# Estimation of the global prevalence of dementia in 2019 and forecasted prevalence in 2050: an analysis for the Global Burden of Disease Study 2019

**DOI:** 10.1016/S2468-2667(21)00249-8

**Published:** 2022-01-06

**Authors:** GBD 2019 Dementia Forecasting Collaborators, Emma Nichols, Jaimie D Steinmetz, Stein Emil Vollset, Kai Fukutaki, Julian Chalek, Foad Abd-Allah, Amir Abdoli, Ahmed Abualhasan, Eman Abu-Gharbieh, Tayyaba Tayyaba Akram, Hanadi Al Hamad, Fares Alahdab, Fahad Mashhour Alanezi, Vahid Alipour, Sami Almustanyir, Hubert Amu, Iman Ansari, Jalal Arabloo, Tahira Ashraf, Thomas Astell-Burt, Getinet Ayano, Jose L Ayuso-Mateos, Atif Amin Baig, Anthony Barnett, Amadou Barrow, Bernhard T Baune, Yannick Béjot, Woldesellassie M Mequanint Bezabhe, Yihienew Mequanint Bezabih, Akshaya Srikanth Bhagavathula, Sonu Bhaskar, Krittika Bhattacharyya, Ali Bijani, Atanu Biswas, Srinivasa Rao Bolla, Archith Boloor, Carol Brayne, Hermann Brenner, Katrin Burkart, Richard A Burns, Luis Alberto Cámera, Chao Cao, Felix Carvalho, Luis F S Castro-de-Araujo, Ferrán Catalá-López, Ester Cerin, Prachi P Chavan, Nicolas Cherbuin, Dinh-Toi Chu, Vera Marisa Costa, Rosa A S Couto, Omid Dadras, Xiaochen Dai, Lalit Dandona, Rakhi Dandona, Vanessa De la Cruz-Góngora, Deepak Dhamnetiya, Diana Dias da Silva, Daniel Diaz, Abdel Douiri, David Edvardsson, Michael Ekholuenetale, Iman El Sayed, Shaimaa I El-Jaafary, Khalil Eskandari, Sharareh Eskandarieh, Saman Esmaeilnejad, Jawad Fares, Andre Faro, Umar Farooque, Valery L Feigin, Xiaoqi Feng, Seyed-Mohammad Fereshtehnejad, Eduarda Fernandes, Pietro Ferrara, Irina Filip, Howard Fillit, Florian Fischer, Shilpa Gaidhane, Lucia Galluzzo, Ahmad Ghashghaee, Nermin Ghith, Alessandro Gialluisi, Syed Amir Gilani, Ionela-Roxana Glavan, Elena V Gnedovskaya, Mahaveer Golechha, Rajeev Gupta, Veer Bala Gupta, Vivek Kumar Gupta, Mohammad Rifat Haider, Brian J Hall, Samer Hamidi, Asif Hanif, Graeme J Hankey, Shafiul Haque, Risky Kusuma Hartono, Ahmed I Hasaballah, M Tasdik Hasan, Amr Hassan, Simon I Hay, Khezar Hayat, Mohamed I Hegazy, Golnaz Heidari, Reza Heidari-Soureshjani, Claudiu Herteliu, Mowafa Househ, Rabia Hussain, Bing-Fang Hwang, Licia Iacoviello, Ivo Iavicoli, Olayinka Stephen Ilesanmi, Irena M Ilic, Milena D Ilic, Seyed Sina Naghibi Irvani, Hiroyasu Iso, Masao Iwagami, Roxana Jabbarinejad, Louis Jacob, Vardhmaan Jain, Sathish Kumar Jayapal, Ranil Jayawardena, Ravi Prakash Jha, Jost B Jonas, Nitin Joseph, Rizwan Kalani, Amit Kandel, Himal Kandel, André Karch, Ayele Semachew Kasa, Gizat M Kassie, Pedram Keshavarz, Moien AB Khan, Mahalaqua Nazli Khatib, Tawfik Ahmed Muthafer Khoja, Jagdish Khubchandani, Min Seo Kim, Yun Jin Kim, Adnan Kisa, Sezer Kisa, Mika Kivimäki, Walter J Koroshetz, Ai Koyanagi, G Anil Kumar, Manasi Kumar, Hassan Mehmood Lak, Matilde Leonardi, Bingyu Li, Stephen S Lim, Xuefeng Liu, Yuewei Liu, Giancarlo Logroscino, Stefan Lorkowski, Giancarlo Lucchetti, Ricardo Lutzky Saute, Francesca Giulia Magnani, Ahmad Azam Malik, João Massano, Man Mohan Mehndiratta, Ritesh G Menezes, Atte Meretoja, Bahram Mohajer, Norlinah Mohamed Ibrahim, Yousef Mohammad, Arif Mohammed, Ali H Mokdad, Stefania Mondello, Mohammad Ali Ali Moni, Md Moniruzzaman, Tilahun Belete Mossie, Gabriele Nagel, Muhammad Naveed, Vinod C Nayak, Sandhya Neupane Kandel, Trang Huyen Nguyen, Bogdan Oancea, Nikita Otstavnov, Stanislav S Otstavnov, Mayowa O Owolabi, Songhomitra Panda-Jonas, Fatemeh Pashazadeh Kan, Maja Pasovic, Urvish K Patel, Mona Pathak, Mario F P Peres, Arokiasamy Perianayagam, Carrie B Peterson, Michael R Phillips, Marina Pinheiro, Michael A Piradov, Constance Dimity Pond, Michele H Potashman, Faheem Hyder Pottoo, Sergio I Prada, Amir Radfar, Alberto Raggi, Fakher Rahim, Mosiur Rahman, Pradhum Ram, Priyanga Ranasinghe, David Laith Rawaf, Salman Rawaf, Nima Rezaei, Aziz Rezapour, Stephen R Robinson, Michele Romoli, Gholamreza Roshandel, Ramesh Sahathevan, Amirhossein Sahebkar, Mohammad Ali Sahraian, Brijesh Sathian, Davide Sattin, Monika Sawhney, Mete Saylan, Silvia Schiavolin, Allen Seylani, Feng Sha, Masood Ali Shaikh, KS Shaji, Mohammed Shannawaz, Jeevan K Shetty, Mika Shigematsu, Jae Il Shin, Rahman Shiri, Diego Augusto Santos Silva, João Pedro Silva, Renata Silva, Jasvinder A Singh, Valentin Yurievich Skryabin, Anna Aleksandrovna Skryabina, Amanda E Smith, Sergey Soshnikov, Emma Elizabeth Spurlock, Dan J Stein, Jing Sun, Rafael Tabarés-Seisdedos, Bhaskar Thakur, Binod Timalsina, Marcos Roberto Tovani-Palone, Bach Xuan Tran, Gebiyaw Wudie Tsegaye, Sahel Valadan Tahbaz, Pascual R Valdez, Narayanaswamy Venketasubramanian, Vasily Vlassov, Giang Thu Vu, Linh Gia Vu, Yuan-Pang Wang, Anders Wimo, Andrea Sylvia Winkler, Lalit Yadav, Seyed Hossein Yahyazadeh Jabbari, Kazumasa Yamagishi, Lin Yang, Yuichiro Yano, Naohiro Yonemoto, Chuanhua Yu, Ismaeel Yunusa, Siddhesh Zadey, Mikhail Sergeevich Zastrozhin, Anasthasia Zastrozhina, Zhi-Jiang Zhang, Christopher J L Murray, Theo Vos

## Abstract

**Background:**

Given the projected trends in population ageing and population growth, the number of people with dementia is expected to increase. In addition, strong evidence has emerged supporting the importance of potentially modifiable risk factors for dementia. Characterising the distribution and magnitude of anticipated growth is crucial for public health planning and resource prioritisation. This study aimed to improve on previous forecasts of dementia prevalence by producing country-level estimates and incorporating information on selected risk factors.

**Methods:**

We forecasted the prevalence of dementia attributable to the three dementia risk factors included in the Global Burden of Diseases, Injuries, and Risk Factors Study (GBD) 2019 (high body-mass index, high fasting plasma glucose, and smoking) from 2019 to 2050, using relative risks and forecasted risk factor prevalence to predict GBD risk-attributable prevalence in 2050 globally and by world region and country. Using linear regression models with education included as an additional predictor, we then forecasted the prevalence of dementia not attributable to GBD risks. To assess the relative contribution of future trends in GBD risk factors, education, population growth, and population ageing, we did a decomposition analysis.

**Findings:**

We estimated that the number of people with dementia would increase from 57·4 (95% uncertainty interval 50·4–65·1) million cases globally in 2019 to 152·8 (130·8–175·9) million cases in 2050. Despite large increases in the projected number of people living with dementia, age-standardised both-sex prevalence remained stable between 2019 and 2050 (global percentage change of 0·1% [–7·5 to 10·8]). We estimated that there were more women with dementia than men with dementia globally in 2019 (female-to-male ratio of 1·69 [1·64–1·73]), and we expect this pattern to continue to 2050 (female-to-male ratio of 1·67 [1·52–1·85]). There was geographical heterogeneity in the projected increases across countries and regions, with the smallest percentage changes in the number of projected dementia cases in high-income Asia Pacific (53% [41–67]) and western Europe (74% [58–90]), and the largest in north Africa and the Middle East (367% [329–403]) and eastern sub-Saharan Africa (357% [323–395]). Projected increases in cases could largely be attributed to population growth and population ageing, although their relative importance varied by world region, with population growth contributing most to the increases in sub-Saharan Africa and population ageing contributing most to the increases in east Asia.

**Interpretation:**

Growth in the number of individuals living with dementia underscores the need for public health planning efforts and policy to address the needs of this group. Country-level estimates can be used to inform national planning efforts and decisions. Multifaceted approaches, including scaling up interventions to address modifiable risk factors and investing in research on biological mechanisms, will be key in addressing the expected increases in the number of individuals affected by dementia.

**Funding:**

Bill & Melinda Gates Foundation and Gates Ventures.

## Introduction

A growing body of evidence from North America and Europe suggests a decreasing trend in dementia incidence, potentially due to increases in educational attainment and improvements in the management of cardiovascular disease and its risk factors.^1^, ^2^, ^3^ It remains unclear whether these trends will continue into the future or whether they extend to other geographical areas, with previous work in Japan and China suggesting increases in age-specific prevalence, which is a function of disease incidence and duration.^4^, ^5^ However, all estimates agree that the absolute number of people affected by dementia will show large increases over time.^6^, ^7^, ^8^, ^9^ Globally, the number of people affected by dementia was estimated to have increased by 117% (95% uncertainty interval [UI] 114–121) between 1990 and 2016, largely due to population ageing.^10^

Demographic analyses suggest that these patterns are driven by decreases in fertility coupled with increases in life expectancy, which together lead to large changes in the age structure of the population (larger numbers of people at the oldest ages than was the case historically).^11^, ^12^ These changes, along with largely stable age-specific prevalence estimates and population growth, lead to large increases in the number of people affected by dementia. Given that these demographic trends are expected to continue into the future, the number of people with dementia will continue to rise.^10^

**Research in context**

**Evidence before this study**

Global efforts to summarise the prevalence and burden of dementia, including the World Dementia Report and the Global Burden of Diseases, Injuries, and Risk Factors Study (GBD), can serve to guide resource allocation and health policy decision making. Additionally, projections of future dementia burden are essential to guide health system planning and inform research funding decisions. We searched PubMed on Oct 23, 2020, using the search terms [“dementia” OR “alzheim*”] AND [“forecast” OR “project*”] AND “prevalence.” Of 635 articles published in English, three reported on global estimates of projected prevalence for dementia or Alzheimer's disease and 30 reported on country-specific estimates. The three studies that calculated global estimates either assumed a constant prevalence and applied population estimates to calculate the effect of changing demographics, or used macrosimulation techniques to estimate prevalence on the basis of transition probabilities for incidence, progression of disease, and mortality. However, none of the available global studies included comprehensive country-level estimates, nor did they consider the potential effects of trends in exposure to known dementia risk factors.

**Added value of this study**

This study leveraged country-specific estimates of dementia prevalence from the GBD study to project dementia prevalence globally, by world region, and at the country level. Furthermore, we incorporated information on projected trends in exposure to known dementia risk factors to understand how trends in risk factors might affect the projected number of individuals with dementia, and did decomposition analysis to understand the drivers of forecasted changes. Our estimates of age-standardised prevalence remained stable between 2019 and 2050 (percentage change of 0·1% [95% uncertainty interval −7·5 to 10·8]), whereas the number of individuals estimated to have dementia increased considerably, from 57·4 (50·4–65·1) million cases in 2019 to 152·8 (130·8–175·9) million cases in 2050. Although we projected increases in the estimated number of individuals with dementia in every country, there was considerable variation, with the highest projected increases in regions of north Africa and the Middle East, and eastern sub-Saharan Africa.

**Implications of all the available evidence**

Due to increases in population growth and population ageing, huge increases in the number of individuals affected by dementia can be expected in 2050. Given that there are currently no available disease-modifying therapies, appropriate emphasis should be placed on efforts to address known modifiable risk factors. Multimodal interventions have shown some success in delaying the rate of cognitive decline and present a promising approach to risk reduction and dementia prevention. Simultaneously, it will be necessary to plan for the expected increases in the utilisation of health and social care services and to expand resources to support caregivers of individuals with dementia. Finally, continued resources should be directed towards better understanding and characterising disease mechanisms, with the goal of developing effective therapeutic agents.

Additionally, evidence has emerged supporting the importance of potentially modifiable risk factors for dementia. The 2020 update to the *Lancet* Commission on dementia prevention, intervention, and care highlighted the evidence for 12 modifiable risk factors for dementia: low education, hypertension, hearing impairment, smoking, midlife obesity, depression, physical inactivity, diabetes, social isolation, excessive alcohol consumption, head injury, and air pollution.^13^ Addressing these factors through public health interventions is a pathway towards reducing disease prevalence, and future changes in modifiable risk factors might influence the trajectory of trends in age-specific prevalence. Studies have hypothesised that education and the prevalence and treatment of cardiovascular disease might be key in explaining previous and future trends in dementia prevalence.^14^, ^15^ Therefore, incorporating the potential effect of changes in risk factors should be a key component of any forecast of dementia prevalence.

The increases over time in the number of people affected by dementia accentuate the importance of not only quantifying the current burden of dementia, but also generating forecasts of the prevalence of dementia to allow for informed policy decisions, health system planning, and allocation of resources. Previous efforts to forecast the prevalence of dementia have largely either been country-specific or have predicted future global cases by simply applying global prevalence estimates to population projections.^16^, ^17^, ^18^, ^19^ Other work has applied macrosimulation techniques, using estimates of incidence, progression, and mortality, although the data requirements to fit these models are large while the evidence base for such data is sparse.^20^

This Article aims firstly to improve and expand on previous forecasting estimates by incorporating the estimated effect of future changes in selected modifiable risk factors and projecting dementia prevalence until 2050 globally, as well as by world region and country, and secondly to decompose the drivers of changes in dementia prevalence. This Article was produced as part of the Global Burden of Diseases, Injuries, and Risk Factors Study (GBD) Collaborator Network and in accordance with the GBD Protocol.

## Methods

### Overview and definitions

The category of dementia, as described in this Article, is referred to under the name Alzheimer's disease and other dementias in GBD 2019. General GBD methods can be found in the GBD 2019 summary papers and in the appendix (pp 10–11).^12^, ^21^, ^22^ The overall GBD protocol and data visualisation tools can be found online. This study complies with the Guidelines for Accurate and Transparent Health Estimates Reporting (GATHER),^23^ and a completed GATHER checklist is available in the appendix (pp 3–4). Flow charts summarising the methodology for the estimation of dementia prevalence from 1990 to 2019 and the projection of dementia prevalence to 2050 are available in the appendix (pp 5–6).

### Prevalence data

The reference case definitions for the estimation of dementia prevalence in the GBD studies were from the Diagnostic and Statistical Manual of Mental Disorders (DSM)-III, DSM-III-R, DSM-IV, or DSM-5, or the International Classification of Diseases (ICD)-8, ICD-9, or ICD-10, assigned by study clinicians in representative surveys.^24^, ^25^ Additional data collected using different definitions or ascertainment methods were included and were adjusted to account for bias due to differences in study characteristics. In GBD 2019, the prevalence of dementia was adjusted downwards to account for dementia that is caused by other clinical diseases (eg, clinical stroke, Parkinson's disease, Down syndrome, and traumatic brain injury).^26^ This correction ensures that the GBD cause list is mutually exclusive. However, dementia as described in this Article does not include this correction, because the future total burden of all dementias is of policy relevance and has been the focus of previous forecasting efforts. Therefore, estimates presented in this Article will not align with those presented in the online tools. The data sources used in this study were identified through systematic review (appendix pp 12–13) and can be downloaded from the Global Health Data Exchange.

We used a Bayesian meta-regression model, MR-BRT (Meta-Regression–Bayesian Regularised Trimmed),^27^ to estimate the sex ratio from sex-specific data and applied this ratio to split data only reported for both sexes combined (34 studies). Additionally, we age-split data with an age range greater than 20 years using the global age pattern, estimated from all data available in narrow age bins (61 studies). To adjust for bias by study definition or characteristics, we used MR-BRT to estimate a network meta-regression model of the bias between the reference case definition and alternative criteria. This modelling framework allows for the inclusion of evidence on the association between different alternative methods rather than limiting the analysis to data on direct comparisons between the reference criteria and each alternative criterion. We modelled the bias associated with studies that used clinical records for diagnosis, studies that used algorithms to classify dementia cases, studies that applied the National Institute on Aging–Alzheimer's Association diagnostic criteria, studies that used general practice records (eg, electronic medical records for diagnosis), and studies that used the 10/66 diagnostic algorithm.^28^, ^29^ We also adjusted for bias in US medical claims data but, because there was evidence of differential bias by age, we used a separate model with a spline on age to appropriately make this adjustment (appendix p 14).

### Estimation of prevalence from 1990 to 2019

We used Disease Modelling Meta-Regression (DisMod-MR) 2.1,^30^ a Bayesian compartmental model widely used in GBD non-fatal modelling, to generate initial estimates of dementia prevalence. This model ensures consistency between the different parameters (ie, prevalence, incidence, remission, mortality) by enforcing associations in a set of differential equations. We assumed zero remission and no incidence before 40 years of age given that dementia is a progressive, chronic disease and is extremely rare before this age.^31^, ^32^ The Bayesian modelling framework allows for the estimation of prevalence in locations with little or no data by leveraging information from covariates as well as priors based on the model fit from the previous higher level of the location hierarchy (ie, the prior for a specific country would be based on the model fit for the region). In locations with high-quality data for both prevalence and incidence, we found that the data on incidence combined with mortality estimates implied a higher prevalence than that observed in the data. Given that dementia incidence is probably measured with less precision because of the gradual onset of disease, we chose to retain prevalence data in our models and exclude data on incidence to improve the estimation of prevalence in this model. Although the measurement of prevalent dementia is not without error, because of the higher average disease severity among prevalent cases than among incident cases, measurement error is probably lower among prevalent cases. In this model, we used covariates of smoking prevalence and average years of education in the population aged 15 years and older to help to guide the model estimation in locations with poor-quality data or no data. We set priors on the value of the education parameter on the basis of the results of a literature meta-analysis on the relative risk of dementia for each additional year of education (appendix p 15). This prevalence model used literature data on mortality to guide initial estimates of dementia mortality, and estimates from this model served as inputs to the mortality modelling process (appendix pp 16–19).

After estimating mortality due to dementia, we ran a second DisMod-MR 2.1 model to calculate final prevalence estimates. This model included the same settings as the first model but excluded literature data on mortality and included our GBD dementia mortality estimates.

### Risk factor forecasts

We initially included the three risk factors (high body-mass index [BMI], high fasting plasma glucose, and smoking) for which sufficient evidence exists to merit inclusion as risk factors for dementia in GBD 2019.^22^, ^33^, ^34^, ^35^ However, various other risk factors have also been proposed for dementia and we tested the association between dementia prevalence and risk factor prevalence for other risks that were included in the 2020 *Lancet* Commission report^13^ and are quantified within the GBD framework (low physical activity, high systolic blood pressure, low education, alcohol use, and air pollution). To quantify risk factors other than education, we used the summary exposure value (SEV), which is a risk-weighted prevalence of a given risk factor exposure, first developed for GBD 2015.^36^ The SEVs range from 0 to 1, with a value of 0 indicating that there is no risk (or no protection for protective risk factors) in a population and a value of 1 indicating that the entire population is at maximum risk.

To forecast SEVs from 2019 to 2050, we calculated the annualised rate of change in logit SEV for each location, age, sex, and past year. We then estimated future annualised rates of change by calculating a weighted mean of prior annualised rates over the time series. A recency weighting parameter altered how much weight is given to more recent trends versus older trends. This parameter was selected using out-of-sample predictive validity holding out the last 10 years of data available. Forecasts for this period were then used to calculate the root-mean square error, which informed the selection of weights. To reduce the effect of extreme trends in long-range forecasts, we implemented caps at the 5th and 95th percentiles of logit SEVs.

We forecasted years of education (up to a maximum of 18 years of education) using similar methods, but assumed that education does not change after 25 years of age and held education forecasts constant after 25 years of age within a given location-sex-specific birth cohort to prevent implausible within-cohort changes. These methods have been previously used in GBD forecasting papers on cause-specific mortality, and additional details can be found in these papers.^37^, ^38^

### Forecasting dementia prevalence attributable to GBD risk factors

We forecasted the combined effect of the three GBD risk factors by calculating a risk factor scalar that describes the proportion of prevalence attributable to GBD risk factors. To calculate our risk factor scalar, we first estimated the population attributable fractions (PAFs) for each risk factor, using past and forecasted SEVs from 1990 to 2050 and relative risks describing the association between each risk factor and dementia, from GBD 2019.^22^ We then estimated the joint PAF for the three risk factors, taking into account potential mediation between the inter-related risk factors. Finally, we calculated scalars specific to each forecasted year, location, age group, and sex as:

$$\text{Scalar}=\frac{1}{1-\text{PAF}}$$

Additional details on the calculation of PAFs, risk factor mediation, and scalars can be found in the appendix (pp 21–22) or in previous GBD forecasting papers.^37^, ^38^

### Forecasting dementia prevalence not attributable to GBD risk factors

We then forecasted GBD risk-deleted prevalence, calculated as the total prevalence divided by the risk factor scalar. To select the additional risk factors to include in our forecasting model, we evaluated the association between each risk factor that was both quantified within the GBD framework and included in the 2020 *Lancet* Commission report^13^ (low physical activity, high systolic blood pressure, low education, alcohol use, and air pollution) and dementia prevalence (logit-transformed). We used sex-stratified models, controlling for 5-year age group and world region. We decided to include covariates for which the estimated association with dementia prevalence in both men and women was significant and in the same direction as the effect reported in the *Lancet* Commission report.^13^ On the basis of these criteria, we added education as a covariate in our forecasting model (appendix p 23).

We used linear regression to forecast logit-transformed GBD risk-deleted prevalence, using sex-stratified models. We included covariates for 5-year age group (reference: 40–44 years), world region (reference: Andean Latin America; appendix p 61), and education (in years), as shown in the equation below depicting the linear regression models:

$$\text{Logit}{\left(\text{prevalence}\right)}_{sex}=\beta_{0}+\beta_{1}\times \text{Education}+\sum_{i=2}^{12}\beta_{i}\times \text{Age}{\text{group}}_{i}+\sum_{i=13}^{33}\beta_{i}\times {\text{Region}}_{i}+ɛ$$

Because our input data on prevalence and risk factors were themselves estimates, we ran 1000 models to incorporate uncertainty from our inputs. Each model used one draw from the distribution of each input to our forecasting model. Finally, to account for trends unexplained by our covariates, we fit a random walk model (autoregressive integrated moving average [ARIMA_(0,1,0)_]) to the residuals of our regression models and added forecasts of these residuals to our estimated forecasts.

### Final prevalence forecasts

We calculated the total forecasted prevalence as the product of our forecasted prevalence not attributable to GBD risk factors and the forecasted risk factor scalar. To ensure continuity between past and future means and UIs, we intercept-shifted the forecasted draws of logit-transformed prevalence. We calculated the number of projected cases of dementia by multiplying prevalence forecasts with population forecasts, as calculated for the GBD population forecasting paper.^38^

### Decomposition of forecasts

To quantify the drivers of changes in the number of individuals with dementia, we estimated the relative contribution of population growth, population age structure, changes due to changes in GBD risk factor prevalence, and changes due to changes in education. For this decomposition analysis, we used methods developed by Das Gupta,^39^ which summarise the contribution of various factors to observed changes by algebraically isolating the standardised impact of each contributing multiplicative factor.

### Quantification of SDI aggregates, age-standardisation, and uncertainty

Socio-demographic Index (SDI) is a composite indicator of total fertility rate, mean years of education in individuals older than 15 years, and lag-distributed income per capita.^12^ Countries were assigned to an SDI quintile on the basis of their estimated values on these indicators in 2019, and these designations were used to produce aggregated estimates of dementia prevalence by SDI quintile. We calculated age-standardised rates using the GBD world population standard.^12^ We propagated uncertainty through all components of the analysis by sampling 1000 draws at each stage of our estimation process. UIs were defined as the 25th and 975th values of the ordered draws. A difference in two quantities was defined as significant if the 95% UI of the difference did not include zero. For our analyses, we used Stata (versions 13.1 and 15.1), Python (versions 3.6.2 and 3.6.8), and R (versions 3.4.2 and 3.5.0).

### Role of the funding source

The study funders had no role in study design, data collection, data analysis, data interpretation, or the writing of the manuscript.

## Results

**Figure 1:**
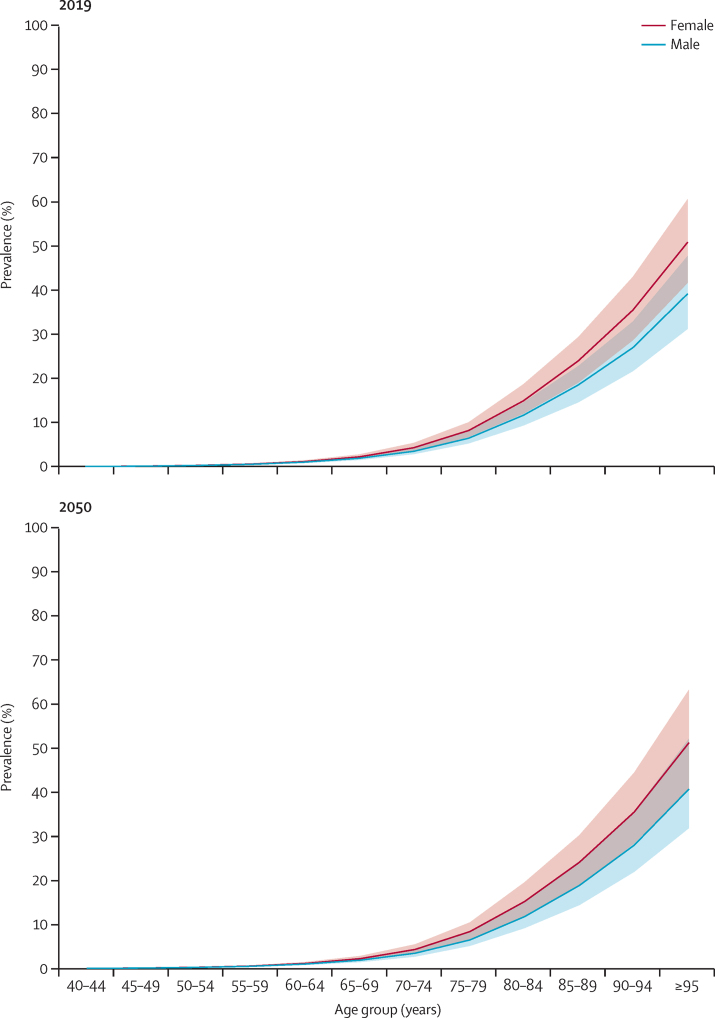
Global prevalence of dementia (with 95% uncertainty intervals) by age group and sex in 2019 and 2050

We estimated a global percentage change in age-standardised both-sex dementia prevalence between 2019 and 2050 of 0·1% (95% UI −7·5 to 10·8), indicating that the global age-sex-specific prevalence of dementia is expected to remain stable. Dementia prevalence was higher in women than in men and increased with age, doubling about every 5 years until 85 years of age in both 2019 and 2050 (figure 1). In 2050, the prevalence of dementia in men globally was forecasted to be 0·5% (95% UI 0·4 to 0·7) among those aged 40–69 years, 6·5% (5·3 to 8·0) among those aged 70–84 years, and 23·5% (19·1 to 29·2) among those aged 85 years and older. In women, the global forecast was projected to be 0·6% (0·5 to 0·8) among those aged 40–69 years, 8·5% (7·0 to 10·4) among those aged 70–84 years, and 30·5% (25·0 to 36·8) among those aged 85 years and older (figure 1).

**Figure 2:**
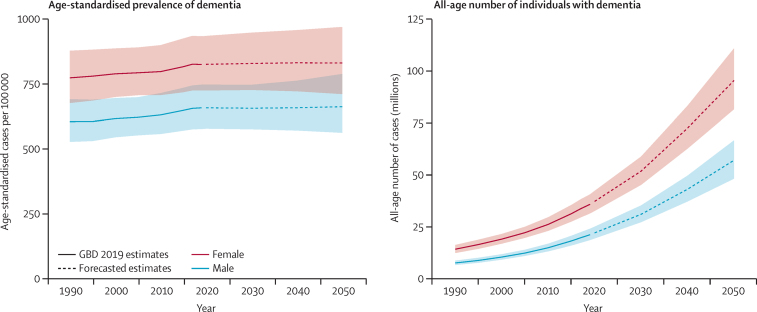
Estimated trends in the global age-standardised dementia prevalence (A) and all-age number of cases (B), with 95% uncertainty intervals, 2019–50 GBD=Global Burden of Diseases, Injuries, and Risk Factors Study.

In comparison, we estimated a much larger percentage change in all-age dementia prevalence between 2019 and 2050 (117% [95% UI 100–133]) due to population ageing, and an even larger percentage change in the all-age numbers of prevalent cases (166% [148–185]), due to a combination of population ageing and population growth (figure 2). In 2019, there were an estimated 57·4 (95% UI 50·4–65·1) million individuals living with dementia globally. We estimated that this number would increase to 83·2 (73·0–94·6) million individuals in 2030, 116·0 (100·7–132·1) million individuals in 2040, and 152·8 (130·8–175·9) million individuals living with dementia in 2050 (table). We estimated that there were more women with dementia than men with dementia globally in 2019 (female-to-male ratio of 1·69 [1·64–1·73]), and we expect this pattern to continue to 2050 (female-to-male ratio of 1·67 [1·52–1·85]).

**Table:** Number of dementia cases in 2019 and 2050 and percentage change in counts and age-standardised rates by country, region, and SDI quintile (95% uncertainty interval)

		**Number of cases**		**Percentage change**	
		2019	2050	Counts	Age-standardised rates
**Global**		**57 394 906 (50 380 728 to 65 059 424)**	**152 811 943 (130 760 492 to 175 873 110)**	**166% (148 to 185)**	**0·1% (−7·5 to 10·8)**
Low SDI		1 216 893 (1 060 042 to 1 393 547)	5 227 425 (4 488 746 to 6 092 498)	330% (295 to 362)	8·2% (0·5 to 19·8)
Low-middle SDI		4 452 767 (3 889 055 to 5 074 679)	15 086 525 (12 932 740 to 17 576 030)	239% (210 to 268)	1·3% (−6·9 to 12·4)
Middle SDI		10 091 960 (8 801 589 to 11 532 222)	34 514 380 (29 282 762 to 40 178 089)	242% (215 to 271)	7·1% (−1·6 to 19·0)
High-middle SDI		9 259 873 (8 039 014 to 10 636 963)	19 700 344 (16 583 929 to 23 011 962)	113% (93 to 134)	1·5% (−5·1 to 9·9)
High SDI		32 373 413 (28 631 545 to 36 610 092)	77 559 727 (65 787 984 to 90 463 680)	140% (117 to 160)	2·8% (−5·7 to 14·0)
Central Asia		436 494 (378 705 to 502 096)	1 501 050 (1 254 725 to 1 773 782)	244% (206 to 280)	10·3% (0·9 to 23·0)
	Armenia	31 890 (27 501 to 36 767)	69 994 (55 443 to 87 569)	119% (84 to 161)	11·6% (1·5 to 24·0)
	Azerbaijan	52 070 (45 063 to 59 823)	195 210 (152 079 to 242 269)	275% (208 to 344)	12·6% (1·8 to 27·0)
	Georgia	46 291 (39 650 to 53 463)	78 458 (53 756 to 107 641)	70% (22 to 126)	10·7% (1·3 to 22·8)
	Kazakhstan	107 698 (93 064 to 123 939)	356 674 (285 733 to 435 149)	231% (181 to 287)	9·8% (−0·1 to 22·2)
	Kyrgyzstan	28 171 (24 481 to 32 273)	105 987 (85 565 to 130 209)	276% (220 to 338)	1·8% (−5·3 to 10·2)
	Mongolia	10 986 (9548 to 12 630)	53 692 (41 881 to 66 919)	389% (300 to 487)	5·4% (−2·0 to 14·9)
	Tajikistan	29 261 (25 457 to 33 337)	135 868 (109 121 to 167 616)	364% (293 to 436)	5·2% (−5·3 to 19·4)
	Turkmenistan	22 117 (19 151 to 25 329)	96 861 (77 795 to 117 753)	338% (268 to 409)	10·0% (−1·6 to 24·9)
	Uzbekistan	108 010 (94 263 to 123 838)	408 306 (307 806 to 515 406)	278% (196 to 357)	14·3% (2·5 to 30·5)
Central Europe		1 969 960 (1 700 079 to 2 274 491)	3 586 187 (2 939 018 to 4 280 177)	82% (60 to 107)	1·4% (−5·1 to 9·6)
	Albania	36 079 (31 092 to 41 597)	86 733 (66 420 to 110 254)	141% (89 to 200)	8·5% (−0·4 to 19·3)
	Bosnia and Herzegovina	48 081 (41 437 to 56 056)	92 167 (72 773 to 116 388)	92% (59 to 130)	7·7% (−2·7 to 20·9)
	Bulgaria	135 285 (116 074 to 156 914)	185 719 (145 989 to 230 430)	37% (14 to 63)	−0·1% (−7·2 to 7·7)
	Croatia	83 429 (71 139 to 97 166)	129 479 (101 243 to 158 966)	55% (32 to 82)	0·8% (−7·7 to 11·2)
	Czech Republic	192 748 (164 883 to 223 173)	379 742 (313 505 to 453 786)	97% (72 to 126)	3·4% (−4·4 to 12·8)
	Hungary	183 870 (157 845 to 213 183)	295 379 (238 639 to 361 018)	61% (38 to 85)	−1·5% (−8·1 to 6·0)
	Montenegro	8247 (7088 to 9583)	14 870 (11 535 to 18 690)	80% (50 to 115)	5·3% (−2·9 to 15·2)
	North Macedonia	28 279 (24 324 to 32 878)	75 147 (62 224 to 89 882)	166% (133 to 205)	7·0% (−1·7 to 18·2)
	Poland	663 408 (573 431 to 766 171)	1 323 739 (1 052 220 to 1 608 391)	100% (70 to 132)	−1·6% (−8·6 to 6·8)
	Romania	341 195 (291 477 to 395 164)	577 177 (466 820 to 696 706)	69% (47 to 93)	3·3% (−5·1 to 12·8)
	Serbia	129 117 (110 961 to 149 416)	178 262 (142 370 to 222 173)	38% (18 to 60)	3·2% (−5·0 to 13·1)
	Slovakia	77 185 (66 652 to 89 480)	163 037 (132 519 to 195 206)	111% (84 to 142)	1·2% (−5·5 to 9·0)
	Slovenia	43 038 (36 793 to 50 186)	84 735 (69 330 to 101 544)	97% (73 to 122)	2·9% (−4·1 to 11·3)
Eastern Europe		2 907 654 (2 509 228 to 3 349 800)	5 591 863 (4 403 039 to 6 883 544)	92% (58 to 127)	3·1% (−2·5 to 9·8)
	Belarus	137 286 (117 896 to 158 209)	245 114 (193 199 to 303 605)	79% (47 to 111)	2·8% (−4·1 to 10·9)
	Estonia	26 710 (22 672 to 31 012)	48 807 (37 612 to 61 074)	83% (50 to 122)	7·7% (−0·5 to 18·1)
	Latvia	38 907 (33 450 to 45 208)	57 136 (42 067 to 74 344)	47% (13 to 85)	3·7% (−3·6 to 12·7)
	Lithuania	56 244 (47 804 to 65 483)	81 237 (63 019 to 101 071)	44% (19 to 70)	0·2% (−6·1 to 7·3)
	Moldova	46 924 (40 603 to 54 020)	119 453 (94 246 to 149 879)	155% (107 to 207)	8·8% (−0·2 to 20·3)
	Russia	1 949 811 (1 679 013 to 2 243 097)	4 032 617 (3 036 325 to 5 108 853)	107% (62 to 155)	4·0% (−2·3 to 11·2)
	Ukraine	651 773 (559 460 to 754 347)	1 007 499 (775 208 to 1 304 708)	55% (24 to 91)	−1·4% (−6·6 to 4·1)
Australasia		414 288 (361 823 to 472 380)	945 658 (780 004 to 1 117 118)	128% (105 to 153)	0·3% (−8·0 to 10·5)
	Australia	348 673 (304 883 to 398 147)	796 702 (656 789 to 944 398)	128% (104 to 153)	−0·2% (−8·5 to 9·9)
	New Zealand	65 616 (56 674 to 75 685)	148 956 (121 688 to 177 915)	127% (103 to 155)	1·0% (−7·2 to 11·3)
High-income Asia Pacific		4 839 076 (4 175 048 to 5 562 598)	7 421 319 (6 290 211 to 8 711 763)	53% (41 to 67)	−10·9% (−16·4 to −4·3)
	Brunei	1574 (1374 to 1795)	7317 (6133 to 8717)	365% (314 to 420)	−7·3% (−15·6 to 2·3)
	Japan	4 117 308 (3 528 130 to 4 752 471)	5 237 201 (4 376 503 to 6 191 162)	27% (16 to 39)	−8·3% (−14·7 to −1·7)
	South Korea	671 288 (593 383 to 759 915)	1 954 016 (1 649 586 to 2 318 128)	191% (157 to 229)	−12·2% (−19·5 to −4·1)
	Singapore	48 906 (43 164 to 55 374)	222 784 (187 231 to 264 640)	356% (303 to 413)	−5·1% (−13·5 to 5·5)
High-income North America		5 856 696 (5 417 356 to 6 362 004)	11 830 056 (10 436 624 to 13 286 322)	102% (83 to 122)	0·5% (−8·5 to 12·1)
	Canada	587 364 (513 628 to 665 177)	1 308 143 (1 092 204 to 1 555 797)	123% (98 to 151)	−2·4% (−11·8 to 10·5)
	Greenland	439 (382 to 508)	1447 (1174 to 1740)	229% (186 to 277)	4·1% (−4·7 to 16·0)
	USA	5 268 893 (4 898 943 to 5 714 140)	10 520 259 (9 316 869 to 11 777 173)	100% (80 to 120)	0·7% (−8·5 to 12·5)
Southern Latin America		634 348 (546 426 to 731 042)	1 468 705 (1 203 709 to 1 775 598)	131% (106 to 160)	4·0% (−5·0 to 15·9)
	Argentina	412 268 (354 720 to 473 783)	892 180 (726 352 to 1 078 782)	116% (89 to 147)	4·3% (−5·5 to 16·8)
	Chile	174 921 (151 480 to 201 357)	491 617 (399 004 to 604 396)	181% (144 to 222)	2·4% (−6·2 to 14·0)
	Uruguay	47 160 (40 364 to 54 718)	84 842 (66 762 to 105 314)	80% (52 to 111)	7·7% (−2·5 to 21·2)
Western Europe		7 860 682 (6 799 340 to 9 053 849)	13 642 589 (11 417 565 to 16 153 469)	74% (58 to 90)	−1·6% (−8·4 to 7·7)
	Andorra	1066 (926 to 1220)	2897 (2376 to 3463)	172% (143 to 200)	0·0% (−7·7 to 10·0)
	Austria	146 391 (125 797 to 168 938)	309 629 (250 432 to 372 437)	111% (89 to 137)	8·4% (−0·8 to 20·5)
	Belgium	190 477 (161 979 to 220 869)	330 572 (272 158 to 399 548)	73% (58 to 93)	−4·5% (−12·2 to 5·5)
	Cyprus	14 143 (12 108 to 16 320)	38 840 (31 358 to 47 469)	175% (139 to 215)	−1·1% (−8·3 to 7·8)
	Denmark	81 923 (70 840 to 93 957)	137 508 (112 574 to 166 054)	68% (48 to 91)	−6·5% (−15·2 to 4·9)
	Finland	97 549 (83 238 to 113 043)	154 002 (126 428 to 184 870)	58% (40 to 79)	−1·5% (−9·3 to 8·3)
	France	1 203 439 (1 032 014 to 1 392 214)	2 191 307 (1 809 791 to 2 657 863)	82% (62 to 102)	1·8% (−7·1 to 13·0)
	Germany	1 691 221 (1 477 093 to 1 944 181)	2 796 783 (2 168 685 to 3 527 605)	65% (35 to 101)	1·8% (−6·5 to 13·5)
	Greece	206 366 (174 643 to 240 487)	298 617 (237 117 to 371 264)	45% (24 to 67)	−1·7% (−10·6 to 9·3)
	Iceland	4222 (3646 to 4842)	10 019 (8368 to 11 863)	137% (115 to 163)	−3·0% (−12·5 to 7·6)
	Ireland	53 932 (46 375 to 62 488)	142 416 (119 268 to 170 671)	164% (136 to 195)	−1·5% (−11·1 to 11·0)
	Israel	85 869 (74 010 to 99 187)	210 339 (173 516 to 250 735)	145% (120 to 171)	−2·2% (−10·7 to 7·7)
	Italy	1 487 368 (1 270 750 to 1 726 466)	2 316 951 (1 895 878 to 2 770 019)	56% (41 to 73)	−5·2% (−12·4 to 3·4)
	Luxembourg	6583 (5708 to 7534)	16 797 (13 947 to 19 856)	155% (131 to 183)	1·2% (−9·0 to 13·7)
	Malta	6651 (5714 to 7728)	11 674 (9515 to 14 061)	76% (53 to 100)	−2·4% (−10·2 to 7·9)
	Netherlands	277 262 (241 522 to 316 994)	493 122 (414 048 to 591 597)	78% (58 to 99)	−6·3% (−14·3 to 3·5)
	Norway	75 301 (65 103 to 86 442)	149 264 (123 996 to 176 637)	98% (80 to 118)	−4·8% (−10·6 to 1·9)
	Portugal	200 994 (171 278 to 233 809)	351 504 (284 708 to 420 991)	75% (55 to 97)	−0·7% (−8·5 to 9·4)
	Spain	826 686 (734 234 to 937 050)	1 516 523 (1 290 677 to 1 764 312)	83% (67 to 101)	−4·4% (−12·5 to 7·2)
	Sweden	153 805 (132 692 to 176 783)	249 290 (208 673 to 298 769)	62% (46 to 79)	−2·3% (−9·8 to 7·4)
	Switzerland	142 105 (121 551 to 164 647)	307 925 (255 333 to 365 367)	117% (99 to 138)	0·3% (−7·4 to 9·7)
	UK	907 331 (784 009 to 1 045 033)	1 592 475 (1 320 026 to 1 912 805)	75% (58 to 95)	−2·4% (−11·7 to 9·1)
Andean Latin America		328 296 (287 528 to 373 369)	1 150 562 (958 542 to 1 376 098)	250% (208 to 294)	7·8% (−0·9 to 19·8)
	Bolivia	43 829 (38 130 to 50 090)	136 892 (112 902 to 164 437)	212% (172 to 260)	6·5% (−2·4 to 18·2)
	Ecuador	87 769 (76 477 to 100 216)	268 822 (211 958 to 329 156)	206% (160 to 255)	5·9% (−3·1 to 17·3)
	Peru	196 699 (172 549 to 224 224)	744 847 (599 223 to 902 865)	279% (222 to 338)	8·8% (−0·7 to 22·1)
Caribbean		291 809 (256 420 to 331 563)	744 761 (619 430 to 885 112)	155% (126 to 186)	2·0% (−5·7 to 11·9)
	Antigua and Barbuda	592 (516 to 673)	1815 (1497 to 2163)	207% (174 to 245)	8·8% (−0·1 to 20·1)
	The Bahamas	1920 (1685 to 2183)	6268 (5081 to 7683)	226% (180 to 277)	2·9% (−4·5 to 12·2)
	Barbados	3023 (2619 to 3476)	5731 (4693 to 6990)	89% (66 to 114)	−0·2% (−6·3 to 7·8)
	Belize	1366 (1193 to 1547)	6213 (5027 to 7505)	355% (293 to 420)	7·5% (−1·4 to 19·8)
	Bermuda	865 (750 to 994)	2066 (1723 to 2437)	139% (113 to 166)	2·4% (−4·7 to 9·9)
	Cuba	116 055 (102 392 to 131 211)	259 547 (206 790 to 320 644)	124% (89 to 165)	−1·9% (−10·3 to 8·1)
	Dominica	574 (497 to 658)	999 (834 to 1194)	74% (56 to 94)	6·3% (−3·0 to 18·6)
	Dominican Republic	51 735 (45 434 to 58 660)	149 178 (119 437 to 184 399)	188% (143 to 239)	7·3% (−4·1 to 22·6)
	Grenada	1058 (900 to 1239)	1743 (1417 to 2102)	65% (46 to 86)	6·4% (−2·9 to 18·3)
	Guyana	2805 (2448 to 3179)	8799 (6635 to 11 290)	214% (143 to 293)	9·8% (0·4 to 22·1)
	Haiti	26 215 (22 676 to 30 173)	83 761 (67 453 to 102 671)	219% (171 to 274)	0·4% (−5·5 to 7·7)
	Jamaica	17 937 (15 630 to 20 448)	40 797 (30 753 to 52 364)	127% (81 to 177)	2·9% (−6·4 to 15·6)
	Puerto Rico	51 313 (44 429 to 58 540)	105 338 (84 370 to 127 371)	105% (75 to 142)	5·1% (−2·7 to 14·3)
	Saint Lucia	1217 (1058 to 1396)	3314 (2693 to 3984)	172% (139 to 209)	1·6% (−5·6 to 11·4)
	Saint Vincent and the Grenadines	830 (723 to 954)	1921 (1550 to 2373)	131% (97 to 170)	10·6% (0·3 to 24·4)
	Suriname	3070 (2673 to 3508)	8843 (6838 to 11 139)	188% (137 to 250)	6·3% (−4·3 to 19·5)
	Trinidad and Tobago	10 194 (8906 to 11 620)	29 968 (22 350 to 38 589)	194% (126 to 267)	3·0% (−4·7 to 11·5)
	Virgin Islands	1039 (899 to 1192)	1680 (1367 to 2052)	62% (39 to 87)	8·3% (−0·4 to 19·5)
Central Latin America		1 336 442 (1 166 913 to 1 523 738)	4 532 279 (3 777 037 to 5 372 328)	239% (202 to 277)	1·7% (−5·6 to 10·6)
	Colombia	369 422 (321 596 to 420 670)	1 375 881 (1 099 923 to 1 666 047)	272% (219 to 333)	3·1% (−5·5 to 13·9)
	Costa Rica	32 637 (28 575 to 37 017)	114 227 (92 564 to 137 653)	250% (200 to 299)	2·4% (−6·1 to 13·0)
	El Salvador	36 675 (31 852 to 42 157)	88 198 (68 336 to 110 623)	140% (92 to 190)	8·4% (−1·2 to 21·8)
	Guatemala	60 721 (52 642 to 70 077)	238 013 (187 528 to 296 739)	292% (221 to 366)	12·2% (0·9 to 28·0)
	Honduras	32 371 (28 168 to 37 050)	109 116 (89 822 to 133 872)	237% (190 to 288)	5·2% (−3·9 to 17·3)
	Mexico	596 202 (519 647 to 681 934)	1 843 049 (1 507 097 to 2 231 411)	209% (168 to 256)	−2·2% (−9·1 to 5·5)
	Nicaragua	28 540 (25 020 to 32 451)	116 953 (96 199 to 138 362)	310% (262 to 361)	6·6% (−3·1 to 18·8)
	Panama	26 527 (23 122 to 30 302)	99 043 (79 933 to 120 285)	273% (221 to 331)	12·0% (−1·3 to 29·2)
	Venezuela	153 345 (134 849 to 173 312)	547 798 (429 118 to 673 874)	257% (193 to 325)	1·3% (−7·3 to 12·2)
Tropical Latin America		1 887 784 (1 663 243 to 2 140 431)	5 786 996 (4 830 039 to 6 888 937)	207% (167 to 251)	−1·6% (−10·6 to 8·9)
	Brazil	1 849 981 (1 630 321 to 2 097 631)	5 666 116 (4 722 302 to 6 738 480)	206% (166 to 251)	−1·9% (−11·0 to 8·6)
	Paraguay	37 803 (32 992 to 43 043)	120 880 (95 942 to 150 443)	220% (162 to 285)	−0·4% (−10·0 to 11·1)
North Africa and Middle East		2 962 640 (2 583 925 to 3 377 445)	13 832 847 (11 816 689 to 15 945 101)	367% (329 to 403)	5·4% (−3·5 to 17·2)
	Afghanistan	55 734 (48 041 to 64 424)	288 376 (237 496 to 350 721)	417% (350 to 485)	18·1% (5·5 to 34·9)
	Algeria	250 509 (216 675 to 286 620)	1 118 628 (928 381 to 1 318 929)	347% (298 to 399)	9·3% (−3·2 to 25·0)
	Bahrain	5126 (4500 to 5832)	60 650 (50 530 to 71 633)	1084% (927 to 1243)	1·2% (−8·1 to 11·4)
	Egypt	305 675 (270 740 to 346 640)	1 530 167 (1 228 884 to 1 858 374)	401% (321 to 482)	10·5% (−1·8 to 26·6)
	Iran	524 457 (455 729 to 600 058)	2 188 336 (1 826 920 to 2 598 034)	317% (274 to 369)	7·3% (−2·8 to 19·5)
	Iraq	159 548 (138 599 to 182 752)	1 050 756 (881 154 to 1 225 100)	559% (483 to 643)	−5·7% (−13·3 to 3·5)
	Jordan	37 373 (32 500 to 43 146)	232 594 (186 892 to 281 471)	522% (437 to 606)	1·6% (−8·4 to 13·2)
	Kuwait	18 001 (15 760 to 20 412)	171 121 (143 406 to 200 674)	850% (754 to 946)	0·4% (−8·4 to 10·9)
	Lebanon	45 952 (40 149 to 52 642)	187 024 (158 959 to 219 882)	307% (269 to 351)	11·5% (0·4 to 25·3)
	Libya	29 286 (25 486 to 33 426)	128 595 (100 400 to 161 805)	339% (264 to 418)	−0·5% (−10·9 to 12·0)
	Morocco	225 682 (196 500 to 260 413)	826 320 (675 460 to 989 097)	266% (220 to 312)	11·0% (−1·0 to 27·2)
	Oman	11 965 (10 398 to 13 630)	124 803 (104 609 to 149 126)	943% (812 to 1080)	5·6% (−6·8 to 22·1)
	Palestine	16 173 (14 130 to 18 487)	79 811 (65 643 to 95 431)	393% (334 to 454)	1·1% (−9·0 to 14·1)
	Qatar	4201 (3665 to 4814)	85 046 (69 847 to 101 841)	1926% (1624 to 2235)	2·2% (−7·8 to 12·8)
	Saudi Arabia	85 735 (74 923 to 97 360)	855 760 (714 919 to 1 021 030)	898% (789 to 1016)	9·4% (−2·3 to 23·3)
	Sudan	115 705 (100 676 to 132 536)	633 251 (521 059 to 754 760)	447% (383 to 511)	15·7% (2·1 to 33·0)
	Syria	85 971 (74 907 to 98 724)	366 323 (296 122 to 440 646)	326% (264 to 388)	−2·4% (−10·8 to 7·4)
	Tunisia	95 059 (82 275 to 109 286)	329 849 (259 371 to 402 301)	247% (195 to 308)	6·0% (−5·3 to 19·8)
	Turkey	803 590 (696 792 to 918 286)	3 031 455 (2 477 722 to 3 603 133)	277% (229 to 330)	0·5% (−9·4 to 11·3)
	United Arab Emirates	11 711 (10 166 to 13 478)	221 672 (174 599 to 283 193)	1795% (1382 to 2265)	0·7% (−9·9 to 12·9)
	Yemen	75 189 (65 552 to 86 510)	309 394 (231 977 to 390 266)	311% (216 to 395)	6·5% (−2·7 to 18·5)
South Asia		4 876 300 (4 220 657 to 5 599 132)	15 070 552 (12 509 797 to 17 939 166)	209% (176 to 245)	6·2% (−2·9 to 19·3)
	Bangladesh	570 899 (496 938 to 659 613)	2 020 081 (1 650 451 to 2 447 563)	254% (205 to 304)	3·7% (−4·7 to 15·3)
	Bhutan	2588 (2244 to 2970)	11 668 (9759 to 13 719)	351% (302 to 403)	9·2% (−0·7 to 24·0)
	India	3 843 118 (3 329 923 to 4 414 367)	11 422 692 (9 367 149 to 13 833 711)	197% (160 to 238)	6·4% (−3·6 to 20·4)
	Nepal	85 635 (74 287 to 98 355)	265 658 (216 849 to 320 886)	210% (169 to 259)	4·0% (−7·8 to 19·6)
	Pakistan	374 060 (326 782 to 429 775)	1 350 453 (1 083 452 to 1 668 674)	261% (205 to 317)	2·7% (−6·5 to 15·8)
East Asia		15 801 493 (13 821 683 to 18 083 556)	47 361 122 (38 293 892 to 56 536 958)	200% (161 to 235)	4·0% (−6·0 to 17·0)
	China	15 330 045 (13 401 824 to 17 569 373)	45 538 093 (36 763 912 to 54 489 693)	197% (159 to 233)	3·7% (−6·6 to 16·9)
	North Korea	192 152 (166 006 to 223 420)	415 335 (323 416 to 531 013)	116% (78 to 165)	−7·0% (−12·5 to −0·3)
	Taiwan (province of China)	279 296 (255 558 to 305 494)	644 733 (547 990 to 755 399)	131% (101 to 161)	−5·7% (−14·4 to 4·0)
Oceania		21 822 (19 048 to 24 912)	84 753 (69 511 to 101 441)	288% (237 to 341)	3·8% (−2·9 to 13·2)
	American Samoa	191 (166 to 220)	622 (509 to 753)	226% (188 to 268)	4·0% (−3·5 to 13·2)
	Fiji	3125 (2719 to 3563)	8107 (6268 to 10 222)	159% (109 to 215)	2·3% (−6·1 to 12·8)
	Guam	1044 (909 to 1194)	2980 (2420 to 3619)	185% (145 to 230)	6·7% (−1·2 to 16·4)
	Kiribati	255 (221 to 293)	772 (617 to 967)	203% (157 to 260)	2·1% (−5·6 to 12·7)
	Marshall Islands	108 (94 to 124)	417 (325 to 522)	285% (214 to 362)	9·5% (0·4 to 21·3)
	Federated States of Micronesia	281 (245 to 320)	904 (728 to 1090)	222% (176 to 269)	3·1% (−5·2 to 13·9)
	Northern Mariana Islands	246 (215 to 279)	1147 (932 to 1381)	366% (303 to 436)	3·6% (−4·1 to 13·4)
	Papua New Guinea	13 460 (11 717 to 15 447)	55 964 (44 852 to 68 164)	316% (251 to 383)	3·9% (−3·2 to 13·7)
	Samoa	692 (602 to 799)	1658 (1318 to 2050)	139% (102 to 183)	−0·2% (−8·4 to 9·5)
	Solomon Islands	1276 (1116 to 1466)	4549 (3702 to 5521)	256% (210 to 305)	6·9% (−2·5 to 19·5)
	Tonga	447 (388 to 515)	1059 (872 to 1276)	137% (107 to 167)	2·9% (−5·6 to 13·5)
	Vanuatu	696 (602 to 805)	1903 (1565 to 2282)	174% (139 to 207)	6·8% (−2·0 to 18·5)
Southeast Asia		3 126 758 (2 736 119 to 3 566 647)	10 574 716 (9 000 551 to 12 307 479)	238% (207 to 269)	4·0% (−4·6 to 17·2)
	Cambodia	55 230 (47 880 to 63 067)	207 476 (173 504 to 246 671)	276% (235 to 320)	7·0% (−4·4 to 22·9)
	Indonesia	987 673 (860 373 to 1 126 148)	3 399 285 (2 879 466 to 3 994 398)	244% (210 to 280)	7·3% (−3·0 to 21·5)
	Laos	19 370 (16 867 to 22 070)	87 292 (72 799 to 104 356)	351% (304 to 400)	10·7% (−0·9 to 26·4)
	Malaysia	142 172 (124 189 to 163 480)	495 842 (411 045 to 596 328)	249% (207 to 293)	1·5% (−8·3 to 13·7)
	Maldives	1703 (1480 to 1948)	11 135 (9290 to 13 157)	554% (486 to 635)	4·8% (−6·0 to 19·8)
	Mauritius	9833 (8638 to 11 249)	27 056 (21 706 to 33 294)	175% (131 to 223)	2·7% (−7·4 to 14·8)
	Myanmar	228 825 (200 081 to 262 541)	726 358 (598 408 to 865 404)	217% (182 to 259)	2·5% (−8·4 to 17·6)
	Philippines	328 852 (286 204 to 375 815)	969 025 (770 021 to 1 223 981)	195% (142 to 251)	−1·8% (−8·9 to 7·1)
	Seychelles	607 (528 to 691)	1598 (1312 to 1915)	163% (135 to 196)	−0·7% (−10·2 to 10·3)
	Sri Lanka	146 778 (127 955 to 168 267)	475 050 (374 248 to 599 464)	224% (166 to 289)	6·9% (−3·6 to 21·3)
	Thailand	670 047 (590 205 to 759 861)	2 391 672 (1 973 046 to 2 864 142)	257% (207 to 311)	2·1% (−8·0 to 15·7)
	Timor-Leste	4036 (3480 to 4639)	12 124 (9963 to 14 527)	201% (160 to 244)	3·2% (−4·9 to 14·5)
	Vietnam	531 633 (462 526 to 604 039)	1 756 890 (1 459 362 to 2 079 348)	230% (194 to 266)	3·1% (−7·0 to 17·1)
Central sub-Saharan Africa		228 263 (198 193 to 261 421)	985 553 (792 176 to 1 192 065)	332% (262 to 402)	3·4% (−3·8 to 13·0)
	Angola	46 598 (40 335 to 53 877)	250 926 (204 456 to 306 412)	439% (357 to 520)	8·3% (−1·5 to 22·3)
	Central African Republic	8207 (7177 to 9434)	24 731 (17 314 to 34 890)	201% (115 to 306)	−1·2% (−7·6 to 6·8)
	Congo (Brazzaville)	11 538 (10 409 to 12 751)	47 842 (35 289 to 64 009)	315% (210 to 431)	1·9% (−8·1 to 14·1)
	DR Congo	153 708 (133 011 to 176 719)	626 362 (471 561 to 787 957)	308% (220 to 396)	1·1% (−6·8 to 10·4)
	Equatorial Guinea	2416 (2086 to 2778)	14 438 (11 553 to 17 658)	498% (399 to 600)	8·0% (−2·7 to 22·2)
	Gabon	5796 (5033 to 6680)	21 254 (17 326 to 25 715)	267% (218 to 317)	6·7% (−4·1 to 20·9)
Eastern sub-Saharan Africa		659 471 (575 759 to 755 115)	3 011 820 (2 579 860 to 3 508 755)	357% (323 to 395)	7·5% (−0·4 to 19·3)
	Burundi	14 791 (12 946 to 16 923)	57 936 (43 752 to 73 814)	292% (212 to 380)	−1·6% (−6·6 to 3·8)
	Comoros	2074 (1810 to 2389)	6757 (5201 to 8596)	226% (161 to 299)	−1·1% (−8·0 to 6·8)
	Djibouti	2085 (1821 to 2383)	11 937 (9646 to 14 655)	473% (382 to 571)	9·2% (−1·1 to 23·1)
	Eritrea	7666 (6628 to 8826)	34 048 (25 632 to 44 102)	344% (244 to 457)	−0·5% (−7·5 to 8·4)
	Ethiopia	174 023 (150 855 to 200 051)	945 224 (796 227 to 1 112 239)	443% (387 to 501)	8·9% (0·9 to 21·5)
	Kenya	86 815 (75 493 to 99 319)	361 042 (299 955 to 429 552)	316% (272 to 365)	3·7% (−5·1 to 15·3)
	Madagascar	37 380 (32 571 to 43 031)	159 725 (122 529 to 205 672)	327% (237 to 425)	1·8% (−6·2 to 11·6)
	Malawi	36 636 (31 524 to 42 617)	132 360 (105 569 to 160 851)	261% (206 to 320)	6·7% (−1·9 to 18·8)
	Mozambique	43 083 (37 458 to 49 507)	181 519 (145 133 to 225 939)	321% (248 to 392)	11·8% (1·3 to 26·5)
	Rwanda	24 705 (21 429 to 28 401)	124 755 (101 000 to 150 739)	405% (330 to 483)	4·8% (−4·0 to 17·2)
	Somalia	21 758 (18 783 to 25 194)	62 012 (45 851 to 80 740)	185% (120 to 259)	−1·1% (−6·0 to 4·3)
	South Sudan	14 107 (12 290 to 16 137)	69 983 (53 255 to 87 649)	396% (297 to 498)	11·4% (3·3 to 21·4)
	Uganda	58 045 (50 363 to 66 863)	273 221 (225 592 to 330 531)	371% (303 to 440)	10·0% (−0·9 to 24·5)
	Tanzania	110 250 (97 219 to 124 082)	469 767 (393 407 to 556 700)	326% (272 to 383)	8·9% (−1·4 to 23·4)
	Zambia	26 055 (22 661 to 29 816)	119 644 (96 970 to 144 675)	359% (296 to 431)	3·6% (−4·6 to 14·6)
Southern sub-Saharan Africa		291 518 (253 868 to 333 957)	830 974 (652 402 to 1 050 035)	185% (133 to 243)	1·7% (−6·2 to 11·4)
	Botswana	6737 (5847 to 7720)	26 873 (21 406 to 33 078)	299% (235 to 364)	6·0% (−5·7 to 19·8)
	Eswatini	2416 (2087 to 2806)	6950 (5149 to 9148)	188% (121 to 264)	0·7% (−7·4 to 10·1)
	Lesotho	5312 (4591 to 6097)	10 726 (7577 to 14 607)	102% (47 to 169)	10·2% (−1·1 to 24·4)
	Namibia	7738 (6686 to 8923)	25 993 (20 376 to 32 595)	236% (172 to 308)	0·1% (−7·9 to 9·7)
	South Africa	241 937 (210 294 to 277 409)	680 045 (514 016 to 882 003)	181% (121 to 250)	1·0% (−7·3 to 11·1)
	Zimbabwe	27 377 (23 716 to 31 626)	80 386 (52 705 to 111 531)	194% (100 to 295)	2·4% (−6·8 to 13·1)
Western sub-Saharan Africa		663 109 (580 172 to 757 219)	2 971 613 (2 479 440 to 3 541 678)	348% (292 to 404)	8·2% (0·1 to 19·1)
	Benin	17 550 (15 411 to 20 147)	76 035 (63 139 to 90 660)	333% (279 to 389)	7·9% (−2·0 to 20·5)
	Burkina Faso	33 793 (29 276 to 38 647)	136 726 (113 069 to 164 640)	305% (252 to 360)	13·2% (3·7 to 27·1)
	Cape Verde	2546 (2197 to 2927)	8324 (6959 to 9857)	227% (192 to 267)	12·2% (−0·7 to 29·2)
	Cameroon	41 181 (35 745 to 47 081)	172 812 (138 989 to 210 888)	320% (255 to 389)	5·8% (−3·2 to 17·2)
	Chad	20 134 (17 569 to 23 061)	75 448 (59 705 to 93 987)	275% (204 to 347)	9·4% (1·3 to 20·1)
	Côte d'Ivoire	34 438 (30 002 to 39 416)	178 367 (143 742 to 216 796)	418% (342 to 489)	8·5% (0·1 to 19·9)
	The Gambia	3796 (3280 to 4390)	12 887 (10 686 to 15 460)	240% (199 to 281)	6·9% (−2·2 to 19·6)
	Ghana	57 276 (49 942 to 65 600)	240 708 (199 701 to 288 987)	320% (271 to 375)	15·0% (2·2 to 31·4)
	Guinea	21 227 (18 523 to 24 293)	61 087 (50 105 to 73 039)	188% (151 to 222)	10·8% (2·4 to 21·7)
	Guinea-Bissau	2271 (1981 to 2619)	9487 (7644 to 11 487)	318% (258 to 377)	5·9% (−1·5 to 15·8)
	Liberia	7084 (6171 to 8125)	31 859 (24 087 to 40 485)	350% (253 to 441)	7·6% (−2·5 to 21·4)
	Mali	31 455 (27 298 to 36 114)	138 631 (114 835 to 166 676)	341% (286 to 400)	11·9% (3·2 to 23·7)
	Mauritania	8404 (7354 to 9634)	34 368 (28 252 to 41 619)	309% (258 to 367)	8·8% (0·6 to 19·3)
	Niger	26 476 (23 097 to 30 336)	122 138 (97 425 to 150 149)	362% (286 to 440)	8·2% (1·7 to 17·2)
	Nigeria	300 332 (262 100 to 341 329)	1 462 514 (1 169 996 to 1 804 298)	387% (300 to 473)	7·1% (−2·1 to 18·0)
	São Tomé and Príncipe	418 (362 to 480)	1535 (1259 to 1850)	267% (220 to 324)	11·1% (−0·1 to 26·1)
	Senegal	29 539 (25 705 to 33 689)	107 492 (88 966 to 129 398)	264% (219 to 310)	7·7% (−0·3 to 18·2)
	Sierra Leone	12 993 (11 252 to 14 966)	45 849 (37 580 to 55 292)	253% (203 to 303)	6·9% (−2·0 to 20·0)
	Togo	12 196 (10 648 to 13 964)	55 318 (44 644 to 66 974)	354% (287 to 425)	4·9% (−3·9 to 16·3)

SDI=Socio-demographic Index.

The percentage change between 2019 and 2050 in total number of dementia cases was highest for the low SDI quintile (330% [95% UI 295 to 362]) and lowest for the high-middle SDI quintile (113% [93 to 134]) and high SDI quintile (140% [117 to 160]). The percentage change in age-standardised rates between 2019 and 2050 was also highest for the low SDI quintile (8·2% [0·5 to 19·8]). The low-middle SDI quintile (1·3% [–6·9 to 12·4]) had the lowest percentage change in age-standardised rates. However, the observed differences between SDI quintiles in percentage change in age-standardised rates were not significant (table).

**Figure 3:**
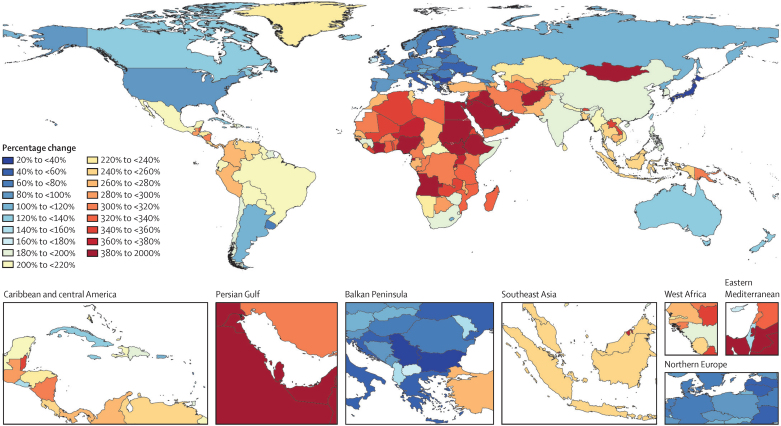
Percentage change between 2019 and 2050 in all-age number of individuals with dementia by country

When examining further patterns of percentage change in the number of projected dementia cases by country or world region, there was evidence of large variability in the projected increases. Although there were increases for every country, the smallest increases were projected in high-income Asia Pacific (53% [95% UI 41–67]) and western Europe (74% [58–90]), whereas the largest estimated increases were in north Africa and the Middle East (367% [329–403]) and eastern sub-Saharan Africa (357% [323–395]; figure 3; table).

**Figure 4:**
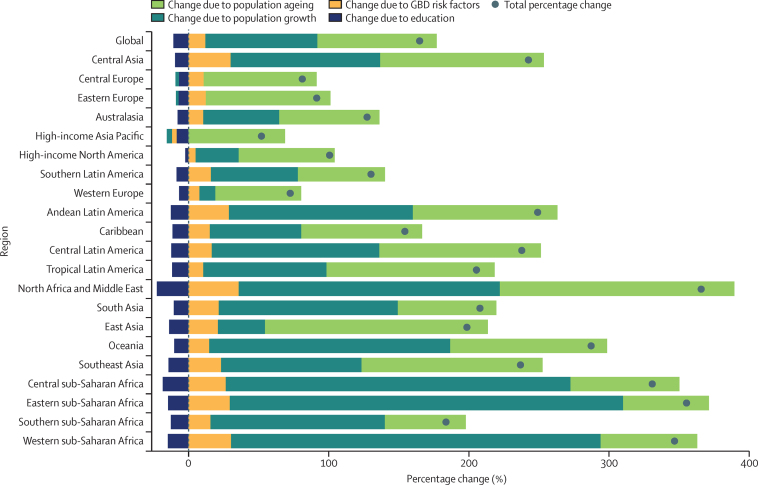
Decomposition of percentage change in the number of individuals with dementia between 2019 and 2050 globally and by world region GBD=Global Burden of Diseases, Injuries, and Risk Factors Study.

The decomposition of percentage change in projected dementia cases indicated a fairly small overall effect of changing prevalence, which included an increase in prevalence due to GBD risk factors for all regions except high-income Asia Pacific, and a decrease in prevalence due to the added effect of trends in education in this analysis. The increases attributable to GBD risk factors in most regions included the effects of increases in BMI and fasting plasma glucose, as well as less clear trends in smoking. Declines in dementia prevalence due to GBD risk factors in high-income Asia Pacific stem from expected declines in risk factor prevalence in this region. However, these effects were overwhelmed by changes due to population ageing and growth. Although at the global level population ageing and population growth contributed about equally to forecasted increases in dementia prevalence, we observed different patterns at the regional level. Whereas population growth is predicted to drive most of the increases in dementia prevalence in sub-Saharan Africa, population growth contributes to a much lesser extent in east Asia and western Europe. In central Europe, eastern Europe, and high-income Asia Pacific, anticipated declines in populations serve to partly counteract the effect of population ageing. Although population ageing is expected to lead to increases in projected dementia cases in every region, the largest projected increases due to population ageing are observed in east Asia, and north Africa and the Middle East (figure 4).

## Discussion

Most of the projected increase in the numbers of people living with dementia can be attributed to population ageing and population growth, although the relative contribution of these two factors varied across GBD world regions. By contrast, we found that country-level projected changes in age-specific or age-standardised dementia prevalence attributable to changes over time in risk factor prevalence were fairly small. In most regions, increases in age-standardised dementia prevalence due to increases in the prevalence of GBD risk factors, including BMI, fasting plasma glucose, and smoking, were counterbalanced with decreases in age-standardised prevalence due to increases in average educational attainment.

Our estimate for the global number of dementia cases in 2050 is similar to previous estimates from the World Alzheimer Report 2019 (152 million cases),^40^ but much higher than previous estimates of the forecasted global prevalence from work done by Brookmeyer and colleagues (106·8 million cases [95% UI 47·2–221·2]).^20^ The estimates from Brookmeyer and colleagues were only for Alzheimer's disease, which constitutes a subset of the total prevalence of dementia, and therefore it is expected that these estimates would be lower than the ones presented here. Due to the high prevalence of mixed pathologies among dementia cases and potential unknown differences in the distribution of underlying causes of dementia across geographical locations, the larger total dementia category is more relevant for the global study of dementia.^41^

Consistent with previous reports,^10^, ^17^ we estimated that there were more women with dementia than men with dementia in 2019, and our analysis indicated that this pattern would continue in 2050. These patterns exist despite the larger prevalence of vascular risks in men than in women, suggesting potentially strong counteracting mechanisms driving these inequities. Although the sex difference can be explained in part by higher life expectancy in women than in men, previous evidence also suggests potential sex differences in the biological mechanisms that underlie Alzheimer's disease.^42^, ^43^

We predicted that population ageing and population growth will drive enormous increases in the number of individuals affected by dementia both regionally and globally. The consequences of these increases are compounded by the high societal and monetary costs of the disease.^44^, ^45^ Given the current absence of available effective disease-modifying treatments for dementia, immediate efforts to reduce these projected increases will need to target disease prevention through interventions for modifiable risk factors.^46^, ^47^ Our analysis suggested that there would not be large changes in age-specific dementia prevalence due to changes in risk factor prevalence, based on the continuation of previous trends in the population-level exposure to educational attainment, BMI, fasting plasma glucose, and smoking. However, interventions that alter the expected trends in risk factor prevalence might reduce the expected future prevalence of dementia. Results from the 2020 update to the *Lancet* Commission on dementia prevention, intervention, and care suggest that up to 40% of dementia prevalence might be preventable through interventions targeting modifiable risk factors.^13^ This conclusion suggests that large changes in the exposure distributions of modifiable risk factors (ie, decreases in harmful risk factors and increases in protective factors) have the potential to considerably change our forecasted estimates and reduce the future burden of disease.

The 2019 guidelines on dementia risk reduction from WHO further highlight potential interventions that might help to reduce the projected increases in dementia prevalence.^48^ However, these guidelines focus primarily on individual-level interventions. Our results indicate that there are differences in the estimated change in age-standardised prevalence rates across SDI quintiles, and suggest that structural inequalities must also be addressed to tackle the observed disparities.

Our estimates can be used to plan for increases in the anticipated resources and support that will be needed by individuals with dementia and their caregivers in the future. Currently, many shortcomings exist in the identification, treatment, and care of individuals with dementia globally. Lower diagnosis rates in low-income settings than in high-income settings and gaps in the availability of resources for the management of neuropsychiatric symptoms of dementia will be important to address.^49^, ^50^, ^51^ Evidence suggests that hospitalisation rates are higher among those with dementia than among those without dementia, and health systems need to be prepared for the expected increases in service use that will accompany increases in the number of people with dementia.^52^ High rates of comorbidity with many other chronic diseases further complicate the care required for individuals with dementia and underscore the need to appropriately plan for the health-care needs of this population.^53^ Furthermore, although large gaps currently exist in the availability of quality end-of-life care for individuals with dementia, such care can positively affect both individuals with dementia and their caregivers.^54^, ^55^ Therefore, efforts should be made to provide these services and scale up available resources to meet the needs of individuals with dementia in the future. The effect on the increasing numbers of caregivers that will be needed to support growing numbers of people with dementia should also be considered, and evidence-based interventions to support caregivers should be part of any comprehensive plan to address increases in dementia prevalence.^56^

Our study had some limitations. The methodology for forecasting prevalence due to GBD risk factors using PAFs relies on the assumptions of the PAF calculation—in particular, the assumption that risks examined are causally related to dementia. To the extent that the estimated relative risks reflect non-causal increases or decreases in risk, this assumption might be violated. Our estimates of dementia prevalence from the GBD study are based on sparse and heterogeneous data because of the absence of standardised methodologies for measuring dementia in population-based studies.^10^ When no data existed, we relied on the Bayesian framework of the DisMod-MR 2.1 and assumed that prevalence could be explained using regional estimates of prevalence adjusted by covariate values. Although there is no available gold standard to which we can judge the validity of our estimates, our global estimates are very similar to those from other efforts, such as the World Alzheimer Report.^40^ Furthermore, some of the geographical heterogeneity observed could be due to methodological differences in prevalence studies, even though we attempted to adjust for studies using different methodologies and different definitions of dementia. Due to the heterogeneity in prevalence data over time, we did not allow observed time trends to affect our forecasted time trends in the future, because this could have induced spurious trends caused by changes in diagnostic procedures over time. Instead, our future time trends are based solely on expected trends in risk factor prevalence, as well as trends in population ageing and growth. Further investments in high-quality epidemiological studies are required to better estimate trends over time in dementia incidence and prevalence as well as geographical variation in dementia prevalence. This observed heterogeneity in prevalence data, in combination with high correlations between GBD risk factors and other dementia risk factors, could have had a role in our ability to detect a significant association between risk factors of dementia that are not currently included in the GBD study and GBD risk-deleted dementia prevalence by country. This limited the number of risk factors we could include as predictors of future dementia prevalence as well as the risk factors we could include in the decomposition analysis showing the effect of risks on future prevalence. The number of risk factors we tested for inclusion was additionally limited to include only those risk factors that were included in the 2020 *Lancet* Commission report and are part of the GBD comparative risk assessment framework.^13^, ^22^ Despite this limitation, we were able to include risks for which large changes in the prevalence of exposure are expected, including BMI, educational attainment, and fasting plasma glucose.^57^, ^58^, ^59^ These risks are of most importance to future forecasts, which will only be affected by the exclusion of risk factors if the future exposure to a given risk is expected to change. However, future efforts should seek to incorporate more risks within GBD, which would allow us to include additional risk factors without relying on the estimation of the ecological association between risk exposure prevalence and dementia prevalence.

We also assumed that the maximal educational attainment was 18 years of education and that educational attainment was constant after the age of 25 years, as higher levels of education are probably not consequential for most health outcomes quantified in the GBD project. However, continued education throughout the life course might convey a greater protective benefit for dementia.^60^ Furthermore, our exposure estimates for high BMI are based on the distribution of BMI in a population at a specific timepoint, but the evidence for BMI as a risk factor suggests that midlife BMI is most important. Therefore, future efforts should consider lagging BMI exposure to capture the risk–outcome association in forecasts more accurately. Additionally, this study focused on the overall prevalence of dementia because of a scarcity of globally relevant data on the decomposition of the broader dementia category into clinical subtypes. It is possible that clinical subtypes, such as vascular dementia, might have different underlying associations with dementia risk factors, which could affect our results. However, evidence suggesting a large overlap of neuropathologies across clinical subtypes calls into question the precision or even utility of clinical subtype designations.^41^, ^61^ Our study also focused on the estimation of dementia prevalence, as the estimation of the numbers of individuals with dementia is of direct relevance for public health planning efforts. However, the estimation of future dementia incidence is also useful and should be the topic of future investigations.

Despite these limitations, this study improves on previous global forecasts of dementia in several important ways. First, drawing on the strength of the country-level estimates from GBD 2019, we were able to produce estimates of projected prevalence by country, world region, and SDI quintile, whereas previous studies have been restricted to global and regional estimates. Second, we used country-level forecasts of four different risk factors that have been shown to be associated with dementia, and the observed association between these risks and dementia prevalence from 1990 to 2019, to incorporate changes in prevalence that would be due to changes in these risk factors. Third, we were able to quantify through decomposition analysis the relative contribution of changes in GBD risk factors, changes in education, changes in population growth, and changes in population ageing.

The country-level specificity of our estimates will allow policy makers and decision makers to understand the expected increases in the number of individuals with dementia and the drivers of these increases in a given geographical setting. This information might be helpful for public health planning efforts, particularly as they relate to scaling up the availability of resources required to meet the needs of individuals with dementia and their caregivers. Furthermore, the projected increases in the number of people with dementia, due largely to population growth and population ageing, underscore the crucial need for research focused on the discovery of disease-modifying treatments, effective low-cost interventions, and novel modifiable risk factors for the prevention or delay of disease onset.

Correspondence to: Emma Nichols, Institute for Health Metrics and Evaluation, University of Washington, Seattle, WA 98105, USA **eln1@uw.edu**

## Data sharing

Citations for the data used in the study can be accessed from the Global Health Data Exchange (http://internal-ghdx.healthdata.org/). Access to the data is also provided as a data use agreements permit.

## Declaration of interests

Y Bejot reports honoraria for lectures from Boehringer-Ingelheim, Bristol Myers Squibb, Pfizer, Servier, Medtronic, and Amgen, outside the submitted work. C Brayne reports support for the present manuscript from the Economic and Social Research Council (ESRC), Alzheimer's Research UK, National Institute for Health Research (NIHR), Medical Research Council (MRC), Alzheimer's Society, East Anglia Regional Health Authority Public Health and Operational Research Advisory Council, Regional Health Authority (as research grants from 1990 to date for the Cognitive Function and Ageing Studies), paid to their institution; honoraria for lectures, presentations, speakers' bureaus, manuscript writing, or educational events from the National Institute on Aging (NIA) Health and Retirement Study (HRS) Data Monitoring Committee and AXA Research Fund Scientific Board, paid to their institution, and from the Department of Biotechnology and Wellcome Trust India Alliance Fellowship Selection Committee, as a personal payment; support for attending meetings and travel from NIA HRS Data Monitoring Committee, Department of Biotechnology and Wellcome Trust India Alliance Fellowship Selection Committee, Canadian Longitudinal Study on Aging Scientific Advisory Board, Alzheimer's Society Research Strategy Council, BRAIN & HEADING International Oversight Committee, The Irish Longitudinal Study on Aging (TILDA) Scientific Advisory Board, ATHLOS Advisory Board, Barcelona Brain Health Initiative Scientific Advisory Board, DZNE International Scientific Review Panel (Humboldt), Faculty of Public Health Academic & Research Committee, and Faculty of Public Health Board; participation on a Data Safety Monitoring Board or Advisory Board with NIA HRS Data Monitoring Committee, AXA Research Fund Scientific Board, Department of Biotechnology and Wellcome Trust India Alliance Fellows, Canadian Longitudinal Study on Aging Scientific Advisory Board, Alzheimer's Society Research Strategy Council, BRAIN & HEADING International Oversight Committee, TILDA Scientific Advisory Board, Chinese University of Hong Kong Project Advisory Board, University of Sheffield Health Lifespan Institute Advisory Board, ATHLOS Advisory Board, Barcelona Brain Health Initiative Scientific Advisory Board, DZNE International Scientific Review Panel (Humboldt), Scientific Advisory Board for UKPRP Air Pollution and Cognitive Health Consortium, and InSPIRE; leadership or fiduciary role in other board, society, committee, or advocacy group, paid or unpaid, with Faculty of Public Health Academic & Research Committee as Chair, Faculty of Public Health Board as a trustee, Public Health England–University of Cambridge Academic Liaison Committee meeting as Chair, and East of England Public Health England Research and Evaluation Hub as co-Chair; all outside the submitted work. I Filip reports payment or honoraria for lectures, presentations, speakers' bureaus, manuscript writing, or educational events from Avicenna Medical and Clinical Research Institute in the form of financial support, outside the submitted work. B J Hall reports consulting fees from WHO, and holds a US S&P index fund and a US Bond Index fund, all outside the submitted work. C Herteliu reports grants from the Romanian National Authority for Scientific Research and Innovation (CNDS-UEFISCDI, project number PN-III-P4-ID-PCCF-2016-0084), outside the submitted work. A Kandel reports grants from the University at Buffalo Clinical and Translational Institute. M Kivimäki reports support for the present manuscript from the MRC (S011676) and the Wellcome Trust (221854/Z/20/Z) as a grant paid to their institution. S Lorkowski reports grants or contracts from Akcea Therapeutics as payments made to their institution; consulting fees from Danone, Swedish Orphan Biovitrum (SOBI), and Upfield; payment or honoraria for lectures, presentations, speakers' bureaus, manuscript writing, or educational events from Akcea Therapeutics, AMARIN, Amedes Holding, Amgen, Berlin-Chemie, Boehringer-Ingelheim Pharma, Daiichi Sankyo Deutschland, Danone, Hubert Burda Media Holding, Lilly Deutschland, Novo Nordisk Pharma, Roche Pharma, Sanofi-Aventis, and SYNLAB Holding and SYNLAB Akademie as personal payments; support for attending meetings and travel from Amgen as personal payments; participation on a Data Safety Monitoring Board or Advisory Board with Akcea Therapeutics, Amgen, Daiichi Sankyo, and Sanofi-Aventis as personal payments; all outside the submitted work. J Massano reports consulting fees from Roche, Biogen, Bial, and AbbVie; payment or honoraria for lectures, presentations, speakers' bureaus, manuscript writing, or educational events from Bial, GE Healthcare, Boston Scientific, and Merck Sharp & Dohme; support for attending meetings and travel from Bial and Roche; leadership or fiduciary role in other board, society, committee, or advocacy group, paid or unpaid, with the Portuguese Brain Aging and Dementia Study Group as President; all outside the submitted work. C D Pond reports payment or honoraria for lectures, presentations, speakers' bureaus, manuscript writing, or educational events from Dementia Training Australia; leadership or fiduciary role in other board, society, committee, or advocacy group, unpaid, as an advisor for the Primary Health Network. A Radfar reports payment or honoraria for lectures, presentations, speakers' bureaus, manuscript writing, or educational events from Avicenna Medical and Clinical Research Institute. J A Singh reports consulting fees from Crealta–Horizon, Medisys, Fidia, Two Labs, Adept Field Solutions, Clinical Care options, Clearview healthcare partners, Putnam associates, Focus forward, Navigant consulting, Spherix, MedIQ, UBM, Trio Health, Medscape, WebMD, Practice Point communications, the NIH, and the American College of Rheumatology; payment or honoraria for lectures, presentations, speakers' bureaus, manuscript writing, or educational events from Simply Speaking; support for attending meetings and travel from OMERACT, an international organisation that develops measures for clinical trials and receives arm's length funding from 12 pharmaceutical companies; participation on a Data Safety Monitoring Board or Advisory Board as a member of the Food and Drug Administration Arthritis Advisory Committee; leadership or fiduciary role in other board, society, committee, or advocacy group, paid or unpaid, with OMERACT as a member of the steering committee, with the Veterans Affairs Rheumatology Field Advisory Committee as a member, and with the UAB Cochrane Musculoskeletal Group Satellite Center on Network Meta-analysis as a director and editor; stock or stock options in TPT Global Tech, Vaxart pharmaceuticals, Charlotte's Web Holdings, and previously owned stock options in Amarin, Viking, and Moderna pharmaceuticals; all outside the submitted work. D J Stein reports personal fees from Lundbeck, Takeda, Johnson & Johnson, and Servier, outside the submitted work. A Wimo reports support for the present manuscript from WHO as payment to their institution and from the Swedish Government (SNAC project) paid to their county council; grants or contracts from Merck Sharp & Dohme (research grant, EU-project IMI2: MOPEAD, EU-project H2020; PRODEMOS, EU-project JPND: MindAD), paid to their institution; royalties and licenses with an RUD instrument as a partial license holder; support for attending meetings and travel to Geneva from WHO, and to Seattle, WA, USA, from the Institute of Health Metrics and Evaluation (IHME); participation on a Data Safety Monitoring Board or Advisory Board with Biogen, Elsai, and IHME; all outside the submitted work.
