# Supplementary material for: Estimation of the global prevalence of dementia in 2019 and forecasted prevalence in 2050: an analysis for the Global Burden of Disease Study 2019
Source: Lancet Public Health. 2022 Jan 6;7(2):e105–25. doi: 10.1016/S2468-2667(21)00249-8 (PMC8810394; doi:10.1016/S2468-2667(21)00249-8)
Supplement: Supplementary appendix [file mmc1.pdf]

THE LANCET
Public Health

Supplementary appendix

This appendix formed part of the original submission and has been peer reviewed. We post it as supplied by the authors.

Supplement to: GBD 2019 Dementia Forecasting Collaborators. Estimation of the global prevalence of dementia in 2019 and forecasted prevalence in 2050: an analysis for the Global Burden of Disease Study 2019. *Lancet Public Health* 2022; published online Jan 6. https://doi.org/10.1016/S2468-2667(21)00249-8.

Appendix

Table of Contents

GATHER checklist, with description of compliance and location of information 3
Author Contributions 5
Dementia prevalence flowchart 8
Dementia forecasting flowchart 9
General methods for the Global Burden of Disease (GBD) study 10
Methods for the estimation of dementia 12
Case definition 12
Input data for prevalence estimation 12
Modelling strategy for prevalence estimation 13
Input data for mortality estimation 16
Modelling strategy for mortality estimation 17
Methods for the calculation of SEV scalars 20
Methods for the calculation of population attributable fractions (PAFs), PAF mediation, and risk factor scalars 21
Selection of risk factors for final forecasting model 23
Effects of age group and risk factors in full forecasting model 24
Air pollution estimation methods 25
Flowchart 25
Input data and modelling strategy 25
Modelling strategy 27
Alcohol estimation methods 30
Flowchart 30
Input data and methodological summary 30
Modelling strategy 33
References 34
Body mass index estimation methods 36
Flowchart 36
Input data and methodological summary 36
Modelling strategy 38
Education estimation methods 42
References 43

High LDL cholesterol estimation methods 44
Flowchart 44
Input data and methodological summary 44
Modelling strategy 46
References 48
High systolic blood pressure estimation methods 49
Flowchart 49
Input data and methodological summary 49
Modelling 51
Smoking estimation methods 55
Flowchart 55
Input data and methodological summary 55
Modelling strategy 56
References 58
Estimation of relative risks for GBD dementia risk factors 59
High fasting-plasma glucose 59
High body-mass index 59
Smoking 59
GBD world regions 61
Supplemental Figure S1 62
Supplemental Figure S2 63

GATHER checklist, with description of compliance and location of information

#	GATHER checklist item	Description of compliance	Reference
**Objectives and funding**			
1	Define the indicators, populations, and time periods for which estimates were made.	Narrative provided in paper and appendix describing indicators, definitions, and populations	Main text (Methods) and appendix
2	List the funding sources for the work.	Funding sources listed in paper	Summary (Funding)
**Data Inputs**			
For all data inputs from multiple sources that are synthesised as part of the study:			
3	Describe how the data were identified and how the data were accessed.	Narrative description of data seeking methods provided	Main text (Methods) and appendix
4	Specify the inclusion and exclusion criteria. Identify all ad-hoc exclusions.	Narrative about inclusion and exclusion criteria by data type provided; ad hoc exclusions in cause-specific write-ups	Main text (Methods) and appendix
5	Provide information on all included data sources and their main characteristics. For each data source used, report reference information or contact name/institution, population represented, data collection method, year(s) of data collection, sex and age range, diagnostic criteria or measurement method, and sample size, as relevant.	An interactive, online data source tool that provides metadata for data sources by component, geography, cause, risk, or impairment has been developed	Online data citation tools: http://ghdx.healthdata.org/gbd-2019/data-input-sources
6	Identify and describe any categories of input data that have potentially important biases (e.g., based on characteristics listed in item 5).	Summary of known biases by cause included in appendix	Appendix
For data inputs that contribute to the analysis but were not synthesised as part of the study:			
7	Describe and give sources for any other data inputs.	Included in online data source tool	http://ghdx.healthdata.org/gbd-2019/data-input-sources
For all data inputs:			
8	Provide all data inputs in a file format from which data can be efficiently extracted (e.g., a spreadsheet as opposed to a PDF), including all relevant meta-data listed in item 5. For any data inputs that cannot be shared due to ethical or legal reasons, such as third-party ownership, provide a contact name or the name of the institution that retains the right to the data.	Downloads of input data available through online data tools (visualization/ data query, GHDx); input data not in tools will be made available upon request	Online data visualisation tools, data query tools, and the Global Health Data Exchange
**Data analysis**			
9	Provide a conceptual overview of the data analysis method. A diagram may be helpful.	Methods section has overview of analysis methods; A flow diagram of the dementia modelling process has been provided	Main text (Methods) and appendix
10	Provide a detailed description of all steps of the analysis, including mathematical formulae. This description should cover, as relevant, data cleaning, data pre-processing, data adjustments and weighting of data sources, and mathematical or statistical model(s).	Flow diagrams and methods write-ups have been provided	Main text (Methods) and appendix

11	Describe how candidate models were evaluated and how the final model(s) were selected.	Provided in methodological write-ups	Appendix
12	Provide the results of an evaluation of model performance, if done, as well as the results of any relevant sensitivity analysis.	Provided in methodological write-ups	Appendix
13	Describe methods for calculating uncertainty of the estimates. State which sources of uncertainty were, and were not, accounted for in the uncertainty analysis.	Appendix	Appendix
14	State how analytic or statistical source code used to generate estimates can be accessed.	Appendix	http://github.com/ihmeuw
**Results and Discussion**			
15	Provide published estimates in a file format from which data can be efficiently extracted.	GBD 2019 results available through online data tools, the Global Health Data Exchange, and online data query tool	Main text, appendix, online data tools (visualization/ data query tools, GHDx)
16	Report a quantitative measure of the uncertainty of the estimates (e.g. uncertainty intervals).	Uncertainty provided with all results	Main text, appendix, online data tools (visualization/ data query tools, GHDx)

Author Contributions

Managing the estimation or publications process
Stein Emil Vollset and Theo Vos.

Writing the first draft of the manuscript
Emma Nichols.

Primary responsibility for applying analytical methods to produce estimates
Emma Nichols, Jaimie D Steinmetz, Kai Fukutaki, and Julian Chalek.

Primary responsibility for seeking, cataloguing, extracting, or cleaning data; designing or coding figures and tables
Emma Nichols and Jaimie D Steinmetz.

Providing data or critical feedback on data sources
Jaimie D Steinmetz, Stein Emil Vollset, Amir Abdoli, Ahmed Abualhasan, Tayyaba Akram, Hanadi Al Hamad, Fares Alahdab, Fahad Mashhour Alanezi, Sami Almustanyir, Hubert Amu, Iman Ansari, Jalal Arabloo, Tahira Ashraf, Thomas Astell-Burt, Atif Amin Baig, Amadou Barrow, Yannick Béjot, Srinivasa Rao Bolla, Archith Boloor, Carol Brayne, Katrin Burkart, Luis Alberto Cámera, Ferrán Catalá-López, Dinh-Toi Chu, Vera Marisa Costa, Rosa A S Couto, Omid Dadras, Lalit Dandona, Rakhi Dandona, Diana Dias da Silva, Abdel Douiri, David Edvardsson, Michael Ekholuenetale, Khalil Eskandari, Jawad Fares, Umar Farooque, Xiaoqi Feng, Seyed-Mohammad Fereshtehnejad, Shilpa Gaidhane, Ahmad Ghashghaee, Syed Amir Gilani, Mahaveer Golechha, Veer Bala Gupta, Vivek Kumar Gupta, Brian J Hall, Shafiul Haque, Simon I Hay, Mohamed I Hegazy, Reza Heidari-Soureshjani, Claudiu Herteliu, Mowafa Househ, Rabia Hussain, Seyed Sina Naghibi Irvani, Vardhmaan Jain, Jost B Jonas, Rizwan Kalani, André Karch, Gizat M Kassie, Moien AB Khan, Mahalaqua Nazli Khatib, Tawfik Ahmed Muthafer Khoja, Jagdish Khubchandani, Min Seo Kim, Yun Jin Kim, Adnan Kisa, Sezer Kisa, G Anil Kumar, Manasi Kumar, Xuefeng Liu, Stefan Lorkowski, Man Mohan Mehndiratta, Ritesh G Menezes, Atte Meretoja, Norlinah Mohamed Ibrahim, Ali H Mokdad, Mohammad Ali Moni, Tilahun Belete Mossie, Gabriele Nagel, Trang Huyen Nguyen, Bogdan Oancea, Mayowa O Owolabi, Songhomitra Panda-Jonas, Fatemeh Pashazadeh Kan, Maja Pasovic, Urvish K Patel, Mona Pathak, Mario F P Peres, Arokiasamy Perianayagam, Carrie B Peterson, Marina Pinheiro, Michele H Potashman, Sergio I Prada, Fakher Rahim, David Laith Rawaf, Salman Rawaf, Nima Rezaei, Brijesh Sathian, Monika Sawhney, Mete Saylan, Allen Seylani, Feng Sha, Masood Ali Shaikh, Mohammed Shannawaz, Jeevan K Shetty, Jae Il Shin, Jasvinder A Singh, Valentin Yurievich Skryabin, Anna Aleksandrovna Skryabina, Sergey Soshnikov, Jing Sun, Rafael Tabarés-Seisdedos, Bhaskar Thakur, Marcos Roberto Tovani-Palone, Bach Xuan Tran, Gebiyaw Wudie Tsegaye, Sahel Valadan Tahbaz, Pascual R Valdez, Narayanaswamy Venketasubramanian, Vasily Vlassov, Giang Thu Vu, Linh Gia Vu, Seyed Hossein Yahyazadeh Jabbari, Naohiro Yonemoto, Chuanhua Yu, Ismaeel Yunusa, Siddhesh Zadey, Mikhail Sergeevich Zastrozhin, Anasthasia Zastrozhina, Christopher J L Murray, and Theo Vos.

Developing methods or computational machinery
Emma Nichols, Stein Emil Vollset, Kai Fukutaki, Tayyaba Akram, Hubert Amu, Omid Dadras, Xiaochen Dai, Khalil Eskandari, Shilpa Gaidhane, Mahaveer Golechha, Mowafa Househ, Rabia Hussain, Pedram Keshavarz, Mahalaqua Nazli Khatib, Adnan Kisa, Sezer Kisa, Ali H Mokdad, Feng Sha, Anasthasia Zastrozhina, Christopher J L Murray, and Theo Vos.

Providing critical feedback on methods or results
Jaimie D Steinmetz, Stein Emil Vollset, Kai Fukutaki, Foad Abd-Allah, Ahmed Abualhasan, Tayyaba Akram, Hanadi Al Hamad, Fares Alahdab, Fahad Mashhour Alanezi, Vahid Alipour, Sami Almustanyir, Hubert Amu, Jalal Arabloo, Thomas Astell-Burt, Getinet Ayano, Jose L Ayuso-Mateos, Atif Amin Baig, Anthony Barnett, Amadou Barrow, Bernhard T Baune, Yannick Béjot, Woldesellassie M Mequanint Bezabhe, Yihienew Mequanint Bezabih, Akshaya Srikanth Bhagavathula, Sonu Bhaskar, Krittika Bhattacharyya, Ali Bijani, Atanu Biswas, Archith Boloor, Carol Brayne, Hermann Brenner, Katrin Burkart, Richard A Burns, Luis Alberto Cámera, Chao Cao, Luis F S Castro-de-Araujo, Ferrán Catalá-López, Ester Cerin, Prachi P Chavan, Nicolas Cherbuin, Dinh-Toi Chu, Vera Marisa Costa, Omid Dadras, Xiaochen Dai, Lalit Dandona, Rakhi Dandona, Vanessa De la Cruz-Góngora, Deepak

Dhamnetiya, Diana Dias da Silva, Daniel Diaz, Abdel Douiri, David Edvardsson, Michael Ekholuenetale, Iman El Sayed, Khalil Eskandari, Sharareh Eskandarieh, Saman Esmaeilnejad, Jawad Fares, Andre Faro, Umar Farooque, Valery L Feigin, Xiaoqi Feng, Seyed-Mohammad Fereshtehnejad, Pietro Ferrara, Irina Filip, Howard Fillit, Florian Fischer, Shilpa Gaidhane, Lucia Galluzzo, Ahmad Ghashghaee, Nermin Ghith, Alessandro Gialluisi, Elena V Gnedovskaya, Mahaveer Golechha, Rajeev Gupta, Veer Bala Gupta, Vivek Kumar Gupta, Mohammad Rifat Haider, Brian J Hall, Samer Hamidi, Asif Hanif, Graeme J Hankey, Shafiul Haque, Risky Kusuma Hartono, Ahmed I Hasaballah, Simon I Hay, Khezar Hayat, Mohamed I Hegazy, Golnaz Heidari, Reza Heidari-Soureshjani, Claudiu Herteliu, Mowafa Househ, Rabia Hussain, Bing-Fang Hwang, Licia Iacoviello, Olayinka Stephen Ilesanmi, Irena M Ilic, Milena D Ilic, Seyed Sina Naghibi Irvani, Masao Iwagami, Roxana Jabbarinejad, Louis Jacob, Vardhmaan Jain, Sathish Kumar Jayapal, Ranil Jayawardena, Ravi Prakash Jha, Jost B Jonas, Nitin Joseph, Rizwan Kalani, Himal Kandel, André Karch, Ayele Semachew Kasa, Gizat M Kassie, Pedram Keshavarz, Moien AB Khan, Mahalaqua Nazli Khatib, Tawfik Ahmed Muthafer Khoja, Jagdish Khubchandani, Min Seo Kim, Yun Jin Kim, Adnan Kisa, Sezer Kisa, Mika Kivimäki, Walter J Koroshetz, Ai Koyanagi, G Anil Kumar, Manasi Kumar, Hassan Mehmood Lak, Bingyu Li, Stephen S Lim, Xuefeng Liu, Yuewei Liu, Giancarlo Logroscino, Stefan Lorkowski, Giancarlo Lucchetti, Francesca Giulia Magnani, Ahmad Azam Malik, Ritesh G Menezes, Atte Meretoja, Yousef Mohammad, Arif Mohammed, Ali H Mokdad, Stefania Mondello, Mohammad Ali Moni, Md Moniruzzaman, Tilahun Belete Mossie, Gabriele Nagel, Sandhya Neupane Kandel, Trang Huyen Nguyen, Bogdan Oancea, Nikita Otstavnov, Stanislav S Otstavnov, Mayowa O Owolabi, Songhomitra Panda-Jonas, Fatemeh Pashazadeh Kan, Urvish K Patel, Mona Pathak, Mario F P Peres, Arokiasamy Perianayagam, Carrie B Peterson, Michael R Phillips, Michael A Piradov, Michele H Potashman, Faheem Hyder Pottoo, Sergio I Prada, Amir Radfar, Alberto Raggi, Fakher Rahim, Mosiur Rahman, Pradhum Ram, Priyanga Ranasinghe, David Laith Rawaf, Salman Rawaf, Nima Rezaei, Aziz Rezapour, Stephen R Robinson, Michele Romoli, Gholamreza Roshandel, Mohammad Ali Sahraian, Brijesh Sathian, Monika Sawhney, Mete Saylan, Allen Seylani, Feng Sha, Masood Ali Shaikh, KS Shaji, Mohammed Shannawaz, Jeevan K Shetty, Mika Shigematsu, Jae Il Shin, Rahman Shiri, Diego Augusto Santos Silva, João Pedro Silva, Renata Silva, Jasvinder A Singh, Valentin Yurievich Skryabin, Anna Aleksandrovna Skryabina, Amanda E Smith, Sergey Soshnikov, Jing Sun, Rafael Tabarés-Seisdedos, Bhaskar Thakur, Binod Timalsina, Marcos Roberto Tovani-Palone, Bach Xuan Tran, Gebiyaw Wudie Tsegaye, Sahel Valadan Tahbaz, Pascual R Valdez, Narayanaswamy Venketasubramanian, Giang Thu Vu, Linh Gia Vu, Anders Wimo, Andrea Sylvia Winkler, Lalit Yadav, Seyed Hossein Yahyazadeh Jabbari, Kazumasa Yamagishi, Lin Yang, Yuichiro Yano, Naohiro Yonemoto, Chuanhua Yu, Ismaeel Yunusa, Siddhesh Zadey, Mikhail Sergeevich Zastrozhin, Zhi-Jiang Zhang, Christopher J L Murray, and Theo Vos.

Drafting the work or revising is critically for important intellectual content

Emma Nichols, Stein Emil Vollset, Foad Abd-Allah, Ahmed Abualhasan, Eman Abu-Gharbieh, Tayyaba Akram, Fares Alahdab, Sami Almustanyir, Hubert Amu, Iman Ansari, Jalal Arabloo, Atif Amin Baig, Anthony Barnett, Amadou Barrow, Bernhard T Baune, Yannick Béjot, Yihienew Mequanint Bezabih, Akshaya Srikanth Bhagavathula, Sonu Bhaskar, Krittika Bhattacharyya, Atanu Biswas, Hermann Brenner, Chao Cao, Felix Carvalho, Luis F S Castro-de-Araujo, Ferrán Catalá-López, Ester Cerin, Nicolas Cherbuin, Dinh-Toi Chu, Vera Marisa Costa, Deepak Dhamnetiya, Diana Dias da Silva, Daniel Diaz, Abdel Douiri, David Edvardsson, Iman El Sayed, Shaimaa I El-Jaafary, Khalil Eskandari, Sharareh Eskandarieh, Saman Esmaeilnejad, Jawad Fares, Andre Faro, Umar Farooque, Valery L Feigin, Seyed-Mohammad Fereshtehnejad, Eduarda Fernandes, Pietro Ferrara, Irina Filip, Howard Fillit, Florian Fischer, Shilpa Gaidhane, Ahmad Ghashghaee, Nermin Ghith, Alessandro Gialluisi, Ionela-Roxana Glăvan, Elena V Gnedovskaya, Rajeev Gupta, Veer Bala Gupta, Vivek Kumar Gupta, Brian J Hall, Graeme J Hankey, Shafiul Haque, Ahmed I Hasaballah, M Tasdik Hasan, Amr Hassan, Simon I Hay, Golnaz Heidari, Claudiu Herteliu, Mowafa Househ, Rabia Hussain, Licia Iacoviello, Ivo Iavicoli, Olayinka Stephen Ilesanmi, Irena M Ilic, Milena D Ilic, Seyed Sina Naghibi Irvani, Hiroyasu Iso, Louis Jacob, Vardhmaan Jain, Sathish Kumar Jayapal, Ranil Jayawardena, Ravi Prakash Jha, Jost B Jonas, Nitin Joseph, Rizwan Kalani, Amit Kandel, Himal Kandel, André Karch, Ayele Semachew Kasa, Gizat M Kassie, Pedram Keshavarz, Moien AB Khan, Mahalaqua Nazli Khatib, Jagdish Khubchandani, Yun Jin Kim, Adnan Kisa, Sezer Kisa, Mika Kivimäki, Walter J Koroshetz, Ai Koyanagi, Hassan Mehmood Lak, Matilde Leonardi, Stefan Lorkowski, Giancarlo Lucchetti, Ricardo Lutzky Saute, Francesca Giulia Magnani, Ahmad Azam Malik, João Massano, Man Mohan Mehndiratta, Ritesh G Menezes, Atte Meretoja, Bahram Mohajer, Yousef Mohammad, Arif Mohammed, Ali H Mokdad, Stefania Mondello, Mohammad

Ali Moni, Md Moniruzzaman, Tilahun Belete Mossie, Muhammad Naveed, Vinod C Nayak, Sandhya Neupane Kandel, Trang Huyen Nguyen, Bogdan Oancea, Nikita Otstavnov, Stanislav S Otstavnov, Mayowa O Owolabi, Songhomitra Panda-Jonas, Fatemeh Pashazadeh Kan, Maja Pasovic, Urvish K Patel, Mona Pathak, Mario F P Peres, Arokiasamy Perianayagam, Michael R Phillips, Michael A Piradov, Constance Dimity Pond, Sergio I Prada, Amir Radfar, Alberto Raggi, Fakher Rahim, Pradhum Ram, Priyanga Ranasinghe, David Laith Rawaf, Salman Rawaf, Nima Rezaei, Stephen R Robinson, Gholamreza Roshandel, Ramesh Sahathevan, Amirhossein Sahebkar, Mohammad Ali Sahraian, Davide Sattin, Mete Saylan, Silvia Schiavolin, Allen Seylani, KS Shaji, Mohammed Shannawaz, Jeevan K Shetty, Mika Shigematsu, Diego Augusto Santos Silva, João Pedro Silva, Jasvinder A Singh, Valentin Yurievich Skryabin, Anna Aleksandrovna Skryabina, Sergey Soshnikov, Dan J Stein, Bhaskar Thakur, Marcos Roberto Tovani-Palone, Bach Xuan Tran, Sahel Valadan Tahbaz, Narayanaswamy Venketasubramanian, Vasily Vlassov, Giang Thu Vu, Linh Gia Vu, Yuan-Pang Wang, Anders Wimo, Andrea Sylvia Winkler, Lalit Yadav, Seyed Hossein Yahyazadeh Jabbari, Kazumasa Yamagishi, Lin Yang, Naohiro Yonemoto, Mikhail Sergeevich Zastrozhin, Anasthasia Zastrozhina, Christopher J L Murray, and Theo Vos.

Extracting, cleaning, or cataloging data; designing or coding figures and tables

Emma Nichols, Jaimie D Steinmetz, Tayyaba Akram, Hubert Amu, Khalil Eskandari, Saman Esmaeilnejad, Shilpa Gaidhane, Ahmed I Hasaballah, Mowafa Househ, Rabia Hussain, Mahalaqua Nazli Khatib, Adnan Kisa, Sezer Kisa, Ali H Mokdad, and Emma Elizabeth Spurlock.

Managing the overall research enterprise

Stein Emil Vollset, Lalit Dandona, Simon I Hay, Ali H Mokdad, Amanda E Smith, Christopher J L Murray, and Theo Vos.

Dementia prevalence flowchart

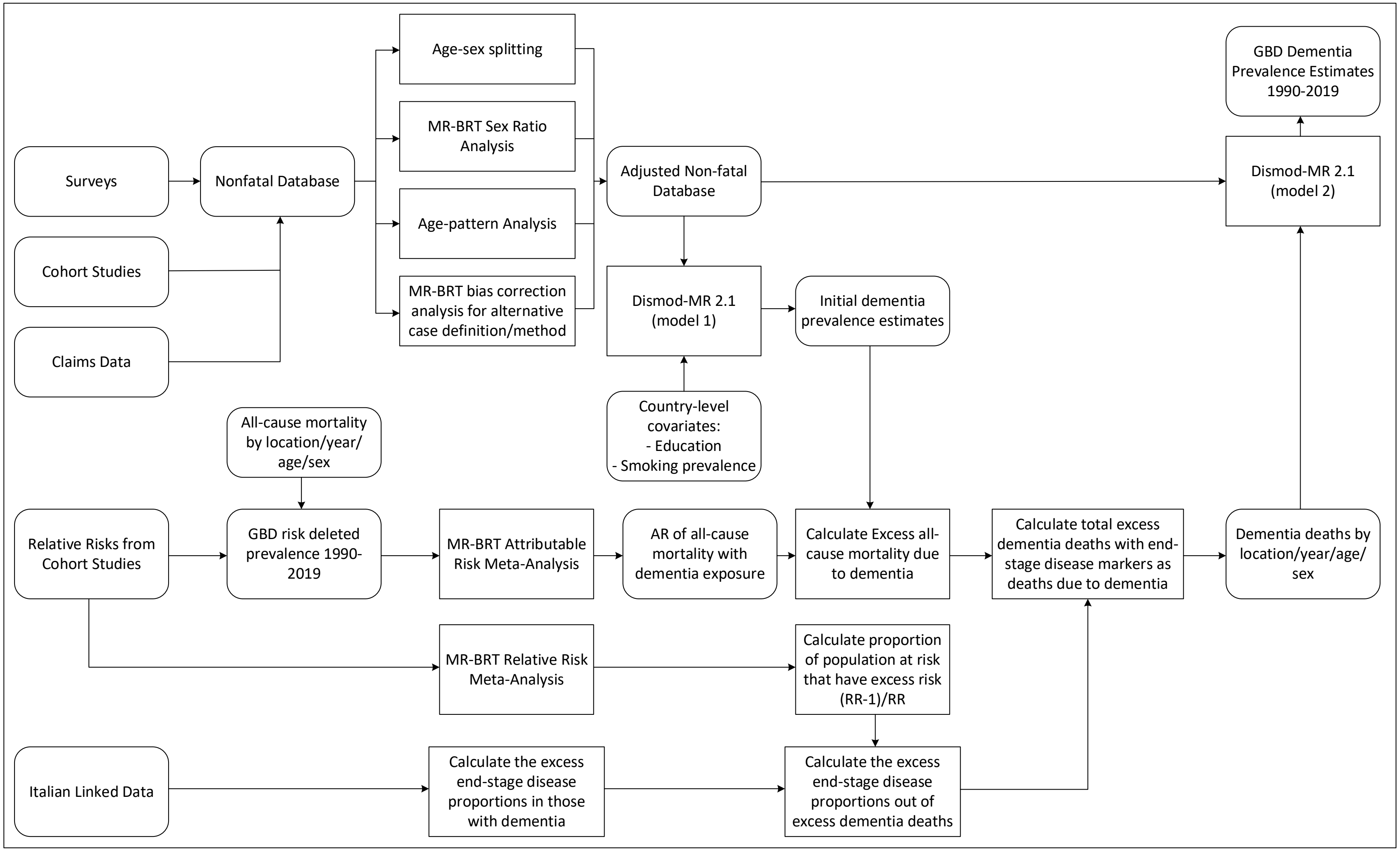

Additional details on the estimation of dementia prevalence and mortality can be found in the Appendix section on the Methods for the Estimation of Dementia. Further discussion of the estimation of dementia mortality can also be found in "GBD 2019 Collaborators. Global mortality from dementia: Application of a new method and results from the Global Burden of Disease Study 2019. Alzheimers Dement (N Y). 2021 Jul 27;7(1):e12200. doi: 10.1002/trc2.12200. PMID: 34337138; PMCID: PMC8315276."

Dementia forecasting flowchart

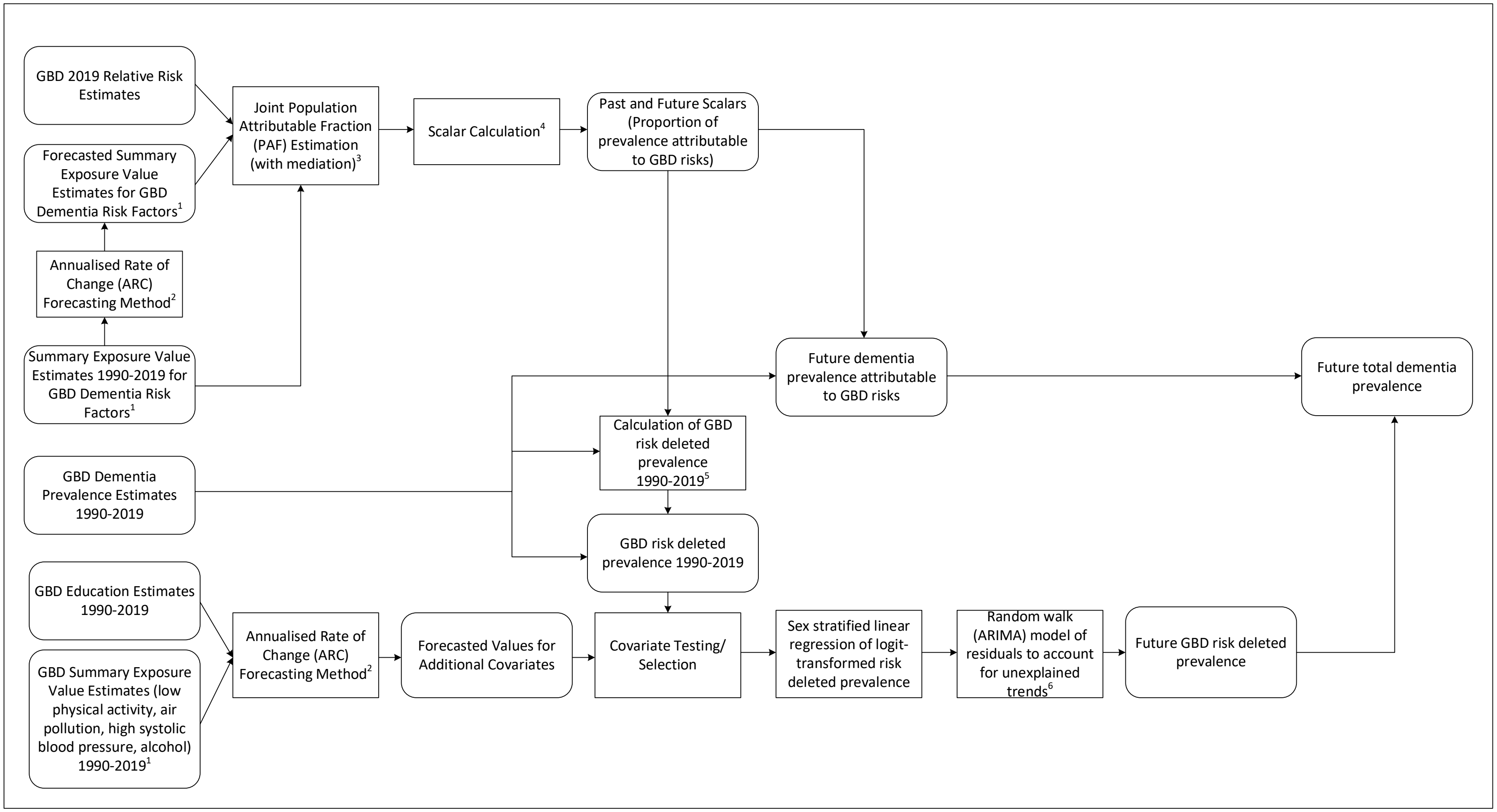

1. Summary exposure values (SEVs) are a relative risk-weighted prevalence of a given risk factor exposure. For binary risk factors the SEV is equal to risk factor prevalence, but for continuous risks the SEV upweights prevalence where the relative risk is highest. The SEV is equal to zero when there is no excess risk in a population and equal to one when the population risk is highest.
2. The annualised rate of change forecasting method works by using prior information on annualised rate of change to predict trends into the future. Out-of-sample predictive validity is used to decide how much weight to give to more recent trends versus older trends.
3. The population attributable fraction (PAF) represents the proportion of disease prevalence in a population that is attributable to a set of risk factors. The population attributable fractions for different risk factors cannot be simply added together because the effect of some risks may be mediated through others. We estimated mediation factors using information on crude and adjusted risks either from individual-level or from the literature.
4. Scalars represent the proportion of dementia prevalence that is attributable to GBD risk factors, and is calculated as 1/(1-PAF).
5. To calculate risk deleted prevalence, you would divide the prevalence of dementia by the scalar.
6. A random walk is a time series model that assumes that each time period takes one additional step away from it's previous value, and that all steps are independent from prior steps. This component of the model accounts for unexplained trends and helps to accurately capture uncertainty in the forecasts.

General methods for the Global Burden of Disease (GBD) study

(Adapted from "Global, regional, and national burden of Alzheimer's disease and other dementias, 1990–2016: a systematic analysis for the Global Burden of Disease Study 2016" by GBD 2016 Dementia Collaborators, *The Lancet* 2019; 18: 88-106.)

The Institute for Health Metrics and Evaluation, with a growing collaboration of scientists, produces annual updates of the Global Burden of Disease study. Estimates span the period from 1990 to the most recent completed year. As of June 2021, there were over 7,041 collaborators in 156 countries who contributed to this global public good. Annual updates allow incorporation of new data and method improvements to ensure that the most up-to-date information is available to policy makers in a timely fashion to help make resource allocation decisions. The guiding principle of GBD is to assess health loss due to mortality and disability comprehensively, where we define disability as any departure from full health. In GBD 2019, estimates were made for 204 countries and territories, and 808 subnational locations, for 29 years starting from 1990, for 23 age groups and both sexes. Deaths were estimated for 276 diseases and injuries, while prevalence and incidence were estimated for 354 diseases and injuries.

Non-fatal estimates are based on systematic reviews of published papers and unpublished documents, survey microdata, administrative records of health encounters, registries, and disease surveillance systems. Our Global Health Data Exchange (GHDx, http://ghdx.healthdata.org/) is the largest repository of health data globally. We first set a reference case definition and/or study method that best quantifies each disease or injury or consequence thereof. If there is evidence of a systematic bias in data that used different case definitions or methods compared to reference data, we adjust those data points to reflect what its value would have been if measured as the reference. This is a necessary step if one wants to use all data pertaining to a particular quantity of interest rather than choosing a small subset of data of the highest quality only. DisMod-MR 2.1, a Bayesian meta-regression tool, is our main method of analysing non-fatal data. It is designed as a geographical cascade where a first model is run on all the world's data, which produces an initial global fit and estimates coefficients for predictor variables and the adjustments for alternative study characteristics. The global fit, adjusted by the values of random effects for each of seven GBD super-regions, the coefficients on sex and country predictors, are passed down as data to a model for each super-region together with the input data for that geography. The same steps are repeated going from super-region to 21 region fits and then to 195 fits by country and, where applicable, a further level down to subnational units. Below the global fit, all models are run separately by sex and for eight time periods: 1990, 1995, 2000, 2005, 2010, 2015, 2017, and 2019. During each fit all data on prevalence, incidence, remission (ie, cure rate) and mortality are forced to be internally consistent.

For risks, we use a "counterfactual" approach, ie, answering the question: "what would the burden have been if the population had been exposed to a theoretical minimum level of exposure to a risk?" Thus, we need to define what level of exposure to a risk factor leads to the lowest amount of disease. We then analyse data on the prevalence of exposure to a risk and derive relative risks for any risk-outcome pair for which we find sufficient evidence of a causal relationship. Prevalence of exposure is estimated in DisMod-MR 2.1, using spatiotemporal Gaussian process regression, or from satellite imagery in the case of ambient air pollution. Relative risk data are pooled using meta-analysis of cohort, case-control, and/or intervention studies.

From the prevalence and relative risk results, population attributable fractions are estimated relative to the theoretical minimum risk exposure level (TMREL). When we aggregate estimates for clusters of risks, eg, metabolic or behavioural risks, we use a multiplicative function rather than simple addition and take into account how much of each risk is mediated through another risk. For instance, some of the risk of high body-mass index is directly onto stroke as an outcome, but much of its impact is mediated through high blood pressure, high cholesterol, or high fasting plasma glucose, and we would not want to double count the mediated effects when we estimate aggregates across risk factors.

Uncertainty is propagated throughout all these calculations by creating 1,000 values for each prevalence, death, YLL, YLD, or DALY estimate and performing aggregations across causes and locations at the level of each of the 1,000 values for all intermediate steps in the calculation. The lower and upper bounds of the 95% uncertainty interval are the 25th and 975th values of the ordered 1,000 values. For all age-standardised rates, GBD uses a

standard population calculated as the non-weighted average across all countries of the percentage of the population in each five-year age group for the years 2010 to 2035. GBD uses a composite indicator of sociodemographic development, the Socio-demographic Index (SDI), which reflects the geometric mean of normalised values of a location's income per capita, the average years of schooling in the population 15 and over, and the total fertility rate. Countries and territories are grouped into five quintiles of high, high-middle, middle, low-middle, and low SDI based on their 2019 values.

Methods for the estimation of dementia

(Adapted from Global burden of 369 diseases, injuries, and impairments, 1990–2019: a systematic analysis for the Global Burden of Disease Study 2019" by GBD 2019 Diseases, Injuries, and Impairments Collaborators, *The Lancet* 2020; 396: 1204–22.)

Case definition

Dementia is a progressive, degenerative, and chronic neurological disorder typified by memory impairment and other neurological dysfunctions. For the purposes of GBD 2019, we use the Diagnostic and Statistical Manual of Mental Disorders III, IV, or V, or ICD case definitions as the reference. The DSM-IV definition is:

- Multiple cognitive deficits manifested by both memory impairment and one of the following: aphasia, apraxia, agnosia, disturbance in executive functioning
- Must cause significant impairment in occupational functioning and represent a significant decline
- Course is characterised by gradual onset and continuing cognitive decline
- Cognitive deficits are not due to other psychiatric conditions
- Deficits do not occur exclusively during the course of a delirium

A wide array of diagnostic and screening instruments exists, including Clinical Dementia Rating scale (CDR), Mini Mental State Examination (MMSE), and the Geriatric Mental State (GMS). For severity rating purposes we use the CDR as the reference. The relevant ICD-10 codes for dementia are F00, F01, F02, F03, G30, and G31. The ICD-9 codes are 290, 291.2, 291.8, 294, and 331.

Unlike most causes in the Global Burden of Disease project, dementia mortality and morbidity estimates are modelled jointly. This is because of marked discrepancies between prevalence data and cause of death data. Specifically, prevalence data suggest little to no variation over time (eg, 1990–2019), whereas age-standardised mortality rates in vital registrations in high-income countries have increased multiple times over this same period. Additionally, prevalence variation between countries is much smaller than the variation in death rates assigned to dementia in vital registration. We attribute these discrepancies to changing coding practices rather than epidemiological change.

Input data for prevalence estimation

Model inputs

To inform our estimates of burden due to dementia, we use mortality data from vital registration systems, as well as prevalence data from surveys and administrative data such as claims sources.

Item response theory for prevalence prediction

The prevalence models for dementia are data-sparse, and there aren't many surveys done in low-income settings. However, there is a larger body of surveys that collect data on cognitive tests and functional limitations, which are the two main components of a DSM or ICD diagnosis. Predictions of dementia prevalence using information from these questions would allow for expanded data coverage and additional information in locations where there are currently no data guiding estimates.

Generating these predictions requires calibrating a model to samples that have information about both functional limitations, cognition, and adjudicated dementia diagnoses. However, making comparisons across surveys can be difficult, as each survey asks a different set of questions about cognition and limitations, although there is some overlap. This overlap allows for the use of item-response theory methods for the harmonisation of these scales. Once the scales are harmonised, the subsamples can be utilised to create a model for the prediction of prevalence.

In GBD 2019, data from the ADAMS and HRS surveys were extracted and used for item-response theory modelling to estimate prevalence. HRS is a nationally representative survey in the USA, which has data on cognition and functional limitations. ADAMS is a subsample of HRS that includes much more detailed neuropsychological testing and adjudicated dementia diagnoses. ADAMS includes almost all questions in HRS plus additional questions.

Excluding incidence

Since 2016, we have made the decision to exclude incidence data, because in locations with high-quality cohort data on prevalence and incidence, the two are not compatible (incidence data imply a higher prevalence than what is reported). Because dementia has a slow, insidious onset and prevalence is easier to measure, we trust prevalence data more and rely on this, excluding incidence data from DisMod.

Modelling strategy for prevalence estimation

First, prevalence data was sex-split, crosswalked, and age-split. Studies with age and sex detail separately were split into age- and sex-specific datapoints. Data specified as "both"-sex data were split into male- and female-specific datapoints using MR-BRT to get a model ratio of female/male prevalence and then using the following equations:

Male prevalence:

$$prev_{male} = prev_{both} * \frac{pop_{both}}{(pop_{male} + ratio * pop_{female})}$$

Female prevalence:

$$prev_{female} = ratio * prev_{male}$$

We also split datapoints where the age range was greater than 25 years using the global age pattern.

Dementia studies are heterogeneous. Even with a smaller number of definitions (DSM/ICD), there are a large number of different ways to diagnose dementia. For example, out of 272 sources used in GBD 2017, there were 263 different methods of diagnosing dementia (overlap was among those who used 10/66 protocol or AGECAT algorithm). Most use a two-step procedure, where clinicians screen patients using a cognitive test and then only fully evaluate those who fall below a certain pre-defined threshold. We controlled for methods differences by crosswalking alternative case definitions to reference. Study covariates are based on broad categories determined after going through the diagnostic heterogeneity, and there are some added for specific criteria that we know are biased. The same study-level covariates were used in 2019 as in 2017, with the addition of item-response theory HRS predictions. Crosswalking was carried out using a logit difference network meta-regression analysis. US MarketScan data were separately crosswalked to standardise the claims data relative to existing literature data.

MR-BRT crosswalk adjustment factors for dementia (network analysis)

Data input	Reference or alternative case definition	Gamma	Beta Coefficient, logit (95% CI)
DSM or ICD case definition	Ref	0.34	---
Clinical records diagnosis criteria	Alt		–0.05 (–0.72 to 0.61)
Algorithm diagnosis criteria (AGECAT)	Alt		0.08 (–0.59 to 0.74)
US MarketScan	Alt		–0.95 (–1.61 to –0.28)
NIA-AA diagnosis criteria	Alt		0.51 (–0.16 to 1.17)

10/66 algorithm diagnosis criteria	Alt		0.97 (0.30 to 1.64)
GP records used for diagnosis	Alt		–1.21 (–1.88 to –0.54)

A separate analysis was conducted to crosswalk MarketScan claims data (excluding MarketScan year 2000) to non-claims data using a spline on age. The plot below shows the model fit over different ages (gamma = 0.07).

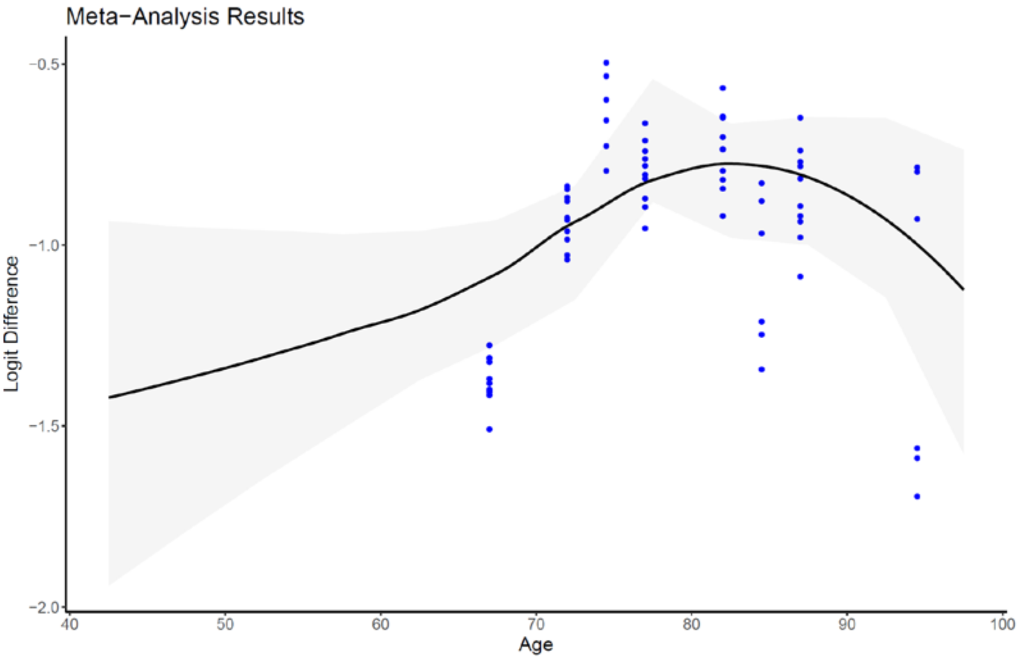

Two country-level covariates were included in the initial DisMod model. Age-standardised education was used as a proxy for general brain health/use that may be protective for dementia. We set priors on the covariate value for education based on the results of a review of the literature for the effect of each additional year of education on dementia prevalence.

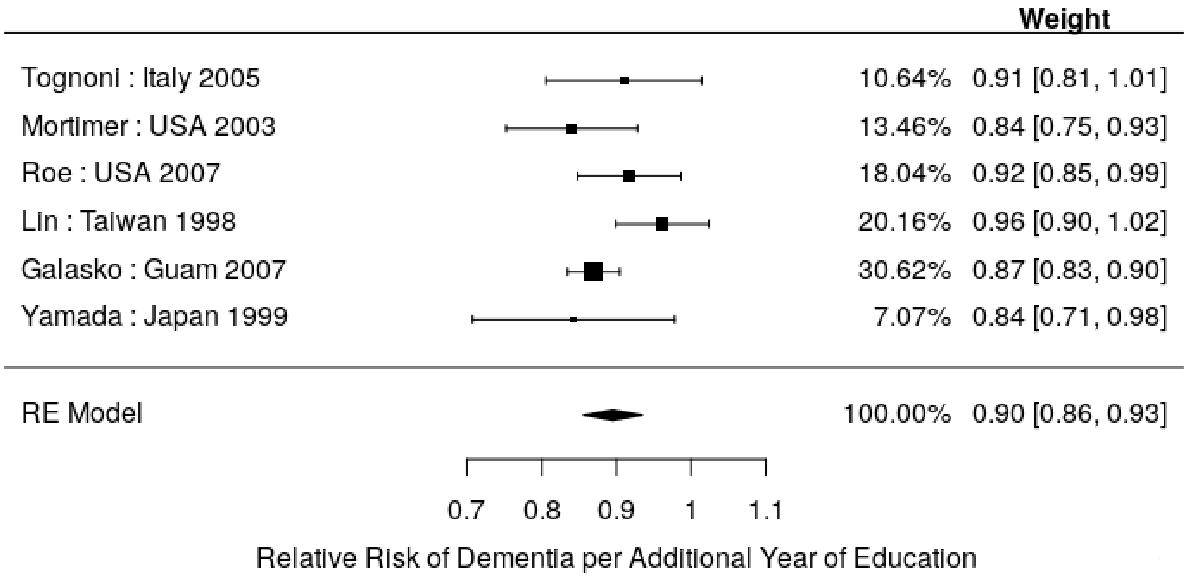

Smoking prevalence (age-standardised, both sexes) was also used as a covariate to guide estimates, as the literature has shown a positive relationship between smoking and dementia.

Note that two DisMod models were run with prevalence inputs – the first uses adjusted prevalence data (DisMod Model 1 in flowchart), which accounts for dementia caused by other diseases. The second uses unadjusted dementia (DisMod Model 2 in flowchart), which accounts for all dementia regardless of cause (this is the dementia impairment envelope). The tables below summarise country-level covariates used in each of these DisMod model.

Covariates. Summary of covariates used in the dementia DisMod-MR meta-regression model (adjusted prevalence, Model 1)

**Covariate**	**Type**	**Exponentiated beta (95% uncertainty interval)**
Smoking prevalence (age-standardised)	Prevalence	2.71 (1.03–7.36)
Years of Education (age-standardised)	Prevalence	0.92 (0.92–0.92)

Covariates. Summary of covariates used in the Dementia DisMod-MR meta-regression model (unadjusted prevalence, Model 2)

**Covariate**	**Type**	**Exponentiated beta (95% uncertainty interval)**

Smoking prevalence (age-standardised)	Prevalence	1.00 (1.00–1.01)
Years of Education (age-standardised)	Prevalence	0.92 (0.92–0.92)

Input data for mortality estimation

In GBD 2019, fatal modelling was redesigned to remove reliance on vital registration data (described in more detail in the "Modelling strategy" section). Instead, two new source types were extracted:

(1) Literature on the relative risk of all-cause mortality given the exposure of dementia. Relative risk sources were identified through a systematic review using search terms[2] in PubMed. This yielded 4470 total hits, of which 34 studies were marked for extraction. Overall, the data were heterogeneous and varied in the exposure category measured (all dementia, Alzheimer's disease, cognitive impairment) and in the different factors controlled for in analyses.

(2) Linked vital registration and hospitalisation data. We used mortality records linked to inpatient records, covering all deaths from 2003 to 2017 in the Emilia-Romagna region of Italy.

Table 1: Results of systematic review on all-cause excess mortality with dementia

N		60
Region name (%)	East Asia	4 (6.7)
	Eastern sub-Saharan Africa	2 (3.3)
	High-income Asia Pacific	4 (6.7)
	High-income North America	22 (36.7)
	North Africa and Middle East	1 (1.7)
	Tropical Latin America	1 (1.7)
	Western Europe	26 (43.3)
Exposure (%)	Alzheimer's disease	11 (18.3)
	cognitive impairment	10 (16.7)
	other dementia	35 (58.3)
	vascular dementia	4 (6.7)
Conducted in clinical setting (%)	Clinical setting	10 (16.7)
	Population representative	50 (83.3)
Controlled for education (%)	Controlled	32 (53.3)
	No control	28 (46.7)
Controlled for basic CVD info (%)	Controlled	33 (55.0)
	No control	27 (45.0)
Extensive CVD control (%)	Controlled	15 (25.0)
	No control	45 (75.0)
Controlled for smoking and alcohol (%)	Controlled	11 (18.3)
	No control	49 (81.7)
Controlled for factors in causal pathway (%)	Controlled	13 (21.7)
	No control	47 (78.3)

Modelling strategy for mortality estimation

Dementia mortality rates have increased more than five-fold since 1980 in high-quality vital registration systems such as in the USA and Scandinavia. We have not seen an equivalent increase in prevalence and incidence data sources. If at all, there has been a modest decline in incidence and prevalence of dementia in studies in the UK and the USA.[1,2] Also, the greater than 20-fold variation in mortality rates of dementia between countries is much greater than the four-fold difference in prevalence and incidence between countries. As it is unlikely that case fatality from dementia has dramatically increased over the time period and that it would differ by a very large margin between countries, the hypothesis is that certifying and coding practices have changed over time and at a different pace between countries. To avoid spurious large trends over time in the fatal component of the burden of dementia, we decided for GBD 2013 to make dementia mortality rates consistent with the most recent rates relative to prevalence of countries that are most likely to certify or code dementia as an underlying cause of death. This approach was applied again for GBD 2017 with some modifications. For GBD 2019, the fatal modelling process was redesigned to avoid the need for using estimates only from locations with the highest dementia mortality. This was accomplished with an attributable risk model based on a systematic review of cohort studies and relative risk data, and end-stage disease proportions from linked hospital and death records. The modelling process is described below.

Modelling steps

Relative risk data

First, using relative risk data extracted from studies identified by systematic review, we calculated attributable risk and the GBD estimate of all-cause mortality rate for a given study location and time, using the following formula:

$$Attributable\ Risk = (Relative\ Risk - 1) * All\text{-}Cause\ Mortality$$

We then conducted a meta-analysis on the attributable risk data, using covariates for age, sex, exposure category (all dementia, Alzheimer's disease, cognitive impairment), whether the study was conducted in a clinical sample, and categories indicating different types of variables that were controlled for in the component studies (educational attainment, cardiovascular disease comorbidities, smoking and alcohol consumption, and daily activities or residence in a nursing home). Relative risks were estimated using a second Bayesian bias-reduction meta-regression model and the same studies identified through systematic review. Regression results for relative risk and attributable risk analyses are displayed below.

---

[1] Akushevich I, Kravchenko J, Ukraintseva S, Arbeev K, Yashin AI. Time trends of incidence of age-associated diseases in the US elderly population: Medicare-based analysis. *Age and ageing*. 2013 Jul 1;42(4):494-500.

[2] Matthews FE, Arthur A, Barnes LE, Bond J, Jagger C, Robinson L, Brayne C, Medical Research Council Cognitive Function and Ageing Collaboration. A two-decade comparison of prevalence of dementia in individuals aged 65 years and older from three geographical areas of England: results of the Cognitive Function and Ageing Study I and II. *The Lancet*. 2013 Nov 1;382(9902):1405-12.

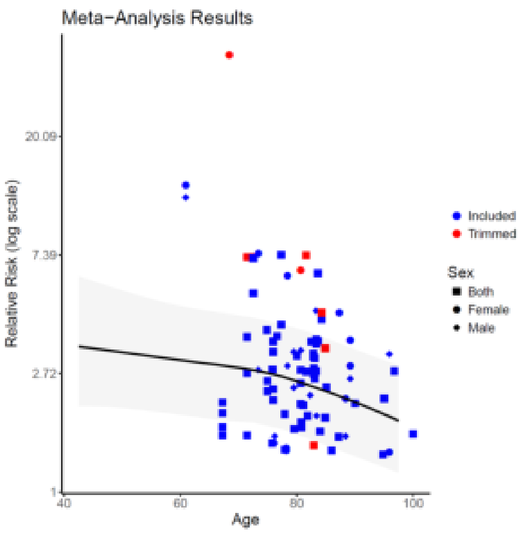

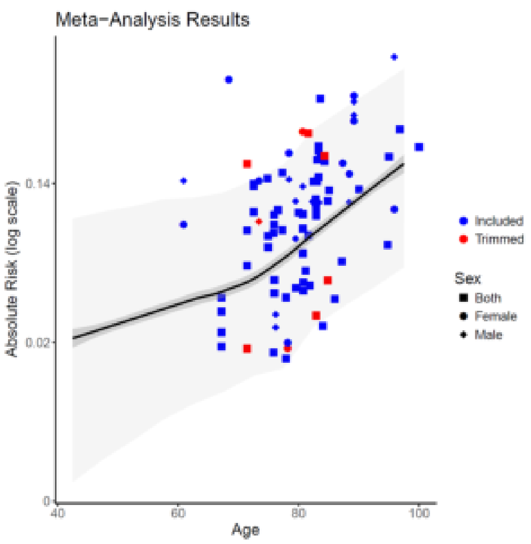

Meta-regression results were used to calculate the total number of excess deaths due to dementia as the product of our prevalence estimates (post-adjustment for dementia caused by other GBD diseases) and our estimates of attributable risk.

Linked data

The excess deaths calculated through the multiplication of attributable risk and prevalence represent the total number of excess deaths due to having dementia, which likely includes deaths due to other conditions, such as cardiovascular diseases, that are more common in those with dementia as compared to the general population due to common underlying risk factors such as blood pressure, smoking, and lower educational attainment. In order to subset this total number of excess dementia deaths to calculate the number of deaths that were caused by dementia, we completed an analysis of linked clinical and mortality data. We used mortality records linked to inpatient records, covering all deaths from 2003 to 2017 in the Emilia-Romagna region of Italy. Using these data, we looked for markers of severe, end-stage disease in the clinical records up to one year before death.

To select these markers, for each ICD code that appeared in the data we calculated the difference in the proportion of individuals who died with dementia and had a record of each code in the year before death and the proportion of individuals who died without dementia and had a record of the same code in the year before death. We reviewed the 150 codes with the highest difference and selected codes that indicated end-stage disease, excluding codes for conditions such as cardiovascular disease. Codes for decubitus ulcer, malnutrition, sepsis, pneumonia, urinary tract infections, falling from bed, senility, dehydration, sodium imbalance, muscular wasting, bronchitis, dysphagia, hip fracture, and bedridden status were used as indicators of severe disease.

In order to determine the proportion of excess deaths that were caused by dementia, we calculated the proportion of dementia deaths that had clinical markers of end-stage disease in the year before death, above and beyond the occurrence of end-stage disease markers in those who died without dementia. The subtraction of the proportions with end-stage disease markers in those without dementia from the proportions in those with dementia represents the proportion of individuals who are assumed to have died with severe, end-stage dementia out of total deaths in those with dementia.

Figure 2: Percent of individuals who died with and without dementia with end stage conditions in the last year before death

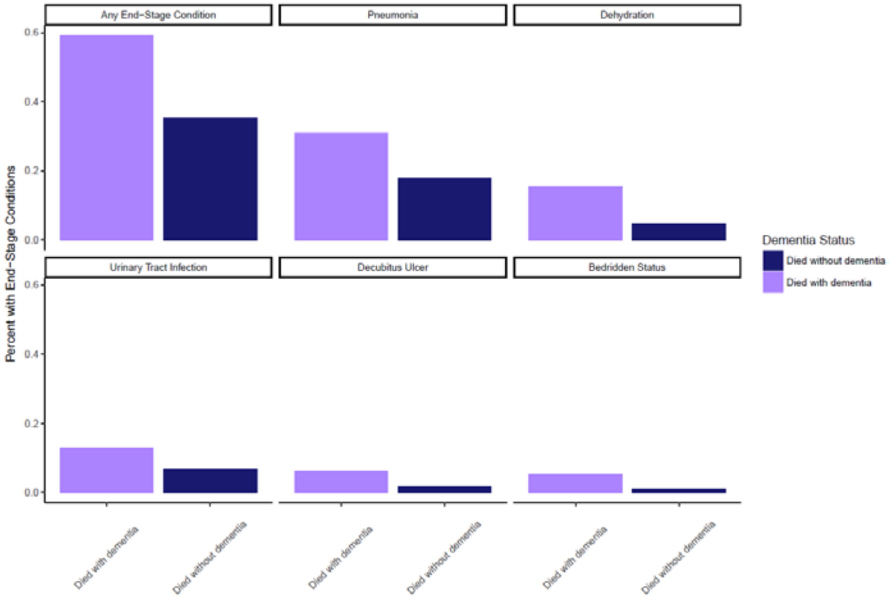

Calculation of deaths due to dementia

In order to apply these estimates to the total excess deaths we then adjusted these proportions to calculate the proportion of individuals who died with severe, end-stage dementia out of excess dementia deaths using the formula:

$$\frac{Died\ with\ Severe\ Disease}{Excess\ Dementia\ Deaths} = \frac{Died\ with\ Severe\ Disease}{Total\ Dementia\ Deaths} * \frac{Relative\ Risk}{Relative\ Risk - 1}$$

We then calculated the number of deaths due to dementia as the product of total excess dementia deaths and the proportion of those who died with severe disease out of excess dementia deaths. These final estimates of deaths due to dementia were then used to adjust data on causes of death from all other causes in vital registration systems.

Interpolation for all years

Finally, we used log-linear interpolation to interpolate these results (limited to 1990, 1995, 2000, 2005, 2010, 2015, 2017, 2019) to create estimates for the entire time series from 1980 to 2019. Socio-demographic Index was used as a covariate to extrapolate back to the year 1980.

Methods for the calculation of SEV scalars

(Adapted from "Forecasting life expectancy, years of life lost, and all-cause and cause-specific mortality for 250 causes of death: reference and alternative scenarios for 2016–40 for 195 countries and territories" by Foreman et al., The Lancet 2018; 392: 2052–90.)

The GBD 2015 introduced summary exposure values (SEVs), a univariate measure of risk-weighted exposure. The SEV is the relative risk-weighted prevalence of exposure, where 0 is no risk in population and 1 is the entire population at maximum risk.

$$SEV_{rc} = \frac{\int_l^L p_l \, RR_l dl - RR_{min}}{(RR_{max} - 1)RR_{min}}$$

where l denotes a category of exposure, as in low, medium, or high. If we set $RR_{min} = 1$

$$SEV_{rc} = \frac{\int_l^L p_l \, RR_l dl - 1}{RR_{max} - 1}$$

Where RR_{max} is the relative risk at the highest level of exposure in theory or observed globally.

Stated in terms of a population attributable fraction (PAF), the SEV is

$$SEV_{rc} = \frac{PAF_{rc}}{(1 - PAF_{rc})(RR_{max} - 1)}$$

In order to only have one SEV per risk factor, we averaged it across the causes which are affected by each risk factor:

$$SEV_r = \frac{1}{N(c)} \sum_c SEV_{rc}$$

Methods for the calculation of population attributable fractions (PAFs), PAF mediation, and risk factor scalars

(Adapted from "Forecasting life expectancy, years of life lost, and all-cause and cause-specific mortality for 250 causes of death: reference and alternative scenarios for 2016–40 for 195 countries and territories" by Foreman et al., The Lancet 2018; 392: 2052–90.)

We generated estimated risk-specific PAFs for the future by converting the forecasted SEV values to PAF as follows:

$$\widehat{PAF_r} = 1 - \frac{1}{(SEV_r * (RR_r^{max} - 1) + 1)}.$$

These PAFs are also location-, age-, and year-specific due to the use of location-, age-, and year-specific SEV estimates.

PAF estimates depend on SEVs, which are not cause-specific; therefore, we expect a bias in logit-transformed space, which is the space where exposures are modelled. We try to correct for this bias by forcing our estimated values to agree with the GBD in the year 2016. This is done by first taking a reference PAF directly computed from exposure and cause-specific relative risks available in the GBD:

$$PAF_r = \frac{\sum_x^X p_{rx} * RR_{xr} - 1}{\sum_x^X p_{rx} * RR_{xr}}$$

where x corresponds to the different exposure levels of the risk factor. This is followed by calculating the correction factor CF via comparing (in logit space) the GBD PAF to the SEV-derived estimated PAF in the reference year 2019:

$$CF_r = logit(PAF_{r2019}) - logit(\widehat{PAF_{r2019}})$$

This correction factor is necessary because the SEV is summarised across all of the causes of death related to that risk factor. If there are different patterns of relative risk by exposure level for different causes of death for the same risk factor, there is some information loss attributable to this dimensionality reduction. Since that correction factor is relatively stable over time, we can simply add it to each year in the forecast to approximate the cause-risk-specific PAF accounting for these different relative risk curves.

We applied the correction factor to the estimated PAF to come up with an adjusted estimated PAF*:

$$PAF_r^* = expit(logit(\widehat{PAF_r}) + CF_r)$$

To properly estimate the joint PAF of all risks, one must take into account of how one risk factor is mediated through other risk factors. The fraction of one risk that is mediated through another is called mediation factor (MF). Using risk mediation factors provided in GBD 2019, we computed the joint (adjusted) PAF of all risks for a cause:

$$PAF = 1 - \prod_r^R (1 - PAF_r^* * \prod_s^S (1 - MF_{risk}))$$

Where s denotes the risks that impact the dementia via r, and r are all the GBD risk factors associated with dementia. Since PAF is the ratio of risk-attributable cause-specific deaths to total cause-specific deaths, we can relate total dementia prevalence to dementia prevalence attributable to GBD risk factors:

$$PAF = \frac{prevalence\ attributable\ to\ GBD\ risks}{total\ prevalence}$$

&

$$Total\ prevalence = prevalence\ attributable\ to\ GBD\ risks + prevalence\ not\ attributable\ to\ GBD\ risks$$

$$\therefore$$

$$Total\ prevalence = prevalence\ not\ attributable\ to\ GBD\ risks * \frac{1}{1 - PAF}$$

Finally, we generated a risk factor scalar, corresponding to the ratio of total cause-specific mortality to underlying cause-specific mortality.

$$Scalar = \frac{1}{1 - PAF}$$

Selection of risk factors for final forecasting model

After accounting for the three risk factors included for dementia in the GBD framework (high BMI, high fasting plasma glucose, and smoking), we evaluated five other covariates to assess their relationship with dementia prevalence not attributable to the GBD risk factors. The five covariates tested represent other covariates estimated within the GBD project and for which the 2020 *Lancet* Commission report reported evidence of a link with dementia: low physical activity, high systolic blood pressure, education, air pollution, and alcohol use. We ran sex-stratified linear regression models on logit prevalence with each risk factor individually as well as dummy variables for five-year age groups. The effect estimates (standardised to represent the effect per standard deviation unit of the risk factor) are shown below.

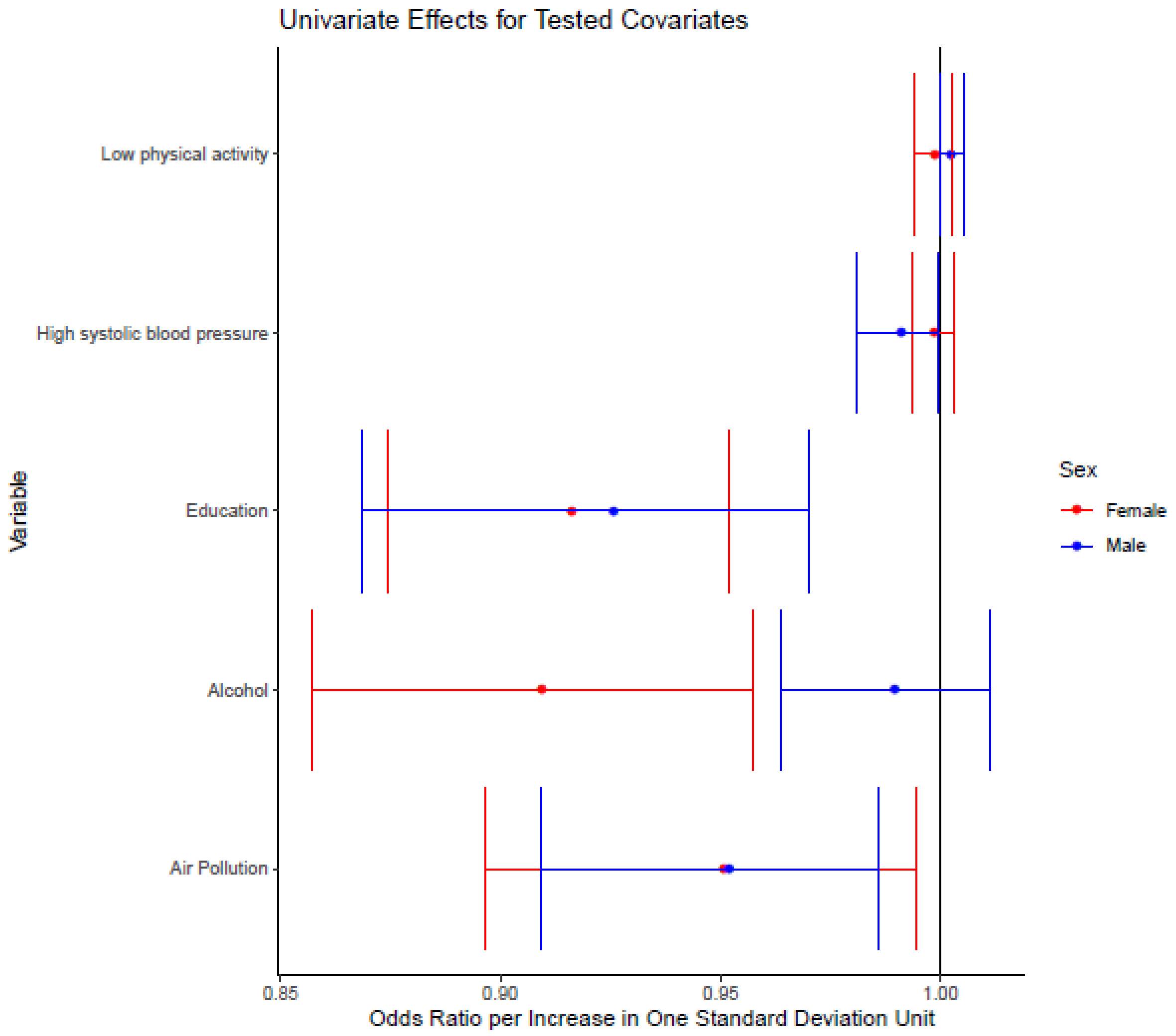

We retained covariates for which the direction of the effect estimate was statistically significant and consistent with that reported in the 2020 *Lancet* Commission report for both males and females. This led us to retain education in our final models.

Effects of age group and risk factors in full forecasting model

The plots below show the effects of the covariates in the final forecasting model.

Effects for age group (reference group: 40–44 years old)

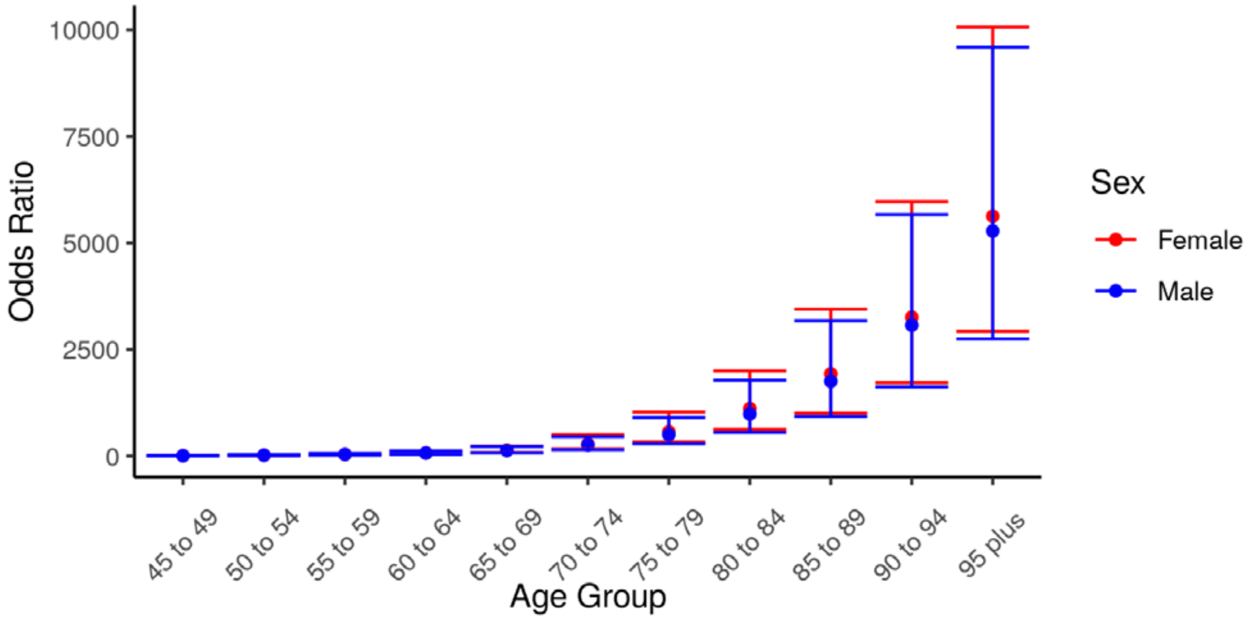

Effects for covariates for the prediction of dementia not attributable to GBD risk factors (per standard deviation increase in risk)

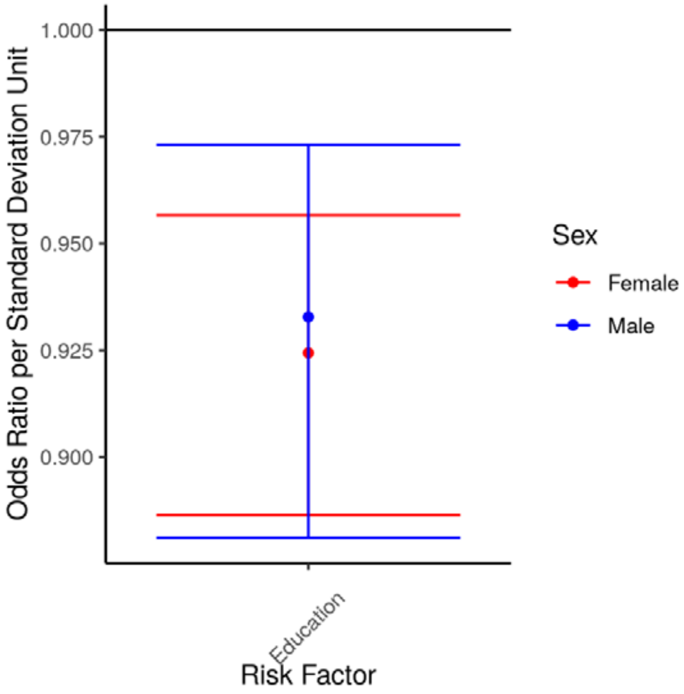

Air pollution estimation methods

(Adapted from “Global burden of 87 risk factors in 204 countries and territories, 1990-2019: a systematic analysis for the Global Burden of Disease Study 2019” by Murray et al., The Lancet 2020; 396: 1223–49.)

Flowchart

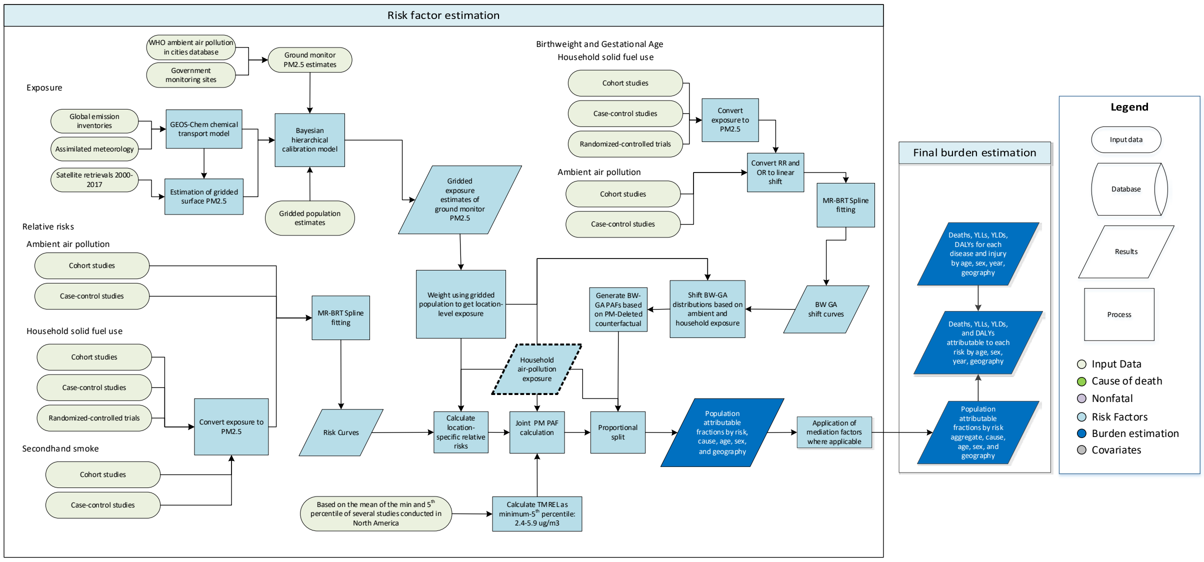

Input data and modelling strategy

Exposure

Definition

Exposure to ambient particulate matter pollution is defined as the population-weighted annual average mass concentration of particles with an aerodynamic diameter less than 2.5 micrometers ($PM_{2.5}$) in a cubic meter of air. This measurement is reported in µg/m^3.

Input data

The data used to estimate exposure to ambient particulate matter pollution comes from multiple sources, including satellite observations of aerosols in the atmosphere, ground measurements, chemical transport model simulations, population estimates, and land-use data. Table 1 summarizes exposure input data.

Table 1: Exposure Input Data

Input data	Exposure
Source count (total)	663
Number of countries with data	114

The following details the updates in methodology and input data used in GBD 2019.

$PM_{2.5}$ ground measurement database

Ground measurements used for GBD 2019 include updated measurements from sites included in 2017 and additional measurements from new locations. New and up-to-date data (mainly from the USA, Canada, EU, Bangladesh, China and USA embassies and consulates), were added to the data from the 2018 update of the WHO

Global Ambient Air Quality Database used in GBD 2017. The updated data included measurements of concentrations of PM_{10} and $PM_{2.5}$ from 10,408 ground monitors from 116 countries from 2010 to 2017. The majority of measurements were recorded in 2016 and 2017 (as there is a lag in reporting measurements, few data from 2018 or newer were available). Annual averages were excluded if they were based on less than 75% coverage within a year. If information on coverage was not available, then data were included unless there were already sufficient data within the same country (monitor density greater than 0.1).

For locations measuring only PM_{10}, $PM_{2.5}$ measurements were estimated from PM_{10}. This was performed using a hierarchy of conversion factors ($PM_{2.5}/PM_{10}$ ratios): (i) for any location a 'local' conversation factor was used, constructed as the ratio of the average measurements (of $PM_{2.5}$ and PM_{10}) from within 50km of the location of the PM_{10} measurement, and within the same country, if such measurements were available; (ii) if there was not sufficient local information to construct a conversion factor then a country-wide conversion factor was used; and (iii) if there was no appropriate information within a country, then a regional factor was used. In each case, to avoid the possible effects of outliers in the measured data (both $PM_{2.5}$ and PM_{10}), extreme values of the ratios were excluded (defined as being greater/lesser than the 95% and 5% quantiles of the empirical distributions of conversion factors). As with GBD 2013, 2015, 2016, and 2017 databases, in addition to values of $PM_{2.5}$ and whether they were direct measurement or converted from PM_{10}, the database also included additional information, where available, related to the ground measurements such as monitor geo-coordinates and monitor site type.

Satellite-based estimates

The global geophysical $PM_{2.5}$ estimates for the years 2000–2017 are from Hammer and colleagues Version V4.GL.03.NoGWR used at 0.1^ox0.1^o resolution (~11 x 11 km resolution at the equator).[1] The method is based on the algorithms of van Donkelaar and colleagues (2016) as used in GBD 2017,[2] with updated satellite retrievals, chemical transport modelling, and ground-based monitoring. The algorithm uses aerosol optical depth (AOD) from several updated satellite products (MAIAC, MODIS C6.1, and MISR v23), including finer resolution, increased global coverage, and improved long-term stability. Ground-based observations from a global sunphotometer network (AERONET version 3) are used to combine different AOD information sources. This is the first time that data from MAIAC at 1 km resolution was used to estimate $PM_{2.5}$ at the global scale. The GEOS-Chem chemical transport model with updated algorithms was used for geophysical relationships between surface $PM_{2.5}$ and AOD. Updates to the GEOS-Chem simulation included improved representation of mineral dust and secondary organic aerosol, as well as updated emission inventories. The resultant geophysical $PM_{2.5}$ estimates are highly consistent with ground monitors worldwide (R^2=0.81, slope = 1.03, n = 2541).

Population data

A comprehensive set of population data, adjusted to match UN2015 Population Prospectus, on a high-resolution grid was obtained from the Gridded Population of the World (GPW) database. Estimates for 2000, 2005, 2010, 2015, and 2020 were available from GPW version 4, with estimates for 1990 and 1995 obtained from the GPW version 3. These data are provided on a $0.0083^o \times 0.0083^o$ resolution. Aggregation to each $0.1^o \times 0.1^o$ grid cell was accomplished by summing the central 12 × 12 population cells. Populations estimates for 2001–2004, 2006–2009, 2011–2014 and 2016–2019 were obtained by interpolation using natural splines with knots placed at 2000, 2005, 2010, 2015, and 2020. This was performed for each grid cell.

Chemical transport model simulations

Estimates of the sum of particulate sulfate, nitrate, ammonium, and organic carbon and the compositional concentrations of mineral dust simulated using the GEOS Chem chemical transport model, and a measure combining elevation and the distance to the nearest urban land surface (as described in van Donkelaar and colleagues 2016[2] and Hammer and colleagues (submitted))[1] were available for 2000–2017 for each $0.1^o \times 0.1^o$ grid cell.

Modelling strategy

The following is a summary of the modelling approach, known as the Data Integration Model for Air Quality (DIMAQ) used in GBD 2015, 2016, 2017, and now in GBD 2019.[3,4]

Before the implementation of DIMAQ (ie, in GBD 2010 and GBD 2013), exposure estimates were obtained using a single global function to calibrate available ground measurements to a “fused” estimate of $PM_{2.5}$; the mean of satellite-based estimates and those from the TM5 chemical transport model, calculated for each $0.1^o \times 0.1^o$ grid cell. This was recognised to represent a tradeoff between accuracy and computational efficiency when utilising all the available data sources. In particular, the GBD 2013 exposure estimates were known to underestimate ground measurements in specific locations (see discussion in Brauer and colleagues, 2015).[5] This underestimation was largely due to the use of a single, global calibration function, whereas in reality the relationship between ground measurements and other variables will vary spatially.

In GBD 2015 and GBD 2016, coefficients in the calibration model were estimated for each country. Where data were insufficient within a country, information can be “borrowed” from a higher aggregation (region) and, if enough information is still not available, from an even higher level (super-region). Individual country-level estimates were therefore based on a combination of information from the country, its region, and its super-region. This was implemented within a Bayesian hierarchical modelling (BHM) framework. BHMs provide an extremely useful and flexible framework in which to model complex relationships and dependencies in data. Uncertainty can also be propagated through the model, allowing uncertainty arising from different components, both data sources and models, to be incorporated within estimates of uncertainty associated with the final estimates. The results of the modelling comprise a posterior distribution for each grid cell, rather than just a single point estimate, allowing a variety of summaries to be calculated. The primary outputs here are the median and 95% credible intervals for each grid cell. Based on the availability of ground measurement data, modelling and evaluation were focused on the year 2016.

The model used in GBD 2017 and GBD 2019 also included within-country calibration variation.[6] The model used for GBD 2019, henceforth referred to as DIMAQ2, provides a number of substantial improvements over the initial formulation of DIMAQ. In DIMAQ, ground measurements from different years were all assumed to have been made in the primary year of interest and then regressed against values from other inputs (eg, satellites, etc.) made in that year. In the presence of changes over time, therefore, and particularly in areas where no recent measurements were available, there was the possibility of mismatches between the ground measurements and other variables. In DIMAQ2, ground measurements were matched with other inputs (over time), and the (global-level) coefficients were allowed to vary over time, subject to smoothing that is induced by a first-order random walk process. In addition, the manner in which spatial variation can be incorporated within the model has developed: where there are sufficient data, the calibration equations can now vary (smoothly) both within and between countries, achieved by allowing the coefficients to follow (smooth) Gaussian processes. Where there are insufficient data within a country, to produce accurate equations, as before, information is borrowed from lower down the hierarchy and it is supplemented with information from the wider region.

DIMAQ2 as described above is used for all regions except for the north Africa and Middle East and sub-Saharan Africa super-regions, where there are insufficient data across years to allow the extra complexities of the new model to be implemented. In these super-regions, a simplified version of DIMAQ2 is used in which the temporal component is dropped.

Model evaluation

Model development and comparison was performed using within- and out-of-sample assessment. In the evaluation, cross-validation was performed using 25 combinations of training (80%) and validation (20%) datasets. Validation sets were obtained by taking a stratified random sample, using sampling probabilities based on the cross-tabulation of $PM_{2.5}$ categories (0-24.9, 25-49.9, 50-74.9, 75-99.9, 100+ $\mu g/m^3$) and super-regions, resulting in them having the same distribution of $PM_{2.5}$ concentrations and super-regions as the overall set of sites. The following metrics were

calculated for each training/evaluation set combination: for model fit – R^2 and deviance information criteria (DIC, a measure of model fit for Bayesian models); for predictive accuracy – root mean squared error (RMSE) and population weighted root mean squared error (PwRMSE). The median R^2 was 0.9, and the median PwRMSE was 10.1 μg/m^3.

All modelling was performed on the log-scale. The choice of which variables were included in the model was made based on their contribution to model fit and predictive ability. The following is a list of variables and model structures that were included in DIMAQ.

Continuous explanatory variables:

- (SAT) Estimate of $PM_{2.5}$ (in μg/m^3) from satellite remote sensing on the log-scale.
- (POP) Estimate of population for the same year as SAT on the log-scale.
- (SNAOC) Estimate of the sum of sulfate, nitrate, ammonium, and organic carbon simulated using the GEOS Chem chemical transport model.
- (DST) Estimate of compositional concentrations of mineral dust simulated using the GEOS-Chem chemical transport model.
- (EDxDU) The log of the elevation difference between the elevation at the ground measurement location and the mean elevation within the GEOS Chem simulation grid cell multiplied by the inverse distance to the nearest urban land surface.

Discrete explanatory variables:

- (LOC) Binary variable indicating whether exact location of ground measurement is known.
- (TYPE) Binary variable indicating whether exact type of ground monitor is known.
- (CONV) Binary variable indicating whether ground measurement is $PM_{2.5}$ or converted from PM_{10}.

Interactions*:*

- Interactions between the binary variables and the effects of SAT.

Random effects:

- Regional temporal (random walk) hierarchical random-effects on the intercept
- Regional hierarchical random-effects for the coefficient associated with SAT
- Regional hierarchical random-effects for the coefficient associated with POP
- Smoothed, spatially varying random-effects for the intercept
- Smoothed, spatially varying random-effects for the coefficient associated with SAT

Inference and prediction

Due to both the complexity of the models and the size of the data, notably the number of spatial predictions that are required, recently developed techniques that perform “approximate” Bayesian inference based on integrated nested Laplace approximations (INLA) were used.[7] Computation was performed using the R interface to the INLA computational engine (R-INLA). GBD 2019 also makes use of an innovation in the way that samples from the (Bayesian) model are used to represent distributions of estimated concentrations in each grid-cell. Here estimates, and distributions representing uncertainty, of concentrations for each grid are obtained by taking repeated (joint) samples from the posterior distributions of the parameters and calculating estimates based on a linear combination of those samples and the input variables.[8]

DIMAQ2 was used to produce estimates of ambient $PM_{2.5}$ for 1990, 1995, and 2010–2019 by matching the gridded estimates with the corresponding coefficients from the calibration. As there is a lag in reporting ambient air pollution based quantities, the input variables were extrapolated (as in GBD 2017), allowing estimates for 2018 and 2019 to be produced in the same way as other years and, crucially, allowing measures of uncertainty to be produced within the BHM framework rather than by using post-hoc approximations.

Estimates from the satellites and the GEOS-Chem chemical transport model in 2018 and 2019 were produced by extrapolating estimates from 2000–2017 using generalised additive models,[9] on a cell-by-cell basis, except in those grid cells that saw a >100% increase between 2016 and 2017, in which case only the 2000–2016 estimates were used for extrapolating, in order to avoid unrealistic and/or unjustified extrapolation of trends. Population estimates for 2018 and 2019 were obtained by interpolation as described above.

Alcohol estimation methods

(Adapted from “Global burden of 87 risk factors in 204 countries and territories, 1990-2019: a systematic analysis for the Global Burden of Disease Study 2019” by Murray et al., The Lancet 2020; 396: 1223–49.)

Flowchart

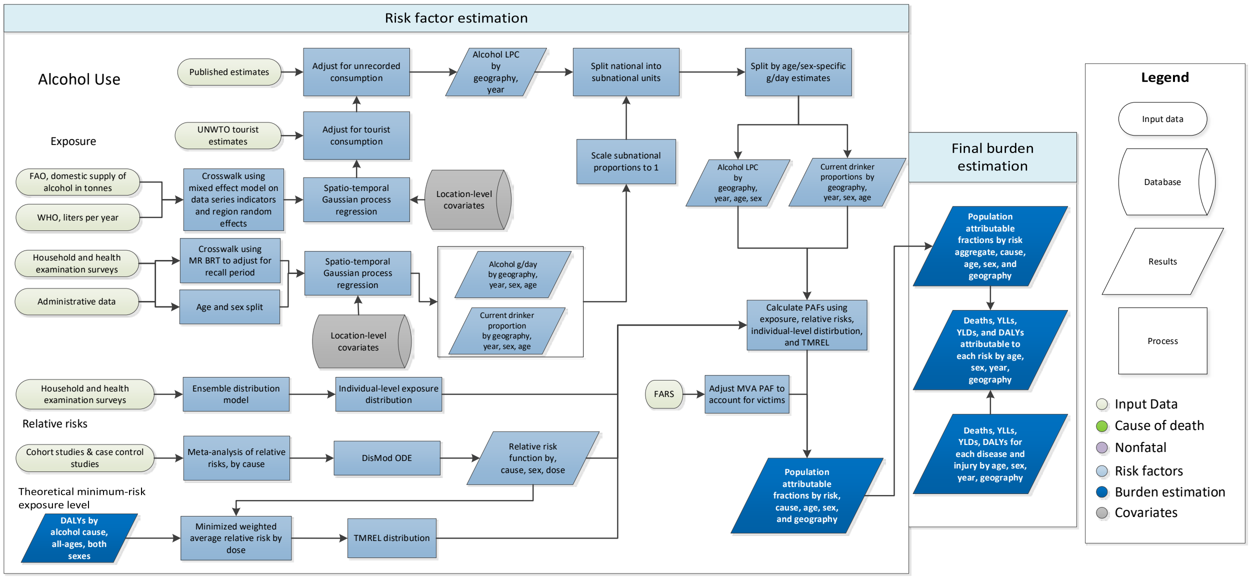

Input data and methodological summary

Definition

Exposure

We defined exposure as the grams per day of pure alcohol consumed among current drinkers. We constructed this exposure using the indicators outlined below:

1. Current drinkers, defined as the proportion of individuals who have consumed at least one alcoholic beverage (or some approximation) in a 12-month period.
2. Alcohol consumption (in grams per day), defined as grams of alcohol consumed by current drinkers, per day, over a 12-month period.
3. Alcohol litres per capita stock, defined in litres per capita of pure alcohol, over a 12-month period.

We also used three additional indicators to adjust alcohol exposure estimates to account for different types of bias:

1. Number of tourists within a location, defined as the total amount of visitors to a location within a 12-month period.
2. Tourists’ duration of stay, defined as the number of days resided in a hosting country.
3. Unrecorded alcohol stock, defined as a percentage of the total alcohol stock produced outside established markets.

Input data

A systematic review of the literature was performed to extract data on our primary indicators. The Global Health Exchange (GHDx), IHME’s online database of health-related data, was searched for population survey data containing participant-level information from which we could formulate the required alcohol use indicators on current drinkers and alcohol consumption. Data sources were included if they captured a sample representative of

the geographical location under study. We documented relevant survey variables from each data source in a spreadsheet and extracted using STATA 13.1 and R 3.3. A total of 6172 potential data sources were available in the GHDx, of which 5091 have been screened and 1125 accepted.

Table 1: Data inputs for exposure for alcohol use.

Input data	Exposure	Relative risk
Sources (total)	10513	495
Number of countries with data	199	-

Estimates of current drinking prevalence were split by age and sex where necessary. First, studies that reported prevalence for both sexes were split using a region-specific sex ratio estimated using MR-BRT. Second, where studies reported estimates across non-GBD age groups, these were split into standard five-year age groups using the global age pattern estimated by ST-GPR.

Table 2: MR-BRT sex splitting adjustment factors for current drinking

Data input	Gamma	Beta coefficient, log (95% CI)	Adjustment factor*
Female: Male	0	-0.16 (-0.17, -0.14)	0.85
Age < 50	0	0.06 (0.06, 0.06)	1.07
East Asia	0.36	-1.02 (-1.74, -0.29)	0.36
Southeast Asia	0.64	-1.06 (-2.34, 0.22)	0.35
Central Asia	0.41	-0.35 (-1.16, 0.46)	0.70
Central Europe	0.18	-0.21 (-0.58, 0.14)	0.80
Eastern Europe	0.10	-0.07 (-0.28, 0.14)	0.93
High-income Asia Pacific	1.27	-1.11 (-4.90, 2.68)	0.33
Western Europe	0.08	0.03 (-0.14, 0.20)	1.03
Southern Latin America	1.26	-0.67 (-4.18, 2.84)	0.51
High-income North America	0.09	-0.07 (-0.26, 0.11)	0.93
Caribbean	0.25	-0.52 (-1.02, -0.03)	0.59
Andean Latin America	0.76	-0.16 (-1.66, 1.34)	0.85
Central Latin America	0.30	-0.52 (-1.12, 0.08)	0.59
Tropical Latin America	0.08	-0.61 (-0.79, -0.44)	0.54
North Africa and Middle East	1.21	-1.44 (-3.91, 1.03)	0.24
South Asia	0.71	-1.17 (-2.57, 0.23)	0.31
Eastern sub-Saharan Africa	0.28	-0.53 (-1.10, 0.03)	0.58
Southern sub-Saharan Africa	0.20	-0.16 (-0.56, 0.23)	0.85
Western sub-Saharan Africa	0.32	-0.19 (-0.83, 0.45)	0.83
Oceania	0.94	-0.54 (-2.42, 1.34)	0.58

**Adjustment factor is the transformed beta coefficient in normal space and can be interpreted as the factor by which the alternative case definition is adjusted to reflect the ratio by which both-sex data points were split.*

To allow for the inclusion of data that did not meet our reference definition for current drinking, two crosswalks were performed using MR-BRT. The first crosswalk converted estimates of one-month drinking prevalence to what they would be if data represented estimates of 12-month drinking prevalence. This crosswalk incorporated two binary covariates: male and age ≥ 50. The second crosswalk converted estimates of one-week drinking prevalence to 12-month drinking prevalence. This crosswalk incorporated age < 20 and male as covariates. The covariates utilised in both crosswalks were included as both x and z covariates. A uniform prior of 0 was set as the upper bound for the beta coefficients to enforce the logical constraint that one-month and one-week prevalence could not be greater than 12-month prevalence.

Table 3: MR-BRT crosswalk adjustment factors for alcohol use current drinking model

Data input	Reference or alternative case definition	Gamma	Beta coefficient, logit (95% CI)
12-month prevalence	Ref	---	---
1-month prevalence	Alt	0.22	-0.60 (-1.05, -0.16)
Age ≥ 50		0.13	0.16 (-0.10, 0.43)
Male		0.29	0.01 (-0.57, 0.59)
1-week prevalence	Alt	0.46	-1.51 (-2.42, -0.59)
Age < 20		0.47	-0.29 (-1.34, 0.76)
Male		0.00	0.38 (0.15, 0.60)

The methods for modelling supply-side-level data were changed substantially from those used in GBD 2017. The raw data are domestic supply (WHO GISAH; FAO) and retail supply (Euromonitor) of litres of pure ethanol consumed. Domestic supply is calculated as the sum of production and imports, subtracting exports. The WHO and FAO sources were combined, so that FAO data were only used if there were no data available for that location-year from WHO. This was done because the WHO source takes into consideration FAO values when available. Since the WHO data are given in more granular alcohol types, the following adjustments were made:

$$LPC\ Pure\ Ethanol = 0.13 * \left(\frac{Wine}{0.973}\right)$$

$$LPC\ Pure\ Ethanol = 0.05 * \left(\frac{Beer}{0.989}\right)$$

$$LPC\ Pure\ Ethanol = 0.4 * \left(\frac{Spirits}{0.91}\right)$$

Three outliering strategies are used to omit implausible datapoints and data that created implausible model fluctuations. First, estimates from the current drinking model are used to calculate the grams of alcohol consumed per drinker per day. A point is outliered if the grams of pure ethanol per drinker per day for a given source-location-year is greater than 100 (approximately ten drinks). These thresholds were chosen by using expert knowledge about reasonable consumption levels. In the second round of outliering, the mean liters per capita value over a ten-year window is calculated. If a point is over 70% of that mean value away from the mean value, it is outliered. The 70% limit was chosen using histograms of these distances. Additionally, some manual outliering is performed to account for edge cases. Finally, data smoothing is performed by taking a three-year rolling mean over each location-year.

Next, an imputation to fill in missing years is performed for all series to remove compositional bias from our final estimates. Since the data from our main sources cover different time periods, by imputing a complete time series for each data series, we reduce the probability that compositional bias of the sources is leading to biased final estimates. To impute the missing years for each series, we model the log ratio of each pair of sources as a function of an intercept and nested random effects on super-region, region, and location. The appropriate predicted ratio is multiplied by the source that we do have, which generates an estimated value for the missing source. For some locations where there was limited overlap between series, the predicted ratio did not make sense, and a regional ratio was used.

Finally, variance was calculated both across series (within a location-year) as well as across years (within a location-source). Additionally, if a location-year had one imputed point, the variance was multiplied by 2. If a location-year had two imputed points, the variance was multiplied by 4. The average estimates in each location-year were the input to an ST-GPR model. This uses a mixed-effects model modelled in log space with nested location random effects.

We obtained data on the number of tourists and their duration of stay from the UNWTO.[3] We applied a crosswalk across different tourist categories, similar to the one used for the litres per capita data, to arrive at a consistent definition (ie, visitors to a country).

We obtained estimates on unrecorded alcohol stock from data available in WHO GISAH database,[2] consisting of 189 locations. For locations with no data available, the national or regional average was used.

For relative risks, in GBD 2016 we performed a systematic literature review of all cohort and case-control studies reporting a relative risk, hazard ratio, or odds ratio for any risk-outcome pairs studied in GBD 2016. Studies were included if they reported a categorical or continuous dose for alcohol consumption, as well as uncertainty measures for their outcomes, and the population under study was representative.

Modelling strategy

While population-based surveys provide accurate estimates of the prevalence of current drinkers, they typically underestimate real alcohol consumption levels.[10-12] As a result, we considered the litre per capita input to be a better estimate of overall volume of consumption. Per capita consumption, however, does not provide age- and sex-specific consumption estimates needed to compute alcohol-attributable burden of disease. Therefore, we use the age-sex pattern of consumption among drinkers modelled from the population survey data and the overall volume of consumption from FAO, GISAH, and Euromonitor to determine the total amount of alcohol consumed within a location. In the paragraphs below, we outline how we estimated each primary input in the alcohol exposure model, as well as how we combined these inputs to arrive at our final estimate of grams per day of pure alcohol. We estimated all models below using 1000 draws.

For data obtained through surveys, we used spatiotemporal Gaussian process regression (ST-GPR) to construct estimates for each location/year/age/sex. We chose to use ST-GPR due to its ability to leverage information across the nearby locations or time periods. We also modelled the alcohol litres per capita (LPC) data, as well as the total number of tourists, using ST-GPR.

Given the heterogeneous nature of the estimates on unrecorded consumption, as well as the wide variation across countries and time periods, we took 1000 draws from the uniform distribution of the lowest and highest estimates available for a given country. We did this to incorporate the diffuse uncertainty within the unrecorded estimates reported. We used these 1000 draws in the equation below.

We adjusted the alcohol LPC for unrecorded consumption using the following equation:

$$Alcohol\ LPC = \frac{Alcohol\ LPC}{(1 - \%\ Unrecorded)}$$

We then adjusted the estimates for alcohol LPC for tourist consumption by adding in the per capita rate of consumption abroad and subtracting the per capita rate of tourist consumption domestically.

$$Alcohol\ LPC_d = Unadjusted\ Alcohol\ LPC_d + Alcohol\ LPC_{Domestic\ consumption\ abroad} - Alcohol\ LPC_{Tourist\ consumption\ domestically}$$

$$Alcohol\ LPC_i = \frac{\sum_l Tourist\ Population_l * Proportion\ of\ tourists_{i,l} * Unadjusted\ Alcohol\ LPC_l * \frac{Average\ length\ of\ stay_{i,l}}{365} *}{Population_d}$$

where:

l *is the set of all locations,* i *is either* Domestic consumption abroad *or* Tourist consumption domestically,

and d *is a domestic location.*

After adjusting alcohol LPC by tourist consumption and unrecorded consumption for all location/years reported, sex-specific and age-specific estimates were generated by incorporating estimates modelled in ST-GPR for percentage of current drinkers within a location/year/sex/age, as well as consumption trends modelled in the ST-GPR grams per day model. We do this by first calculating the proportion of total consumption for a given location/year by age and sex, using the estimates of alcohol consumed per day, the population size, and the percentage of current drinkers. We then multiply this proportion of total stock for a given location/year/sex/age by the total stock for a given location/year to calculate the consumption in terms of litres per capita for a given location/year/sex/age. We then convert these estimates to be in terms of grams/per day. The following equations describe these calculations:

$$Proportion\ of\ total\ consumption_{l,y,s,a} = \frac{Alcohol\ \ g/day_{\ l,y,s,a} * \ Population_{l,y,s,a} * \ \%\ Current\ drinkers_{l,y,s,a}}{\sum_{s,a} Alcohol\ \ g/day_{l,y,s,a} * \ Population_{l,y,s,a} * \ \%\ Current\ drinkers_{l,y,s,a}}$$

$$Alcohol\ LPC_{l,y,s,a} = \frac{Alcohol\ LPC_{l,y} * \ Population_{l,y} * \ Proportion\ of\ total\ consumption_{l,y,s,a}}{\%\ Current\ drinkers_{l,y,s,a} * Population_{l,y,s,a}}$$

$$Alcohol\ g/day_{l,y,s,a} = Alcohol\ LPC_{l,y,s,a} * \frac{1000}{365}$$

where:

$l\ is\ a\ location, y\ is\ a\ year, s\ is\ a\ sex, and\ a\ is\ an\ age\ group.$

We then used the gamma distribution to estimate individual-level variation within location, year, sex, age drinking populations, following the recommendations of other published alcohol studies.[7,8] We chose parameters of the gamma distribution based on the mean and standard deviation of the 1,000 draws of alcohol g/day exposure for a given population. Standard deviation was calculated using the following formula.[15] We tested several alternative models using our data and found this model performed best.

$$standard\ deviation = mean * (0.087 * female + \ 1.171\ )$$

References

1. Food and Agriculture Organization of the United Nations (FAO). FAOSTAT Food Balance Sheets, October 2014. Rome, Italy: Food and Agriculture Organization of the United Nations (FAO).
2. World Health Organization (WHO). WHO Global Health Observatory - Recorded adult per capita alcohol consumption, Total per country. Geneva, Switzerland: World Health Organization (WHO).
3. UN World Tourism Organization (UNWTO). UN World Tourism Organization Compendium of Tourism Statistics 2015 [Electronic]. Madrid, Spain: UN World Tourism Organization (UNWTO), 2016.
4. Ramstedt, Mats. “How much alcohol do you buy? A comparison of self-reported alcohol purchases with actual sales.” *Addiction* 105.4 (2010): 649-654.
5. Stockwell, Tim, et al. “Under-reporting of alcohol consumption in household surveys: a comparison of quantity–frequency, graduated–frequency and recent recall.” *Addiction* 99.8 (2004): 1024-1033.
6. Kerr, William C., and Thomas K. Greenfield. “Distribution of alcohol consumption and expenditures and the impact of improved measurement on coverage of alcohol sales in the 2000 National Alcohol Survey.” *Alcoholism: Clinical and Experimental Research* 31.10 (2007): 1714-1722.
7. Taylor, Bruce, et al. “The more you drink, the harder you fall: a systematic review and meta-analysis of how acute alcohol consumption and injury or collision risk increase together.” *Drug and alcohol dependence* 110.1 (2010): 108-116.

8. Vinson, Daniel C., Guilherme Borges, and Cheryl J. Cherpitel. “The risk of intentional injury with acute and chronic alcohol exposures: a case-control and case-crossover study.” *Journal of studies on alcohol* 64.3 (2003): 350-357.
9. Vinson, Daniel C., et al. “A population-based case-crossover and case-control study of alcohol and the risk of injury.” *Journal of studies on alcohol*64.3 (2003): 358-366.
10. Fatal Accident Reporting System (FARS). National Highway Traffic Safety Administration, National Center for Statistics and Analysis Data Reporting and Information Division (NVS-424); 1985, 1990, 1995, 2000, 2005, 2010, 2015
11. Chen, Li-Hui, Susan P. Baker, and Guohua Li. “Drinking history and risk of fatal injury: comparison among specific injury causes.” Accident Analysis & Prevention 37.2 (2005): 245-251.
12. Bell, Nicole S., et al. “Self-reported risk-taking behaviors and hospitalization for motor vehicle injury among active duty army personnel.” American journal of preventive medicine 18.3 (2000): 85-95.
13. Margolis, Karen L., et al. “Risk factors for motor vehicle crashes in older women.” The Journals of Gerontology Series A: Biological Sciences and Medical Sciences 57.3 (2002): M186-M191.
14. Sorock, Gary S., et al. “Alcohol-drinking history and fatal injury in older adults.” Alcohol 40.3 (2006): 193-199.
15. Kehoe, Tara et al. “Determining the best population-level alcohol consumption model and its impact on estimates of alcohol-attributable harms.” Population health metrics 10 6. (2012)

Body mass index estimation methods

(Adapted from "Global burden of 87 risk factors in 204 countries and territories, 1990-2019: a systematic analysis for the Global Burden of Disease Study 2019" by Murray et al., The Lancet 2020; 396: 1223–49.)

Flowchart

Adult (Ages 20+) High Body-Mass Index: Data and Model Flow Chart

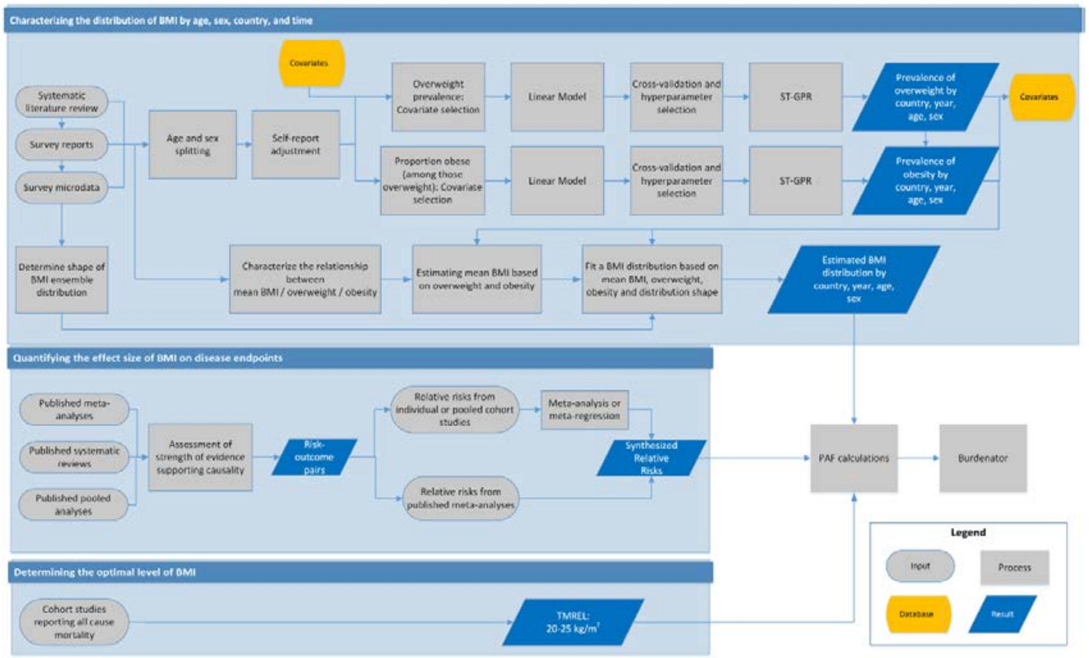

Childhood (Ages 2-19) High Body-Mass Index: Data and Model Flow Chart

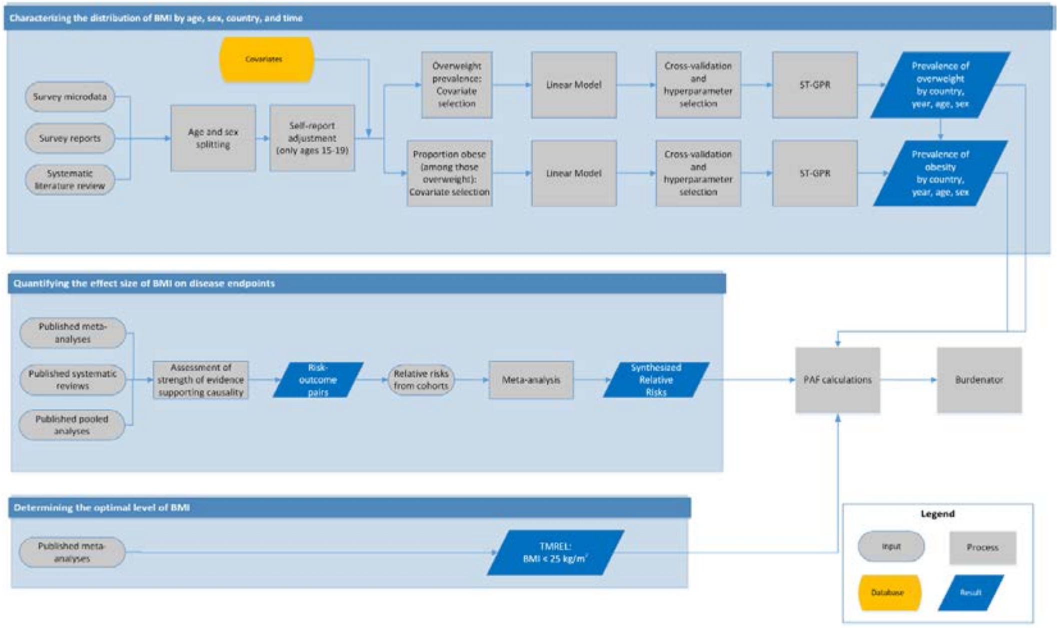

Input data and methodological summary

Case definitions

High body-mass index (BMI) for adults (ages 20+) is defined as BMI greater than 20 to 25 kg/m^2. High BMI for children (ages 1–19) is defined as being overweight or obese based on International Obesity Task Force standards.

Data sources

In GBD 2019, new data were added from sources included in the annual GHDx update of known survey series. We conducted a systematic review in GBD 2017 to identify studies providing nationally or subnationally representative estimates of overweight prevalence, obesity prevalence, or mean body-mass index (BMI). We limited the search to literature published between January 1, 2016, and December 31, 2016, to update the systematic literature search previously performed as part of GBD 2015.

The search for adults was conducted on 4 January 2017, using the following terms:

((("Body Mass Index"[Mesh] OR "Overweight"[Mesh] OR "Obesity"[Mesh]) AND ("Geographic Locations"[Mesh] NOT “United States”[Mesh]) AND ("humans"[Mesh] AND "adult"[MeSH]) AND ("Data Collection"[Mesh] OR "Health Services Research"[Mesh] OR "Population Surveillance"[Mesh] OR "Vital statistics"[Mesh] OR "Population"[Mesh] OR "Epidemiology"[Mesh] OR "surve*"[TiAb]) NOT (Comment[ptyp] OR Case Reports[ptyp] OR "hospital"[TiAb])) AND ("2016/01/01"[Date - Publication] : "2016/12/31"[Date - Publication]))

The search for children was conducted on 4 August 2016, using the following terms:
((("Body Mass Index"[Mesh] OR "Overweight"[Mesh] OR "Obesity"[Mesh]) AND ("Geographic Locations"[Mesh] NOT “United States”[Mesh]) AND ("humans"[Mesh] AND "child"[MeSH]) AND ("Data Collection"[Mesh] OR "Health Services Research"[Mesh] OR "Population Surveillance"[Mesh] OR "Vital statistics"[Mesh] OR "Population"[Mesh] OR "Epidemiology"[Mesh] OR "surve*"[TiAb]) NOT (Comment[ptyp] OR Case Reports[ptyp] OR "hospital"[TiAb])) AND ("2016/01/01"[Date - Publication] : "2016/12/31"[Date - Publication]))

Table 1: Data inputs for exposure for high body-mass index.

Input data	Exposure
Source count (total)	2022
Number of countries with data	190

Table 2: Data inputs for relative risks for high body-mass index.

Input data	Relative risk
Source count (total)	267
Number of countries with data	32

Eligibility criteria

We included representative studies providing data on mean BMI or prevalence of overweight or obesity among adults or children. For adults, studies were included if they defined overweight as BMI≥25 kg/m^2 and obesity as BMI≥30 kg/m^2, or if estimates using those cutoffs could be back-calculated from reported categories. For children (children ages 2–19), studies were included if they used International Obesity Task Force (IOTF) standards to define overweight and obesity thresholds. We only included studies reporting data collected after January 1, 1980. Studies were excluded if they used non-random samples (eg, case-control studies or convenience samples), conducted among specific subpopulations (eg, pregnant women, racial or ethnic minorities, immigrants, or individuals with specific diseases), used alternative methods to assess adiposity (eg, waist-circumference, skin-fold thickness, or hydrodensitometry), had sample sizes of less than 20 per age-sex group, or provided inadequate information on any of the inclusion criteria. We also excluded review articles and non-English-language articles.

Data collection process

Where individual-level survey data were available, we computed mean BMI using weight and height. We then used BMI to determine the prevalence of overweight and obesity. For individuals aged over 19 years, we considered them to be overweight if their BMI was greater than or equal to 25 kg/m^2, and obese if their BMI was greater than or equal to 30 kg/m^2. For individuals aged 2 to 19 years, we used monthly IOTF cutoffs[2] to determine overweight and obese status when age in months was available. When only age in years was available, we used the cutoff for the midpoint of that year. Obese individuals were also considered to be overweight. We excluded studies using the World Health Organization (WHO) standards or country-specific cutoffs to define childhood overweight and obesity. At the individual level, we considered BMI<10 kg/m^2 and BMI>70 kg/m^2 to be biologically implausible and excluded those observations.

The rationale for choosing to use the IOTF cutoffs over the WHO standards has been described elsewhere.[1] Briefly, the IOTF cutoffs provide consistent child-specific standards for ages 2–18 derived from surveys covering multiple countries. By contrast, the WHO growth standards apply to children under age 5, and the WHO growth reference applies to children ages 5–19. The WHO growth reference for children ages 5–19 was derived from United States data, which are less representative than the multinational data used by IOTF. Additionally, the switch between references at age 5 can produce artificial discontinuities. Given that we estimate global childhood overweight and obesity for ages 2–19 (with ages 19 using standard adult cutoffs), the IOTF cutoffs were preferable. Additionally, we found that IOTF cutoffs were more commonly used in scientific literature covering childhood obesity.

From report and literature data, we extracted data on mean BMI, prevalence of overweight, and prevalence of obesity, measures of uncertainty for each, and sample size, by the most granular age and sex groups available. Additionally, we extracted the same study-level covariates as were extracted from microdata (measurement, urbanicity, and representativeness), as well as location and year.

In addition to the primary indicators described above, we extracted relevant survey-design variables, including primary sampling unit, strata, and survey weights, which were used to tabulate individual-level microdata and produce accurate measures of uncertainty. We extracted three study-level covariates: 1) whether height and weight data were measured or self-reported; 2) whether the study was predominantly conducted in an urban area, rural area, or both; and 3) the level of representativeness of the study (national or subnational).

Finally, we extracted relevant demographic indicators, including location, year, age, and sex. We estimated the standard error of the mean from individual-level data, where available, and used the reported standard error of the mean for published data. When multiple data sources were available for the same country, we included all of them in our analysis. If data from the same data source were available in multiple formats such as individual-level data and tabulated data, we used individual-level data.

Modelling strategy

Age and sex splitting

Any report or literature data provided in age groups wider than the standard five-year age groups or as both sexes combined were split using the approach used by Ng and colleagues.[2] Briefly, age-sex patterns were identified using sources with data on multiple age-sex groups and these patterns were applied to split aggregated report and literature data. Uncertainty in the age-sex split was propagated by multiplying the standard error of the data by the square root of the number of splits performed. We did not propagate the uncertainty in the age pattern and sex pattern used to split the data as they seemed to have small effect.

Self-report bias adjustment

We included both measured and self-reported data. We tested for bias in self-report data compared to measured data, which is considered to be the gold-standard. There was no clear direction of bias for children ages 2–14, so for these age groups we only included measured data. For individuals ages 15 and above, we *adjusted self-reported data for overweight prevalence and obesity prevalence. In GBD 2017, the self-report bias adjustment used a nested hierarchical mixed-effects regression model. This approach was updated in GBD 2019 to utilise the power of MR-*

BRT. For both overweight and obesity, we fit sex-specific MR-BRT models on the logit difference between measured and self-reported with a fixed effect on super-region. The bias coefficients derived from these two models are in Table 1 and 2.

Table 1: MR-BRT self-report crosswalk adjustment factors for overweight prevalence

Model	Data input	Reference or alternative case definition	Gamma	Beta coefficient, logit (95% CI)
Females	Measured data	Ref	0.26	---
	Self-reported data (southeast Asia, east Asia, and Oceania)	Alt		-0.53 (-1.03, -0.04)
	Self-reported data (central Europe, eastern Europe, and central Asia)	Alt		-0.20 (-0.69, 0.30)
	Self-reported data (high-income)	Alt		-0.25 (-0.75, 0.24)
	Self-reported data (Latin America and Caribbean)	Alt		-0.19 (-0.69, 0.31)
	Self-report data (north Africa and Middle East)	Alt		-0.38 (-0.89,0.11)
	Self-report data (south Asia)	Alt		0.36 (-0.14, 0.85)
	Self-report data (sub-Saharan Africa)	Alt		-0.26 (-0.76, 0.24)
Males	Measured data	Ref	0.43	---
	Self-reported data (southeast Asia, east Asia, and Oceania)	Alt		-0.36 (-1.17, 0.50)
	Self-reported data (central Europe, eastern Europe, and central Asia)	Alt		-0.03 (-0.84, 0.82)
	Self-reported data (high-income)	Alt		0.05 (-0.77, 0.87)
	Self-reported data (Latin America and Caribbean)	Alt		-0.02 (-0.84, 0.81)
	Self-report data (north Africa and Middle East)	Alt		-0.21 (-1.04, 0.61)
	Self-report data (south Asia)	Alt		0.53 (-0.28, 1.37)
	Self-report data (sub-Saharan Africa)	Alt		-0.27 (-1.09, 0.55)

Table 2: MR-BRT self-report crosswalk adjustment factors for obesity prevalence

Model	Data input	Reference or alternative case definition	Gamma	Beta coefficient, logit (95% CI)
Females	Measured data	Ref	0.38	---
	Self-reported data (southeast Asia, east Asia, and Oceania)	Alt		-0.11 (-0.86, 0.64)
	Self-reported data (central Europe, eastern Europe, and central Asia)	Alt		-0.95 (-1.70, -0.19)
	Self-reported data (high-income)	Alt		-0.42 (-1.16, 0.34)
	Self-reported data (Latin America and Caribbean)	Alt		-0.41 (-1.16, 0.34)
	Self-report data (north Africa and Middle East)	Alt		-0.48 (-1.23, 0.27)
	Self-report data (south Asia)	Alt		0.50 (-0.25, 1.26)
	Self-report data (sub-Saharan Africa)	Alt		-0.41 (-1.16, 0.34)
Males	Measured data	Ref	0.74	
	Self-reported data (southeast Asia, east Asia, and Oceania)	Alt		0.04 (-1.41, 1.53)

	Self-reported data (central Europe, eastern Europe, and central Asia)	Alt		-0.79 (-2.25, 0.71)
	Self-reported data (high-income)	Alt		-0.13 (-1.58, 1.40)
	Self-reported data (Latin America and Caribbean)	Alt		-0.26 (-1.70, 1.21)
	Self-report data (north Africa and Middle East)	Alt		-0.33 (-1.77, 1.16)
	Self-report data (south Asia)	Alt		0.66 (-0.78, 2.15)
	Self-report data (sub-Saharan Africa)	Alt		-0.41 (-1.86, 1.08)

Prevalence estimation for overweight and obesity

After adjusting for self-report bias and splitting aggregated data into five-year age-sex groups, we used spatiotemporal Gaussian process regression (ST-GPR) to estimate the prevalence of overweight and obesity. This modelling approach has been described in detail elsewhere.

The linear model, which when added to the smoothed residuals forms the mean prior for GPR is as follows:

$$\text{logit}(\text{overweight})_{c,a,t} = \beta_0 + \beta_1 \text{energy}_{c,t} + \beta_2 \text{SDI}_{c,t} + \beta_3 \text{vehicles}_{c,t} + \beta_4 \text{agriculture}_{c,t} + \sum_{k=5}^{21} \beta_k I_{A[a]} + \alpha_s + \alpha_r + \alpha_c$$

$$\text{logit}(\text{obesity/overweight})_{c,a,t} = \beta_0 + \beta_1 \text{energy}_{c,t} + \beta_2 \text{SDI}_{c,t} + \beta_3 \text{vehicles}_{c,t} + \sum_{k=4}^{21} \beta_k I_{A[a]} + \alpha_s + \alpha_r + \alpha_c$$

where energy *is ten-year lag-distributed energy consumption per capita,* SDI *is a composite index of development including lag-distributed income per capita, education, and fertility*, vehicles *is is the number of two- or four-wheel vehicles per capita, and* agriculture *is the proportion of the population working in agriculture.* $I_{A[a]}$ is a dummy variable indicating specific age group A that the prevalence point captures, and α_s, α_r, and α_c are super-region, region, and country random intercepts, respectively. Random effects were used in model fitting but were not used in prediction.

We tested all combinations of the following covariates to see which performed best in terms of in-sample AIC for the overweight linear model and the obesity as a proportion of overweight linear model: ten-year lag-distributed energy per capita, proportion of the population living in urban areas, SDI, lag-distributed income per capita, educational attainment (years) per capita, proportion of the population working in agriculture, grams of sugar adjusted for energy per capita, grams of sugar not adjusted for energy per capita, and the number of two- or four-wheeled vehicles per capita. We selected these candidate covariates based on theory as well as reviewing covariates used in other publications. The final linear model was selected based on 1) if the direction of covariates matched what is expected from theory, 2) all the included covariates were significant, and 3) minimising in-sample AIC. The covariate selection process was performed using the dredge package in R.

Estimating mean BMI

To estimate the mean BMI for adults in each country, age, sex, and time period 1980–2019, we first used the following nested hierarchical mixed-effects model, fit using restricted maximum likelihood on data from sources containing estimates of all three indicators (prevalence of overweight, prevalence of obesity, and mean BMI), in order to characterise the relationship between overweight, obesity, and mean BMI:

$$\log(\text{BMI}_{c,a,s,t}) = \beta_0 + \beta_1 \text{ow}_{c,a,s,t} + \beta_2 \text{ob}_{c,a,s,t} + \beta_3 \text{sex} + \sum_{k=4}^{20} \beta_k I_{A[a]} + \alpha_s(1 + \text{ow}_{c,a,s,t} + \text{ob}_{c,a,s,t}) + \alpha_r(1 + \text{ow}_{c,a,s,t} + \text{ob}_{c,a,s,t}) + \alpha_c(1 + \text{ow}_{c,a,s,t} + \text{ob}_{c,a,s,t}) + \epsilon_{c,a,s,t}$$

where $ow_{c,a,s,t}$ *is the prevalence of overweight in country c, age a, sex s, and year t,* $ob_{c,a,s,t}$ *is the prevalence of obesity in country c, age a, sex s, and year t,* sex *is a fixed effect on sex,* $I_{A[a]}$ *is an indicator variable for age, and* α_s, α_r, *and* α_c *are random effects at the super-region, region, and country, respectively. The model was run in Stata 13.*

We applied 1000 draws of the regression coefficients to the 1000 draws of overweight prevalence and obesity prevalence produced through ST-GPR to estimate 1000 draws of mean BMI for each country, year, age, and sex. This approach ensured that overweight prevalence, obesity prevalence, and mean BMI were correlated at the draw level and uncertainty was propagated.

Estimating BMI distribution

We used the ensemble distribution approach described in the GBD 2019 Risk Factors paper. We fit ensemble weights by source and sex, with source- and sex-specific weights averaged across all sources included to produce the final global weights. The ensemble weights were fit on measured microdata. The final ensemble weights were exponential = 0.002, gamma = 0.028, inverse gamma = 0.085, log-logistic = 0.187, Gumbel = 0.220, Weibull = 0.011, log-normal = 0.058, normal = 0.012, beta = 0.136, mirror gamma = 0.008, and mirror Gumbel = 0.113.

One thousand draws of BMI distributions for each location, year, age group, and sex estimated were produced by fitting an ensemble distribution using 1000 draws of estimated mean BMI, 1000 draws of estimated standard deviation, and the ensemble weights. Estimated standard deviation was produced by optimising a standard deviation to fit estimated overweight prevalence draws and estimated obesity prevalence draws.

References

1.) Cole, TJ, and T Lobstein. "Extended International (IOTF) Body Mass Index Cut-Offs for Thinness, Overweight and Obesity." Pediatric Obesity 2012; 7(4): 284–94.
2.) Ng M, Fleming T, Robinson M, et al. Global, regional, and national prevalence of overweight and obesity in children and adults during 1980–2013: a systematic analysis for the Global Burden of Disease Study 2013. The Lancet 2014; 384: 766–81.
3.) Angelantonio ED, Bhupathiraju SN, Wormser D, et al. Body-mass index and all-cause mortality: individual-participant-data meta-analysis of 239 prospective studies in four continents. The Lancet 2016; 388: 776–86.

Education estimation methods

(Adapted from "Global age-sex-specific fertility, mortality, healthy life expectancy (HALE), and population estimates in 204 countries and territories,1950-2019: a comprehensive demographic analysis for the Global Burden of Disease Study 2019" by Wang et al., The Lancet 2020; 396: 1160–203.)

We based our estimates of average years of education on a collection of 3675 censuses and household surveys. The approach we used to estimate educational attainment, which we describe below, was based on previously published methods.[1] Each data source we used had information about the distribution of educational attainment by country or territory, year, sex, and five-year or ten-year age group. We only used data sources that included information on the distribution of educational attainment by country or territory, year, sex, and five-year or ten-year age groups. Some sources only provided education data for multi-year bins (eg, the percentage of the population with between two and five years of completed schooling), so for those sources we split data into single-year distributions from 0 to 18 years based on a database of 1226 sources that did report single years of educational attainment. We used the average of the 12 single-year distributions that were closest in terms of geographical proximity and year. From each data source, we calculated mean years of schooling by age and sex.

Next, we used age-cohort imputation to carry educational attainment in observed cohorts forward through time, since education levels are relatively constant after age 25. For datapoints from cohorts aged 25 or older, we extrapolated the data forward and backward in time so all year-age combinations in that cohort contained that data (eg, a datapoint for a cohort aged 40–44 in 1995 was projected forward for 45–49-year-olds in 2000, 50–54-year-olds in 2005, etc. and backward for 35–39-year-olds in 1990, 30–34-year-olds in 1985, etc.).

After imputation, we fit age-period models to all the original input data and to the imputed cohort data. This allowed us to estimate a complete single-year series of educational attainment from 1950 through 2019 by age, sex, and location.

For each sex and GBD location, we calculated the mean level of educational attainment of the country-age-year-specific population$Edu_{c,a,s,t}$, using the following formula:

$$logit\left(\frac{Edu_{c,a,s,t}}{Edu_{max_a}}\right) = \beta_{s,r} + \delta_{s,r}\,Year + I_{s,r} + \propto_{c,a,s}$$

$$\alpha_{c,a,s} \sim N(0, \sigma_\alpha^2)$$

where:

c is location, a is age, s is sex, t is time

Edu_{max_a} is the maximum mean educational attainment for each age group, defined as 3 for ages 5-9, 8 for ages 10-14, 13 for ages 15-19, and 18 for all age groups 20-24 and up

$\beta_{s,r}$is a sex- and region-specific intercept

$\delta_{s,r}$ captures the linear secular trend for each sex and region

$I_{s,r}$is a natural spline on age to capture the non-linear age pattern by sex and region, with knots at 45 and 65 years of age

$\propto_{c,s}$ is a country-sex-specific random intercept.

Last, we used GPR to smooth the age-period model residuals. This step allowed us to account for uncertainty in each datapoint and combine data and model uncertainty to estimate UIs.

References

1. Gakidou E, Cowling K, Lozano R, Murray CJL. Increased educational attainment and its effect on child mortality in 175 countries between 1970 and 2009: a systematic analysis. Lancet Lond Engl 2010; 376: 959–74.

High LDL cholesterol estimation methods

(Adapted from "Global burden of 87 risk factors in 204 countries and territories, 1990-2019: a systematic analysis for the Global Burden of Disease Study 2019" by Murray et al., The Lancet 2020; 396: 1223–49.)

Flowchart

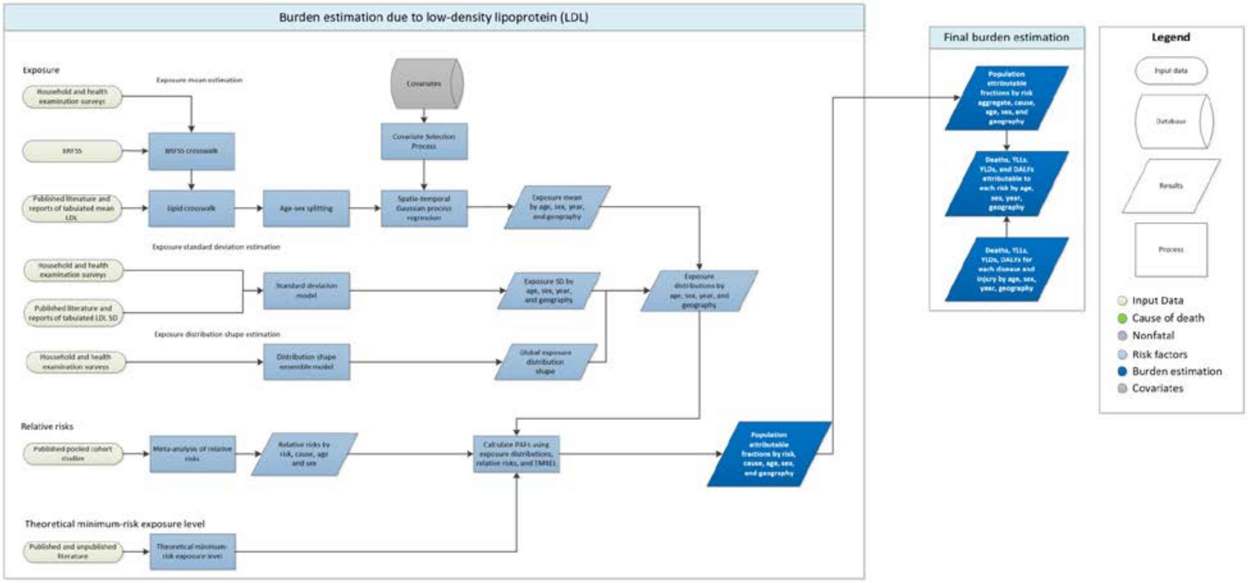

Input data and methodological summary

Exposure

Case definition

In earlier iterations of the GBD study, we estimated burden attributable to total cholesterol. Beginning in GBD 2017, we modelled blood concentration of low-density lipoprotein (LDL) in units of mmol/L.

Input data

We used data on blood levels for low-density lipoprotein, total cholesterol, triglyceride, and high-density lipoprotein from literature and from household survey microdata and reports. We adjusted data for total cholesterol, triglycerides, and high-density lipoprotein using the correction approach described in the Lipid Crosswalk section below. Counts of the data inputs used for GBD 2019 are show in Tables 1 and 2 below. Details of inclusion and exclusion criteria and data processing steps follow.

Table 1: Data inputs for exposure for low-density lipoprotein

Input data	Exposure
Total sources	711
Number of countries with data	145

Table 2: Data inputs for relative risks for low-density lipoprotein

Input data	Relative risk
Source count (total)	1

Inclusion criteria

Studies were included if they were population-based and measured total LDL, total cholesterol (TC), high-density

lipoprotein (HDL), and/or triglycerides (TG) were available from blood tests or if LDL was calculated using the Friedewald equation. We assumed the data were representative of the location if the geography or population chosen were not related to the diseases and if it was not an outlier compared to other data in the country or region.

Outliers
Data were utilised in the modelling process unless an assessment of data strongly suggested that the data were biased. A candidate source was excluded if the quality of study did not warrant a valid estimate because of selection (non-representative populations) or if the study did not provide methodological details for evaluation. In a small number of cases, a data point was considered to be an outlier candidate if the level was implausibly low or high based on expert judgement and other country data.

Data extraction
Where possible, individual-level data on LDL estimates were extracted from survey microdata and these were collapsed across demographic groupings to produce mean estimates in the standard GBD five-year age-sex groups. If microdata were unavailable, information from survey reports or from literature were extracted along with any available measure of uncertainty including standard error, uncertainty intervals, and sample size. Standard deviations were also extracted. Where LDL was reported split out by groups other than age, sex, location, and year (eg, by diabetes status), a weighted mean was calculated.

Lipid crosswalk
Total cholesterol consists of three major components: LDL, HDL, and TG. LDL is often calculated for an individual using the Friedewald equation, shown below:

$$LDL = TC - \left(HDL + \frac{TGL}{2.2}\right)$$

We utilised this relationship at the individual level to impute the mean LDL for a study population when only data on TC, HDL, and TGL were available. Because studies report different combinations of TC, HDL, and TGL, we constructed a single regression to utilise all available data to evaluate the relationship between each lipid and LDL at the population level. We used the following regression:

$$LDL = ind_{tc}\beta_1 TC - \left(ind_{hdl}\beta_2 HDL + ind_{tgl}\beta_3 TGL\right) + \sum \alpha_l I_l$$

Where ind_{tc}, ind_{hdl}, and ind_{tgl} are indicator variables for whether data are available for a given lipid, I_l is an indicator variable a given set of available lipids l. α_l is a unique intercept for each set of available lipid combinations. For example, for sources that only reported TC and HDL, $\alpha_{l=TC,HDL}$ should account for the missing lipid data, ie, TGL. The form of this regression allows us to estimate the betas for each lipid using all available data. As a sensitivity analysis, we also ran separate regressions for each set of available lipids and found that the single regression method had much lower root-mean-squared error. We found almost no relationship between LDL and HDL or TGL when TC was not available, so only studies that reported TC were adjusted to LDL.

Incorporating United States prevalence data

Survey reports and literature often report information only about the prevalence, but not the level, of hypercholesterolemia in the population studied. These sources were not used to model LDL, with the exception of data from the Behavioral Risk Factors Surveillance System (BRFSS) because of the availability of a similarly structured exam survey covering the identical population (NHANES). BRFSS is a telephone survey conducted in the United States for all counties. It collects self-reported diagnosis of hypercholesterolemia. These self-reported values of prevalence of raised total cholesterol in each age group, sex, US state, and year were used to predict a mean total cholesterol for the same strata with a regression using data from the National Health and Nutrition Examination Survey, a nationally representative health examination survey of the US adult population. The regression was:

$$TC_{l,a,t,s} = \beta_0 + \beta_1 prev_{l,a,t,s}$$

where $TC_{l,a,t,s}$ *is the location, age, time, and sex specific mean total cholesterol and* $prev_{l,a,t,s}$ *is the location, age, time, and sex specific prevalence of raised total cholesterol. The coefficients for both models are reported in Table 1.*

Table 3. Coefficients in the sex-specific US states TC prediction models

Term	Male model	Female model
Intercept	4.23	4.36
Prevalence	6.25	5.22

Out of sample RMSE was used to quantify the predictive validity of the model. The regression was repeated 10 times for each sex, each time randomly holding out 20% of the data. The RMSEs from each holdout analysis were averaged to get the average out of sample RMSE. The results of this holdout analysis are reported in Table 2. Total cholesterol estimates were crosswalked to LDL using the lipid crosswalk reported above.

Table 4. Out of sample RMSEs of the sex-specific US states TC prediction models

	Male model	Female model
Out of sample RMSE	0.21 mmol/L	0.20 mmol/L

Age and sex splitting

Prior to modelling, data provided in age groups wider than the GBD five-year age groups were processed using the approach outlined in Ng and colleagues.[2] Briefly, age-sex patterns were identified using person-level microdata (58 sources), and estimate age-sex-specific levels of total cholesterol from aggregated results reported in published literature or survey reports. In order to incorporate uncertainty into this process and borrow strength across age groups when constructing the age-sex pattern, we used a model with auto-regression on the change in mean LDL over age groups:

$$\mu_a = \mu_{a-1} + \omega_a$$
$$\omega_a \sim N(\omega_{a-1}, \tau)$$

Where μ_a is the mean predicted value for age group a, μ_{a-1} is the mean predicted value for the age group previous to age group a, ω_a is the difference in mean between age group *a* and age group *a-1*, ω_{a-1} is the difference between age group *a-1* and age group *a-2*, and τ is a user-input prior on how quickly the mean LDL changes for each unit increase in age. We used a τ of 0.05 mmol/L for this model. Draws of the age-sex pattern were combined with draws of the input data needing to be split in order to calculate the new variance of age-sex-split data points.

Modelling strategy

Exposure estimates were produced from 1980 to 2019 for each national and subnational location, sex, and for each five-year age group starting from 25. As in GBD 2017, we used a spatiotemporal Gaussian process regression (ST-GPR) framework to model the mean LDL at the location-, year-, age-, and sex- level. Details of the ST-GPR method used in GBD 2019 can be found elsewhere in the appendix.

Covariate selection

The first step of the ST-GPR framework requires the creation of a linear model for predicting LDL at the location-, year-, age-, sex- level. Covariates for this model were selected in two stages. First a list of variables with an expected causal relationship with LDL was created based on significant association found within high-quality

prospective cohort studies reported in the published scientific literature. The second stage in covariate selection was to test the predictive validity of every possible combination of covariates in the linear model, given the covariates selected above. This was done separately for each sex. Predictive validity was measured with out of sample root-mean-squared error.

In GBD 2016, the linear model with the lowest root-mean-squared error for each sex was then used in the ST-GPR model. Beginning in GBD 2017, we used an ensemble model of the 50 models with the lowest root-mean-squared error for each sex. This allows us to utilise covariate information from many plausible linear mixed-effects models. The 50 models were each used to predict the mean LDL for every age, sex, location, and year, and the inverse-RMSE-weighted average of this set of 50 predictions was used as the linear prior. The relative weight contributed by each covariate is plotted by sex in Figure 2.

Figure 2. Results of the ensemble linear model covariate selection

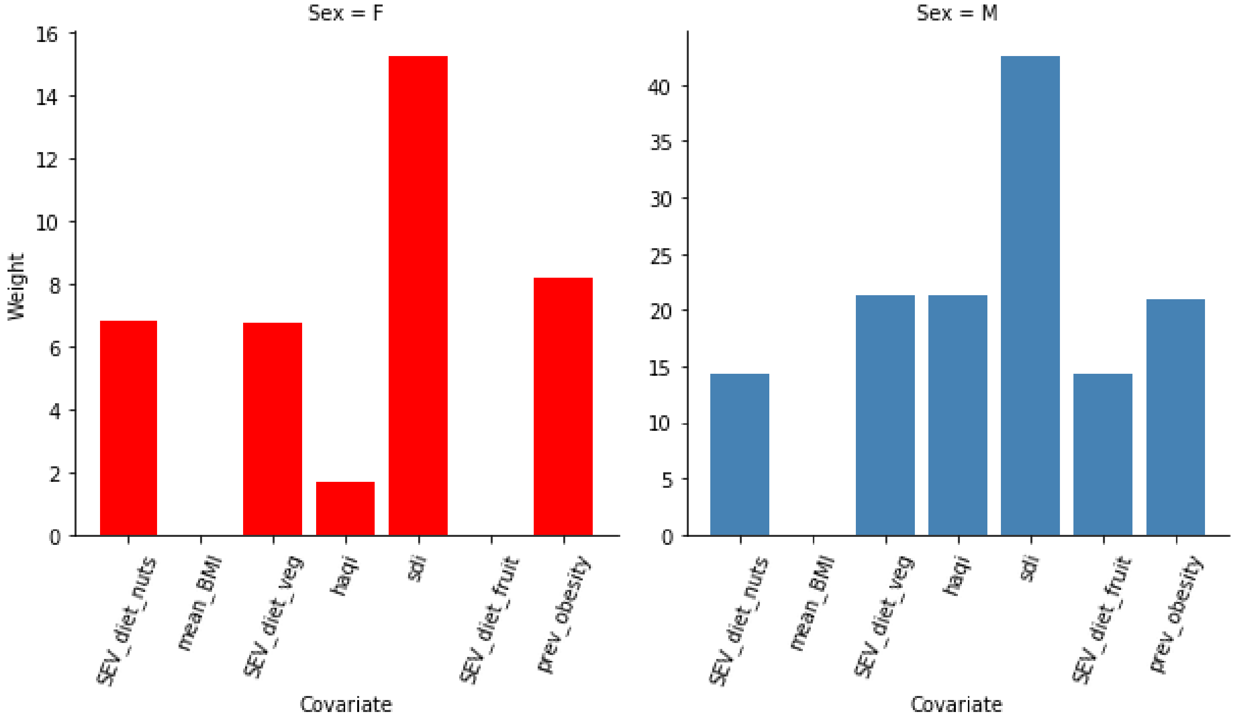

The results of the ensemble linear model were used for the first stage in an ST-GPR model. The result of the ST-GPR model are estimates of the mean LDL for each age, sex, location, and year.

Estimate of standard deviation

The standard deviation of LDL within a population was estimated for each national and subnational location, sex, and five-year age group starting from age 25 using the standard deviation from person-level and some tabulated data sources. Person-level microdata accounted for 3009 of the total 4001 rows of data on standard deviation. The remaining 992 rows came from tabulated data. Tabulated data were only used to model standard deviation if they were sex-specific and five-year-age-group-specific and reported a population standard deviation LDL. The LDL standard deviation function was estimated using a linear regression:

$$\log\left(SD_{c,a,t,s}\right) = \beta_0 + \beta_1 \log(mean\_LDL_{c,a,t,s}) + \beta_4 sex + \sum_{k=2}^{16} \beta_k I_{A[a]}$$

where $mean\_LDL_{c,a,t,s}$ is the country-, age-, time-, and sex-specific mean LDL estimate from ST-GPR, and $I_{A[a]}$ is a dummy variable for a fixed effect on a given five-year age group.

Distribution shape modelling

The shape of the distribution of LDL was estimated using all available person-level microdata sources, which was a

subset of the input data into the modelling process. The distribution shape modelling framework for GBD 2019 is detailed in the GBD 2019 Risk Factors paper. Briefly, an ensemble distribution created from a weighted average of distribution families was fit for each individual microdata source, separately by sex. The weights for the distribution families for each individual source were then averaged and weighted to create a global ensemble distribution for each sex.

References

1. Roth GA, Fihn SD, Mokdad AH, Aekplakorn W, Hasegawa T, Lim SS. High total serum cholesterol, medication coverage and therapeutic control: an analysis of national health examination survey data from eight countries. *Bull World Health Organ* 2011; **89**: 92–101.

2 . Ng M, Fleming T, Robinson M, *et al.* Global, regional, and national prevalence of overweight and obesity in children and adults during 1980–2013: a systematic analysis for the Global Burden of Disease Study 2013. *The Lancet* 2014; **384**: 766–81.

3 . Boekholdt SM, Hovingh GK, Mora S, *et al.* Very Low Levels of Atherogenic Lipoproteins and the Risk for Cardiovascular EventsA Meta-Analysis of Statin Trials. *J Am Coll Cardiol* 2014; **64**: 485–94.

4. Sabatine MS, Giugliano RP, Keech AC, et al. Evolocumab and Clinical Outcomes in Patients with Cardiovascular Disease. *N Engl J Med*. 2017; **376**:1713-1722.

5. Wilson PF, D'Agostino RB, Levy D, Belanger AM, Silbershatz H, Kannel WB. Prediction of Coronary Heart Disease Using Risk Factor Categories. *Circulation*. 1998; **97**:1837-1847.

6. Singh GM, Danaei G, Farzadfar F, *et al.* The age-specific quantitative effects of metabolic risk factors on cardiovascular diseases and diabetes: a pooled analysis. *PloS One* 2013; **8**: e65174.

High systolic blood pressure estimation methods

(Adapted from "Global burden of 87 risk factors in 204 countries and territories, 1990-2019: a systematic analysis for the Global Burden of Disease Study 2019" by Murray et al., The Lancet 2020; 396: 1223–49.)

Flowchart

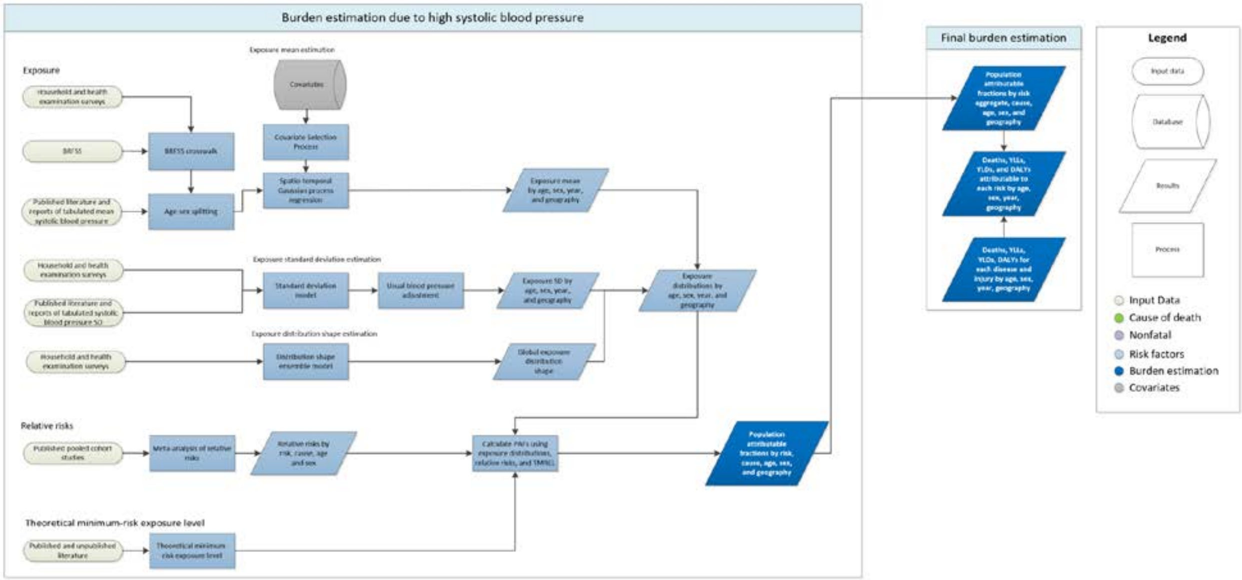

Input data and methodological summary

Case definition

Brachial systolic blood pressure in mmHg.

Input data

We utilised data on mean systolic blood pressure from literature and from household survey microdata and reports (e.g. STEPS, NHANES). For GBD 2019, we did not carry out a systematic review of the literature for new data. Counts of the data inputs used for GBD 2019 are show in Tables 1 and 2 below. Details of inclusion and exclusion criteria and data processing steps follow.

Table 1: Data inputs for exposure for systolic blood pressure.

Input data	Exposure
Total sources	1112
Number of countries with data	166

Table 2: Data inputs for relative risks for systolic blood pressure.

Input data	Relative risk
Source count (total)	3

Inclusion criteria
Studies were included if they were population-based and directly measured systolic blood pressure using a sphygmomanometer. We assumed the data were representative if the geography or the population were not selected because it was related to hypertension or hypertensive outcomes.

Outliers
Data were utilised in the modelling process unless an assessment strongly suggested that the source was biased. A candidate source was excluded if the quality of study did not warrant a valid estimate because of selection (non-representative populations) or if the study did not provide methodological details for evaluation. In a small number

of cases, a data point was considered to be an outlier candidate if the level was implausibly low or high based on expert judgement and data from other country data.

Data extraction

Where possible, individual-level data on blood pressure estimates were extracted from survey microdata. These data points were collapsed across demographic groupings to produce mean estimates in the standard GBD five-year age-sex groups. If microdata were unavailable, information from survey reports or from literature were extracted along with any available measure of uncertainty including standard error, uncertainty interval, and sample size. Standard deviations were also extracted. Where mean systolic blood pressure was reported split out by groups other than age, sex, location, and year (e.g. by hypertensive status), a weighted mean was calculated.

Incorporating United States prevalence data

Survey reports and literature often report information only about the prevalence, but not the level, of hypertension in the population studied. These sources were not used to model systolic blood pressure, with the exception of data from the Behavioral Risk Factors Surveillance System (BRFSS) because of the availability of a similarly structured exam survey that is representative of the same population (NHANES). BRFSS is a telephone survey conducted in the United States for all US counties. It collects self-reported diagnosis of hypertension. These self-reported values of prevalence of raised blood pressure were adjusted for self-report bias and tabulated by age group, sex, US state, and year. These prevalence values were used to predict a mean systolic blood pressure for the same strata with a regression using data from the National Health and Nutrition Examination Survey, a nationally representative health examination survey of the US adult population. The regression was run separately by sex, and was specified as:

$$\mathrm{SBP}_{l,a,t,s} = \beta_0 + \beta_1 \mathrm{prev}_{l,a,t,s}$$

where $\mathrm{SBP}_{l,a,t,s}$ is the location, age, time, and sex specific mean systolic blood pressure and $\mathrm{prev}_{l,a,t,s}$ is the location, age, time, and sex specific prevalence of raised blood pressure. The coefficients for both models are reported in Table 3.

Table 3. Coefficients in the sex-specific US states blood pressure prediction models

Term	Male model	Female model
Intercept (β_0)	114.65	108.28
Prevalence (β_1)	51.86	68.87

Out of sample RMSE was used to quantify the predictive validity of the model. The regression was repeated 10 times for each sex, each time randomly holding out 20% of the data. The RMSEs from each holdout analysis were averaged to get the average out of sample RMSE. The results of this holdout analysis are reported in Table 4.

Table 4. Out of sample RMSEs of the sex-specific US states blood pressure prediction models

	Male model	Female model
Out of sample RMSE	2.37 mmHg	3.27 mmHg

Age and sex splitting

Prior to modelling, data provided in age groups wider than the GBD five-year age groups were processed using the approach outlined in Ng and colleagues.[2] Briefly, age-sex patterns was identified using 115 sources of microdata with multiple age-sex groups, and these patterns were applied to estimate age-sex-specific levels of mean systolic blood pressure from aggregated results reported in published literature or survey reports. In order to incorporate uncertainty into this process and borrow strength across age groups when constructing the age-sex pattern, we used a model with auto-regression on the change in mean SBP over age groups:

$$\mu_a = \mu_{a-1} + \omega_a$$
$$\omega_a \sim N(\omega_{a-1}, \tau)$$

Where μ_a is the mean predicted value for age group *a*, μ_{a-1} is the mean predicted value for the age group previous to age group *a*, ω_a is the difference in mean between age group *a* and age group *a-1*, ω_{a-1} is the difference between age group *a-1* and age group *a-2*, and τ is a user-input prior on how quickly the mean SBP changes for each unit

increase in age. We used a τ of 1.5 mmHg for this model. Draws of the age-sex pattern were combined with draws of the input data needing to be split in order to calculate the new variance of age-sex split data points.

Modelling

Exposure estimates were produced from 1980 to 2019 for each national and subnational location, sex, and for each five-year age group starting from 25+. As in GBD 2017, we used a spatiotemporal Gaussian process regression (ST-GPR) framework to model the mean systolic blood pressure at the location-, year-, age-, sex- level. Details of the ST-GPR method used in GBD 2019 can be found elsewhere in the appendix.

Covariate selection

The first step of the ST-GPR framework requires the creation of a linear model for predicting SBP at the location-, year-, age-, sex- level. Covariates for this model were selected in two stages. First a list of variables with an expected causal relationship with SBP was created based on significant association found within high-quality prospective cohort studies reported in the published scientific literature. The second stage in covariate selection was to test the predictive validity of every possible combination of covariates in the linear model, given the covariates selected above. This was done separately for each sex. Predictive validity was measured with out of sample root-mean-squared error.

In GBD 2016, the linear model with the lowest root-mean-squared error for each sex was then used in the ST-GPR model. Beginning in GBD 2017, we used an ensemble model of the 50 models with the lowest root-mean-squared error for each sex. This allows us to utilise covariate information from many plausible linear mixed-effects models. The 50 models were each used to predict the mean SBP for every age, sex, location, and year, and the inverse-RMSE-weighted average of this set of 50 predictions was used as the linear prior. The relative weight contributed by each covariate is plotted by sex in Figure 1.

Figure 1. Results of the ensemble linear model covariate selection

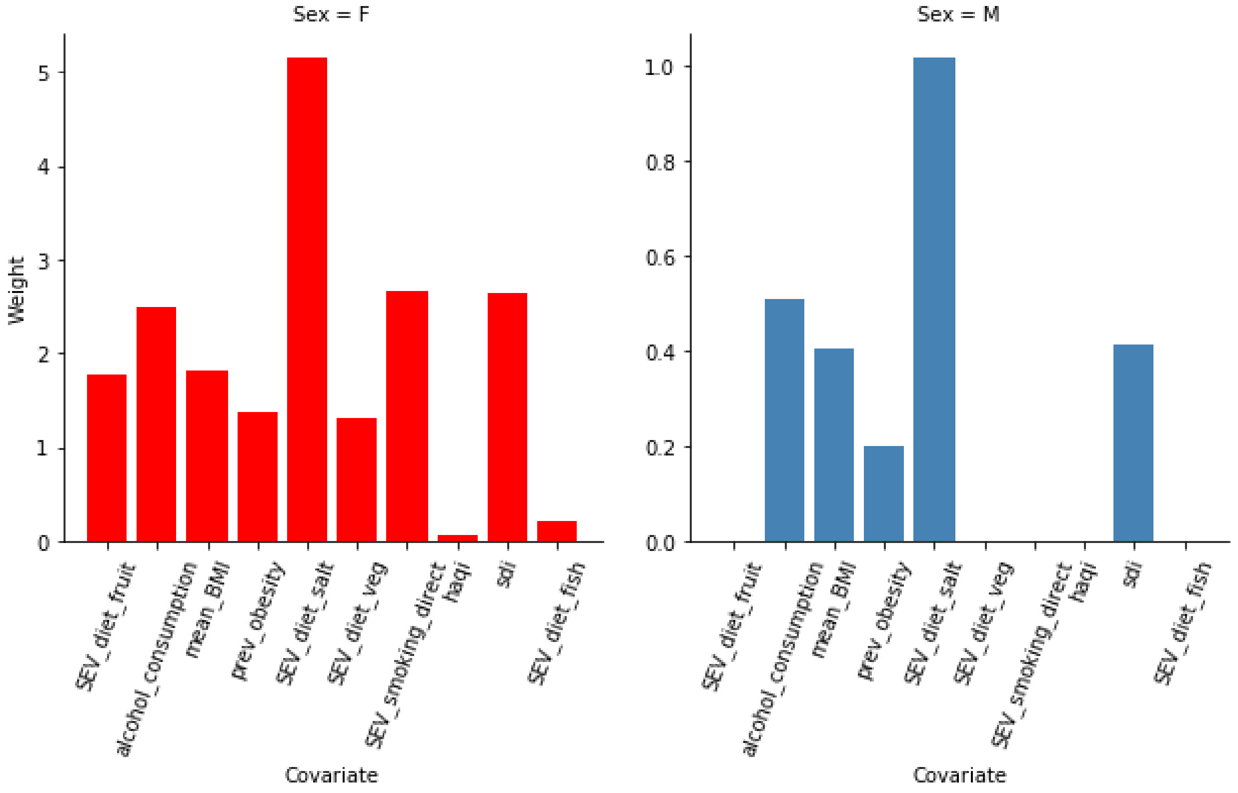

The results of the ensemble linear model were used for the first stage in an ST-GPR model. The result of the ST-GPR model are estimates of the mean SBP for each age, sex, location, and year.

Estimate of standard deviation

Currently, the ST-GPR model only produces an estimate of mean exposure level without standard deviation. Therefore, the standard deviation of systolic blood pressure within a population was estimated for each national and subnational location, sex, and five-year age group starting from age 25 using the standard deviation from person-level and some tabulated data sources. Person-level microdata accounted for 10 375 of the total 12 570 rows of data on standard deviation. The remaining 2195 rows came from tabulated data. Tabulated data were only used to model standard deviation if it was sex-specific and five-year-age-group-specific and reported a population standard deviation of systolic blood pressure. The systolic blood pressure standard deviation function was estimated using a linear regression:

$$\log\left(SD_{l,a,t,s}\right) = \beta_0 + \beta_1 \log(mean\_SBP_{l,a,t,s}) + \beta_4 sex + \sum_{k=2}^{16} \beta_k I_A$$

where $mean\_SBP_{l,a,t,s}$ is the location-, age-, time-, and sex-specific mean SBP estimate from ST-GPR, and I_A is a dummy variable for a fixed effect on a given five-year age group.

Adjustment for usual levels of blood pressure

To account for in-person variation in systolic blood pressure, a "usual blood pressure" adjustment was done. The need for this adjustment has been described elsewhere.[5] Briefly, measurements of a risk factor taken at a single time point may not accurately capture an individual's true long-term exposure to that risk. Blood pressure readings are highly variable over time due to measurement error as well as diurnal, seasonal, or biological variation. These sources of variation result in an overestimation of the variation in cross-sectional studies of the distribution of SBP.

To adjust for this overestimation, we applied a correction factor to each location-, age-, time-, and sex-specific standard deviation. These correction factors were age-specific and represented the proportion of the variation in blood pressure within a population that would be observed if there were no within-person variation across time. Four longitudinal surveys were used to estimate these factors: the China Health and Retirement Longitudinal Survey (CHRLS), the Indonesia Family Life Survey (IFLS), the National Health and Nutrition Examination Survey I Epidemiological Follow-up Study (NHANES I/EFS), and the South Africa National Income Dynamics Survey (NIDS). The sample size and number of blood pressure measurements at each measurement period for each survey is reported in Table 5.

Table 1. Characteristics of longitudinal surveys used for the usual blood pressure adjustment

Source	Measurement periods	Number of measurements	Sample size
CHRLS	2008	3	1967
	2012	3	1419
IFLS	1997	1	19 418
	2000	1	16 626
	2007	3	14 136
NIDS	1997	2	14 084
	2000	2	9612
	2007	2	9098
NHANES I/EFS	1971–1976	2	20 716
	1982–1984	3	9932

For each survey, the following regression was created for each age group:

$$SBP_{i,a} = \beta_0 + \beta_1 sex + \beta_3 age + +\upsilon_i$$

where $SBP_{i,a}$ is the systolic blood pressure of an individual i at age a, sex is a dummy variable for the sex of an individual, age is a continuous variable for the age of an individual, and υ_i is a random intercept for each individual. Then, a blood pressure value $\widehat{SBP}_{i,b}$ was predicted for each individual i for his/her age at baseline b. The correction

factor cf for each age group within each survey was calculated as variation in these predicted blood pressures was divided by the variation in the observed blood pressures at baseline, $SBP_{i,b}$:

$$cf = \sqrt{\frac{var(\widehat{SBP}_b)}{var(SBP_b)}}$$

The average of the correction factors was taken over the three surveys to get one set of age-specific correction factors, which were then multiplied by the square of the modelled standard deviations to estimate standard deviation of the "usual blood pressure" of each age, sex, location, and year. Because of low sample sizes, the correction factors for the 75–79 age group was used for all terminal age groups. The final correction factors for each age group are reported in Table 6. Figure 2 shows the correction factors by survey and age group ID.

Table 2. Age-specific usual blood pressure correction factors

Age group	Correction factor
25–29	0.665
30–34	0.713
35–39	0.737
40–44	0.733
45–49	0.798
50–54	0.771
55–59	0.764
60–64	0.753
65–69	0.719
70–74	0.689
75+	0.678

Figure 2: Correction factor by survey and age group id. The correction factor is equal to the variance of the predictions divided by the variance of the raw dataset. In pink is the average correction factor for each age group, summarised in Table 6.

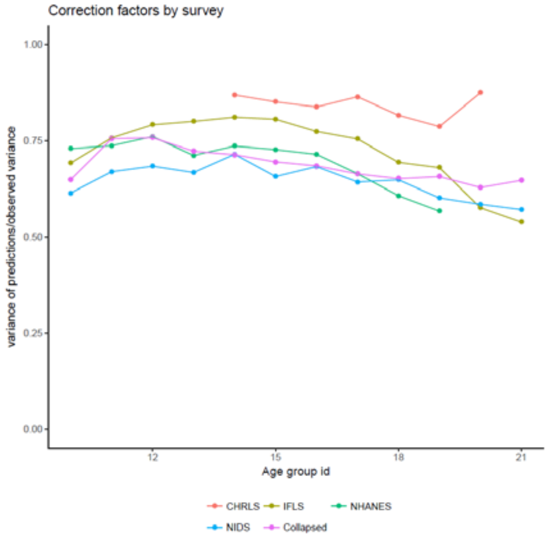

A visualisation of how the uncorrected blood pressure measurements overestimate the "usual" blood pressure variation is shown in Figure 3. This image shows the density of the distribution of the observed blood pressure

values $SBP_{i,b}$ in participants in the Indonesian Family Life Study survey in red, and the density of the predicted blood pressure values $\widehat{SBP}_{i,b}$ in blue. The ratio of the variance of the blue distribution to the variance of the red distribution is an example of the scalar adjustment factor being applied to the modelled standard deviations.

Figure 3: Raw and predicted distributions of blood pressure in the Indonesia Family Life Survey

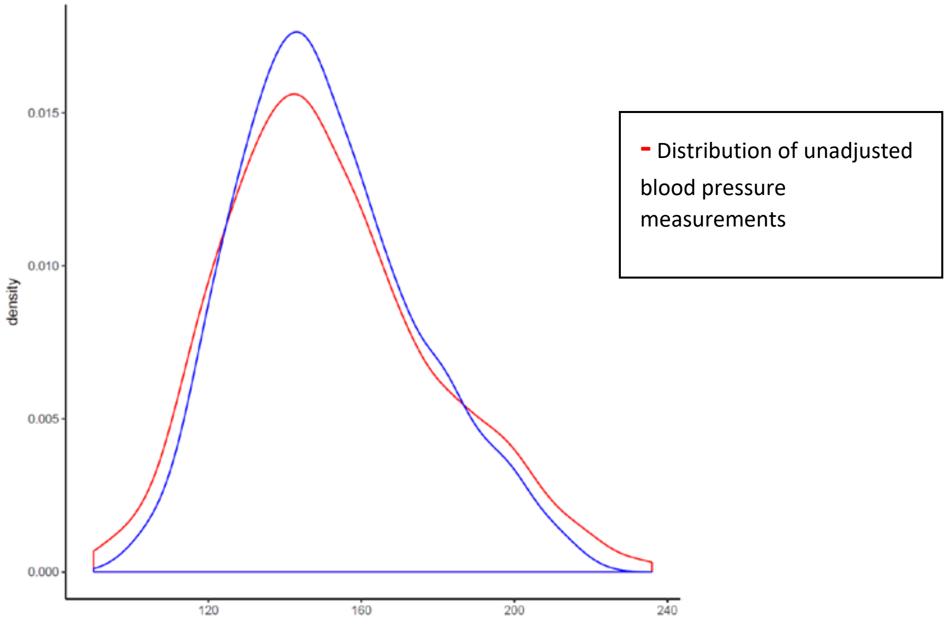

Estimating the exposure distribution shape

The shape of the distribution of systolic blood pressure was estimated using all available person-level microdata sources, which was a subset of the input data into the modelling process. The distribution shape modelling framework for GBD 2019 is detailed in the GBD 2019 Risk Factors paper. Briefly, an ensemble distribution created from a weighted average of distribution families was fit for each individual microdata source, separately by sex. The weights for the distribution families for each individual source were then averaged and weighted to create a global ensemble distribution for each sex.

Smoking estimation methods

(Adapted from "Global burden of 87 risk factors in 204 countries and territories, 1990-2019: a systematic analysis for the Global Burden of Disease Study 2019" by Murray et al., The Lancet 2020; 396: 1223–49.)

Flowchart

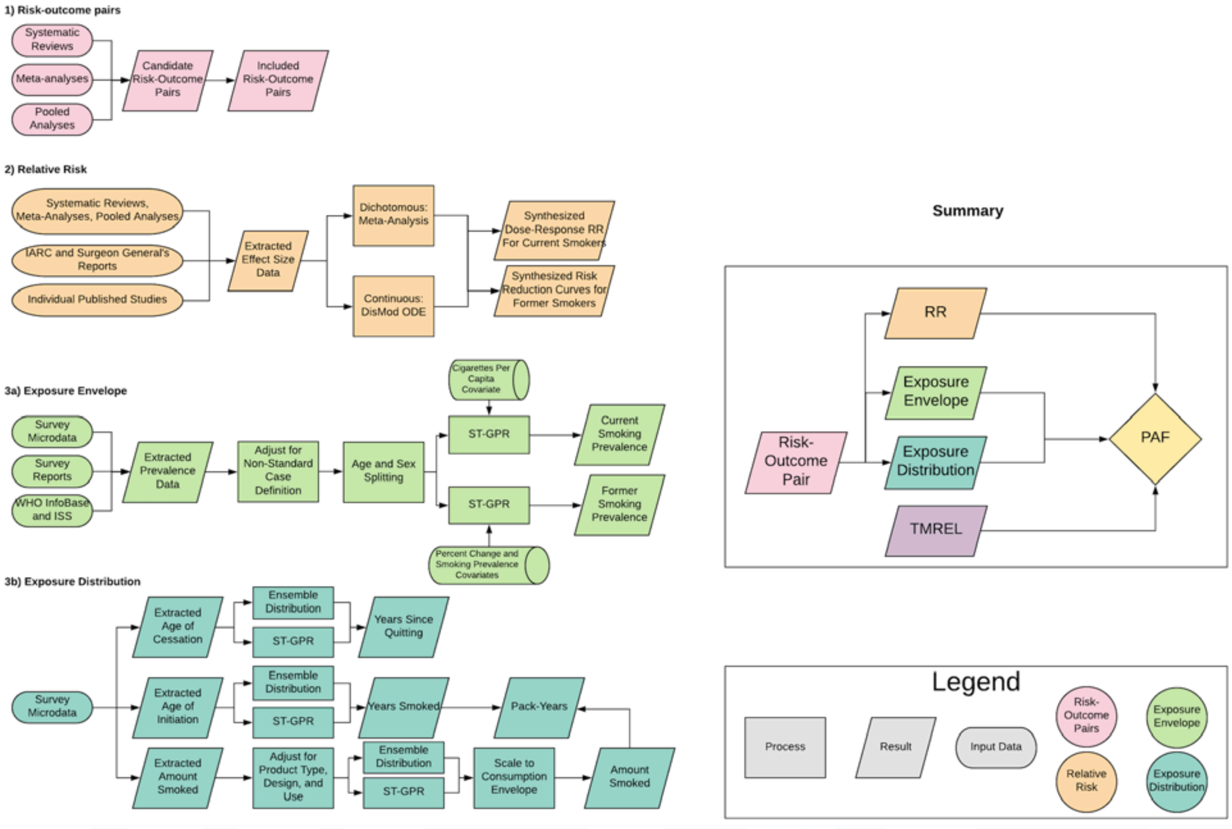

Input data and methodological summary

Definition

Exposure

As in GBD 2017, we estimated the prevalence of current smoking and the prevalence of former smoking using data from cross-sectional nationally representative household surveys. We defined current smokers as individuals who currently use any smoked tobacco product on a daily or occasional basis. We defined former smokers as individuals who quit using all smoked tobacco products for at least six months, where possible, or according to the definition used by the survey.

Input data

Our extraction method has not changed from GBD 2017. We extracted primary data from individual-level microdata and survey report tabulations. We extracted data on current, former, and/or ever smoked tobacco use reported as any combination of frequency of use (daily, occasional, and unspecified, which includes both daily and occasional smokers) and type of smoked tobacco used (all smoked tobacco, cigarettes, hookah, and other smoked tobacco products such as cigars or pipes), resulting in 36 possible combinations. Other variants of tobacco products, for example hand-rolled cigarettes, were grouped into the four type categories listed above based on product similarities.

For microdata, we extracted relevant demographic information, including age, sex, location, and year, as well as survey metadata, including survey weights, primary sampling units, and strata. This information allowed us to tabulate individual-level data in the standard GBD five-year age-sex groups and produce accurate estimates of uncertainty. For survey report tabulations, we extracted data at the most granular age-sex group provided.

Table 1: Data inputs for exposure for smoking.

Input data	Exposure
Source count (total)	3439
Number of countries with data	201

Table 2: Data inputs for relative risks for smoking.

Input data	Relative risk
Source count (total)	673
Number of countries with data	16

Crosswalk

Our GBD smoking case definitions were current smoking of any tobacco product and former smoking of any tobacco product. All other data points were adjusted to be consistent with either of these definitions. Some sources contained information on more than one case definition and these sources were used to develop the adjustment coefficient to transform alternative case definitions to the GBD case definition. The adjustment coefficient was the beta value derived from a linear model with one predictor and no intercept. We used the same crosswalk adjustment coefficients as in GBD 2017, and thus we have not included a methods explanation in this appendix, as it has been detailed previously.

Age and sex splitting

As in GBD 2017, we split data reported in broader age groups than the GBD 5-year age groups or as both sexes combined by adapting the method reported in Ng et al[1] to split using a sex- geography- time-specific reference age pattern. We separated the data into two sets: a training dataset, with data already falling into GBD sex-specific 5-year age groups, and a split dataset, which reported data in aggregated age or sex groups. We then used spatiotemporal Gaussian process regression (ST-GPR) to estimate sex-geography-time-specific age patterns using data in the training dataset. The estimated age patterns were used to split each source in the split dataset.

The ST-GPR model used to estimate the age patterns for age-sex splitting used an age weight parameter value that minimises the effect of any age smoothing. This parameter choice allowed the estimated age pattern to be driven by data, rather than being enforced by any smoothing parameters of the model. Because these age-sex split data points were to be incorporated in the final ST-GPR exposure model, we did not want to doubly enforce a modelled age pattern for a given sex-location-year on a given aggregate data point.

Modelling strategy

Smoking prevalence modelling

We used ST-GPR to model current and former smoking prevalence. The model is nearly identical to that in GBD 2017. Full details on the ST-GPR method are reported elsewhere in the appendix. Briefly, the mean function input to GPR is a complete time series of estimates generated from a mixed effects hierarchical linear model plus weighted

residuals smoothed across time, space, and age. The linear model formula for current smoking, fit separately by sex using restricted maximum likelihood in R, is:

$$logit(p_{g,a,t}) = \beta_0 + \beta_1 CPC_{g,t} + \sum_{k=2}^{19} \beta_k I_{A[a]} + \alpha_s + \alpha_r + \alpha_g + \epsilon_{g,a,t}$$

Where $CPC_{g,t}$ is the tobacco consumption covariate by geography g and time t, described above, $I_{A[a]}$ is a dummy variable indicating specific age group A that the prevalence point $p_{g,a,t}$ captures, and α_s, α_r, and α_g are super-region, region, and geography random intercepts, respectively. Random effects were used in model fitting but not in prediction.

The linear model formula for former smoking is:

$$logit(p_{g,a,t}) = \beta_0 + \beta_1 PctChange_{A[a],g,t} + \beta_3 CSP_{A[a],g,t} + \sum_{k=3}^{20} \beta_k I_{A[a]} + \alpha_s + \alpha_r + \alpha_g + \epsilon_{g,a,t}$$

Where $PctChange_{A[a],g,t}$ is the percentage change in current smoking prevalence from the previous year, and $CSP_{A[a],g,t}$ is the current smoking prevalence by specific age group A, geography g, and time t that point $p_{g,a,t}$ captures, both derived from the current smoking ST-GPR model defined above.

Supply-side estimation

The methods for modelling supply-side-level data were changed substantially from those used in GBD 2017. The raw data were domestic supply (USDA Global Surveillance Database and UN FAO) and retail supply (Euromonitor) of tobacco. Domestic supply was calculated as production + imports - exports. The data went through three rounds of outliering. First, they were age-sex split using daily smoking prevalence to generate number of cigarettes per smoker per day for a given location-age-sex-year. If more than 12 points for a particular source-location-year (equal to over 1/3 of the split points) were above the given thresholds, that source-location-year was outliered. A point would not be outliered if it was (in cigarettes per smoker): under five (10–14 year olds); under 20 (males, 15–19 year olds); under 18 (females, 15–19 year olds); under 38/35 and over three (males/females, 20+ year olds). These thresholds were chosen by visualising histograms of the data for each age-sex, as well as with expert knowledge about reasonable consumption levels. In the second round of outliering, the mean tobacco per capita value over a 10-year window was calculated. If a point was over 70% of that mean value away from the mean value, it was outliered. The 70% limit was chosen using histograms of these distances. Additionally, some manual outliering was performed to account for edge cases. Finally, data smoothing was performed by taking a three-year rolling mean over each location-year.

Next, a simple imputation to fill in missing years was performed for all series to remove compositional bias from our final estimates. Since the data from our main sources covered different time periods, by imputing a complete time series for each data series, we reduced the probability that compositional bias of the sources was leading to biased final estimates. To impute the missing years for each series, we modelled the log ratio of each pair of sources as a function of an intercept and nested random effects on super-region, region, and location. The appropriate predicted ratio was multiplied by each source that we did have, and then the predictions were averaged to get the final imputed value. For example, if source A was missing for a particular location-year, but sources B and C were present, then we predicted A twice: once from the modelled ratio of A to B, and again from the modelled ratio of A to C. These two predictions were then averaged. For some locations where there was limited overlap between series, the predicted ratio did not make sense, and a regional ratio was used.

Finally, variance was calculated both across series (within a location-year) as well as across years (within a location-source). Additionally, if a location-year had one imputed point was, the variance was multiplied by 2. If a location-year had two imputed points, the variance was multiplied by 4. The average estimates in each location-year were the

input to an ST-GPR model. For this, we used a simple mixed effects model, which was modelled in log space with nested location random effects. Subnational estimates were then further modelled by splitting the country-level estimates using current smoking prevalence.

References

1. Ng M, Freeman MK, Fleming TD, Robinson M, Dwyer-Lindgren L, Thomson B, et al. Smoking Prevalence and Cigarette Consumption in 187 Countries, 1980–2012. *JAMA*. 2014 Jan 8;311(2):183–92.

Estimation of relative risks for GBD dementia risk factors

High fasting-plasma glucose

Due to data limitations and a sparsity of sources reporting on dose-response curves for fasting plasma glucose, for GBD 2019, we used a previously published meta-analysis on the risk of Alzheimer's disease given an exposure of diabetes:

- Zhang J, Chen C, Hua S, Liao H, Wang M, Xiong Y, Cao F. An updated meta-analysis of cohort studies: diabetes and risk of Alzheimer's disease. Diabetes Res Clin Pract. 2017; Feb (124): 41-47.

Based on this study a relative risk of 1.516 (1.084 to 2.295) was used for males and a relative risk of 1.520 (1.08 to 2.301) was used for females.

High body-mass index

Relative risks for BMI were calculated using two-step generalized least squares methods for time-trend estimation methods. These methods allowed for the estimation of a log-linear relative risk curves describing the increase in the risk per additional 5 kg/m^2 units of BMI. For the relationship between BMI and dementia, this analysis leveraged information from the following data sources:

- Kivipelto M, Ngandu T, Fratiglioni L, Viitanen M, Kåreholt I, Winblad B, Helkala E-L, Tuomilehto J, Soininen H, Nissinen A. Obesity and vascular risk factors at midlife and the risk of dementia and Alzheimer disease. Arch Neurol. 2005; 62(10): 56–60
- Hassing LB, Dahl AK, Pedersen NL, Johansson B. Overweight in midlife is related to lower cognitive function 30 years later: a prospective study with longitudinal assessments. Dement Geriatr Cogn Disord. 2010; 29(6): 543-52
- Whitmer RA, Gunderson EP, Quesenberry CP, Zhou J, Yaffe K. Body Mass Index in Midlife and Risk of Alzheimer Disease and Vascular Dementia. Curr Alzheimer Res. 2007; 4(2): 103–9
- Luchsinger JA, Patel B, Tang M-X, Schupf N, Mayeux R. Measures of adiposity and dementia risk in elderly persons. Arch Neurol. 2007; 64(3): 392–8
- Hayden KM, Zandi PP, Lyketsos CG, Khachaturian AS, Bastian LA, Charoonruk G, Tschanz JT, Norton MC, Pieper CF, Munger RG, Breitner JCS, Welsh-Bohmer KA, Cache County Investigators. Vascular risk factors for incident Alzheimer disease and vascular dementia: the Cache County study. Alzheimer Dis Assoc Disord. 2006; 20(2): 93–100
- Xu W, Qiu C, Winblad B, Fratiglioni L. The Effect of Borderline Diabetes on the Risk of Dementia and Alzheimer's Disease. Diabetes. 2007; 56(1): 211–6

Based on these sources, we estimated relative risks of 1.218 (1.054 to 1.409) in males and 1.214 (1.047 to 1.404) in females per additional 5 kg/m^2 of BMI.

Smoking

Relative risk curves for smoking were estimated using the DisMod ordinary differential equations Bayesian meta-regression model. The methods allowed for the estimation of a non-linear relationship between smoking exposure and relative risk. For the relationship between smoking and dementia, this analysis leveraged information from the following data sources:

- Ohara T, Ninomiya T, Hata J, Ozawa M, Yoshida D, Mukai N, Nagata M, Iwaki T, Kitazono T, Kanba S, Kiyohara Y. Midlife and Late-Life Smoking and Risk of Dementia in the Community: The Hisayama Study. J Am Geriatr Soc. 2015; 63(11): 2332–9

- Rusanen M, Kivipelto M, Quesenberry CP, Zhou J, Whitmer RA. Heavy smoking in midlife and long-term risk of Alzheimer disease and vascular dementia. Arch Intern Med. 2011; 171(4): 333–9
- Ikeda A, Yamagishi K, Tanigawa T, Cui R, Yao M, Noda H, Umesawa M, Chei C, Yokota K, Shiina Y, Harada M, Murata K, Asada T, Shimamoto T, Iso H. Cigarette smoking and risk of disabling dementia in a Japanese rural community: a nested case-control study. Cerebrovasc Dis. 2008; 25(4): 324-31
- Prince M, Cullen M, Mann A. Risk factors for Alzheimer's disease and dementia: a case-control study based on the MRC elderly hypertension trial. Neurology. 1994; 44(1): 97-104
- Garcia AM, Ramon-Bou N, Porta M. Isolated and joint effects of tobacco and alcohol consumption on risk of Alzheimer's disease. J Alzheimers Dis. 2010; 20(2): 577-86
- Shalat SL, Seltzer B, Pidcock C, Baker EL Jr. Risk factors for Alzheimer's disease: a case-control study. Neurology. 1987; 37(10): 1630-3

Based on the information in these sources, the estimated relative risks for all ages and both sexes were the following:

Cigarette Equivalent	Relative Risk
0	1.00 (1.00-1.00)
12	2.08 (1.40–2.84)
24	2.94 (1.78–4.47)
36	3.74 (1.97–6.02)
48	4.10 (2.07–7.23)

GBD world regions

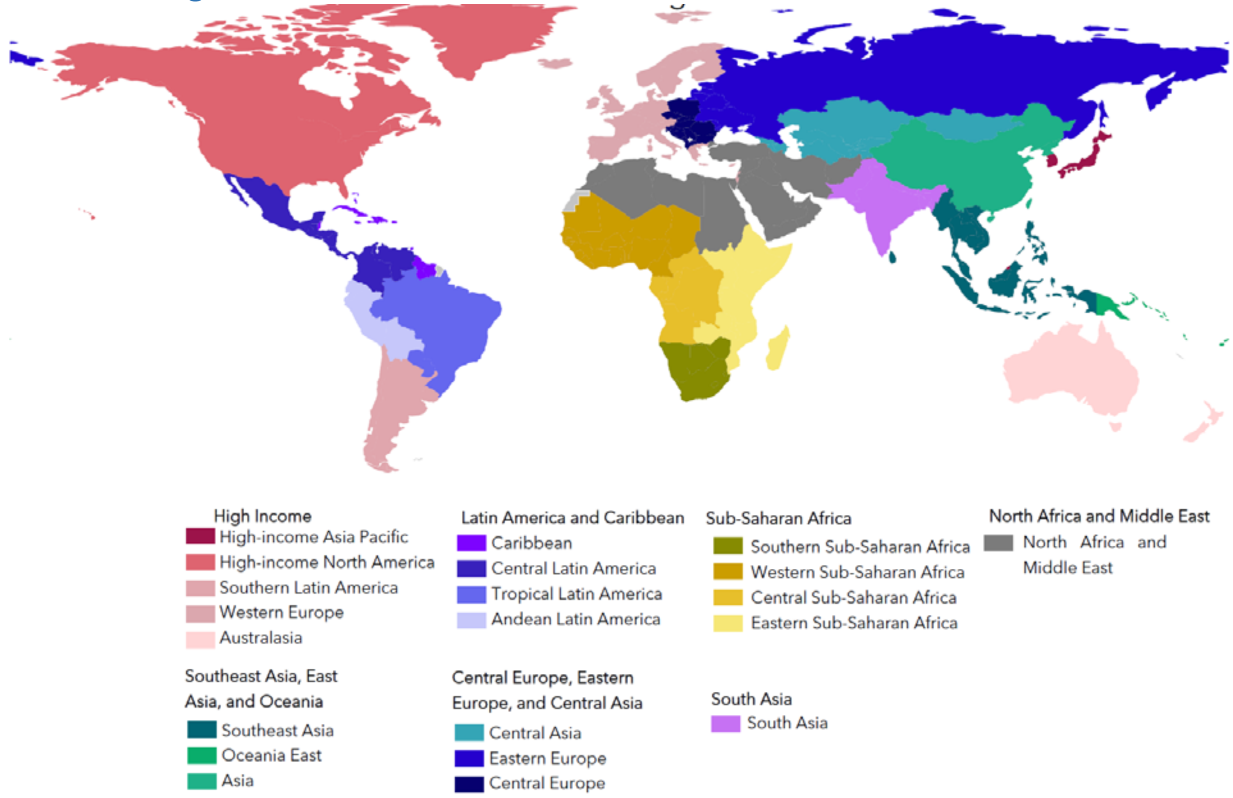

Supplemental Figure S1

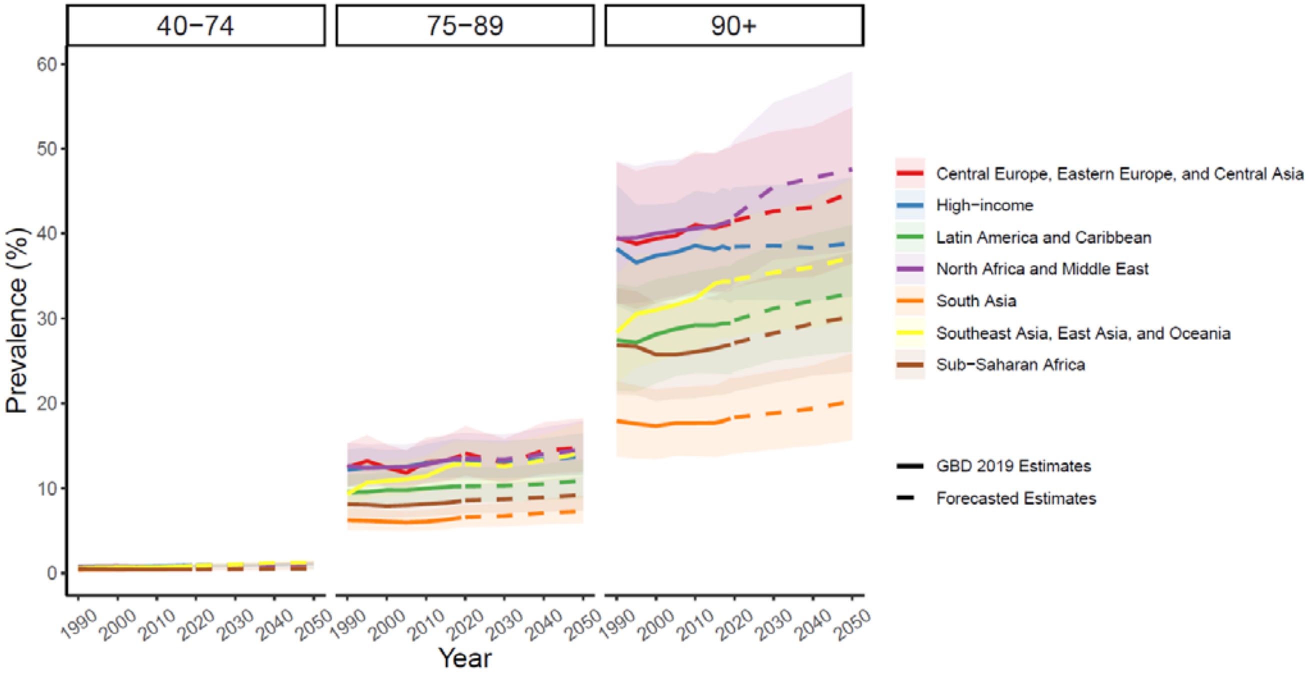

Figure S1. Both-sex prevalence rates by GBD super-region from 1990 to 2050 by age group

Supplemental Figure S2

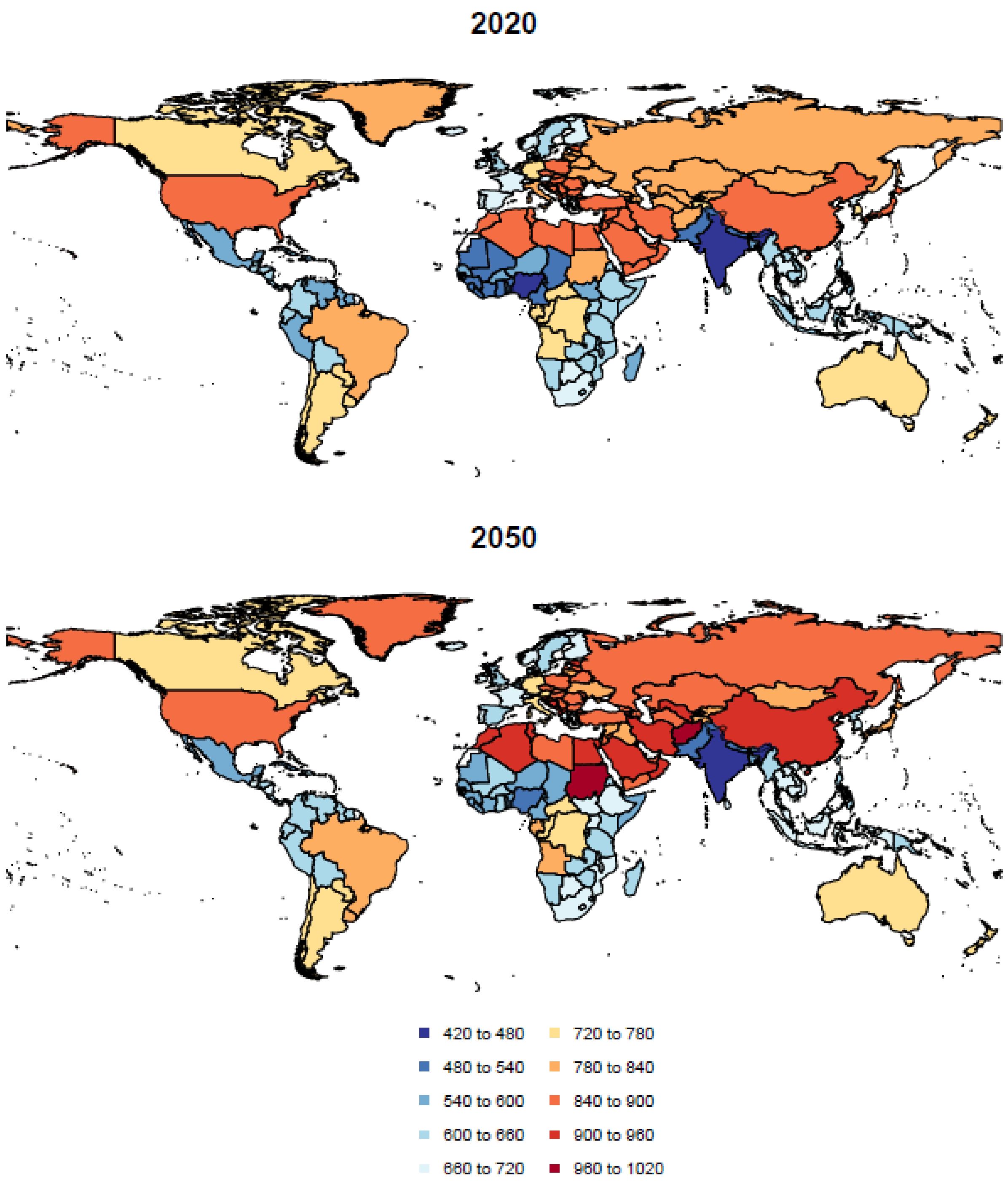

Figure S2. Comparison of both-sex age-standardised dementia prevalence (per 100,000) by country in 2020 and 2050
